# Benzo‐fused Tri[8]annulenes as Molecular Models of Cubic Graphite

**DOI:** 10.1002/anie.202106233

**Published:** 2021-08-06

**Authors:** Barbara Ejlli, Pascal Nußbaum, Frank Rominger, Jan Freudenberg, Uwe H. F. Bunz, Klaus Müllen

**Affiliations:** ^1^ Organisch-Chemisches Institut Ruprecht-Karls-Universität Heidelberg Im Neuenheimer Feld 270 69120 Heidelberg Germany; ^2^ InnovationLab Speyerer Str. 4 69115 Heidelberg Germany; ^3^ Max Planck Institute for Polymer Research Ackermannweg 10 55128 Mainz Germany

**Keywords:** carbon allotrope, cubic graphite, cyclooctatetraene, cyclotrimerization, molecular cup

## Abstract

Cyclotrimerization of 9,10‐dibromo‐9,10‐dihydrodibenzo[3,4:7,8]cycloocta[1,2‐*l*]phenanthrene with potassium *tert*‐butoxide in the presence of a transition‐metal catalyst afforded two polycyclic aromatic hydrocarbon stereoisomers consisting of three cyclooctatetraene (COT) moieties connected via a central benzene ring. Both isomeric tri[8]annulenes were obtained selectively through the choice of the catalyst: The α,α,α‐form (Ru catalyst) displayed a threefold symmetrywith the COT subunits forming the side walls of a (chiral) molecular cup. In the thermodynamically more stable α,α,β‐isomer (Pd catalyst), one of the three boat‐shaped COTs was flipped over and faced the opposite molecular hemisphere with respect to the central benzene ring as evidenced by crystal structure analysis. Both title compounds are small segments of “cubic graphite”, an elusive carbon allotrope.

## Graphical abstract

Two benzo‐fused tri[8]annulenes were synthesized via cyclotrimerization reactions, introducing two valid model compounds of the yet elusive carbon allotrope cubic graphite. Both conformers do not interconvert via temperature‐ or light‐induced stimuli.

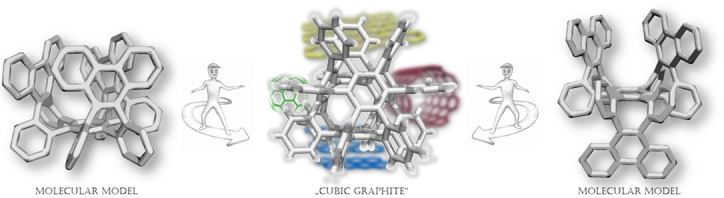

The discovery of carbon allotropes^[1]^ such as fullerenes,^[2]^ carbon nanotubes,^[3]^ graphenes^[4]^ and, most recently, other carbon nanostructures synthesized via on‐surface chemistry^[5]^ has attracted great attention.^[6]^ While the well‐known forms of carbon include diamond^[7]^ and graphite,^[8]^ there is another, yet elusive 3D carbon allotrope, the so‐called “cubic graphite”, deriving its name from its symmetry and relationship to conventional graphite. It was first proposed by Gibson et al.^[9]^ and predicted to possess high thermal and mechanical stability.^[10]^ Attempted synthesis by high‐temperature carbonization of hexahalobenzenes with sodium amalgam only furnished amorphous carbon, illustrating the difficulty in accessing the three‐dimensional, crowded arrangement of hexaphenylbenzene units.^[9]^ Cubic graphite can be regarded as a 3D carbon structure[^10^, ^11^] consisting of hexagons and octagons: Each benzene ring is part of three equivalent poly‐*para*‐phenylene (PPP) chains while two biphenyl subunits of neighboring PPP strands form eight‐membered[^6k^, ^12^] tetraphenylene rings, rendering all of the sp^2^ hybridized carbon atoms equivalent. If ever prepared, the existence of diffusion channels, for example, for lithium ions, should qualify cubic graphite as active material for rechargeable batteries.[^10^, ^11^]

Subunits of cubic graphite were prepared via bottom‐up approaches to serve as model compounds.[^11b^, ^13^] Three‐dimensional dendritic oligophenylenes^[13b]^ underline the crowded oligophenylenic nature while the tetraphenylene substructures^[14]^ are lacking entirely. The latter are present in the doubly helical *ortho*‐oligophenylenes[^14c^, ^15^] although the 3D feature is missing in the linear arrangements.^[16]^ Synthetic strategies towards these models involve Suzuki couplings^[13a]^ and Diels–Alder‐ cycloadditions.[^11b^, ^13b^] Tetraphenylene substructures^[10]^ were synthesized via thermal ring opening of biphenylenes^[17]^ or via Suzuki^[18]^ or Ullmann^[15c]^ reactions of biphenyls, albeit in low yields.

Hypothetically, cubic graphite (Figure 1) would be available by multiple cyclotetramerizations of hexadehydrobenzene or cyclotrimerizations of octadehydrocyclooctatetraene. Therefore, we envisage a suitable model compound to result from cyclotrimerization of a cyclooctatrienyne with sterically congested benzene rings.

**Figure 1:**
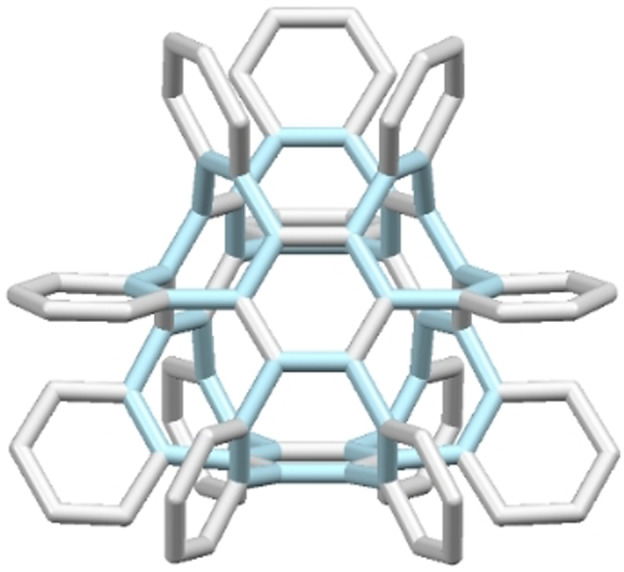
A hypothetical segment of cubic graphite (eight‐membered‐rings are hightlighted in blue).

In this contribution, we report two routes towards hydrocarbon **7**, a subunit of cubic graphite, which rely on cyclotrimerization reactions (Scheme 1). We expected the less crowded C_s_‐symmetric (α,α,β)‐conformer **7 a** to be favored over the (α,α,α)‐rotamer **7 b**, where the COT rings all point toward the same side of the central benzene ring.

**Scheme 1:**
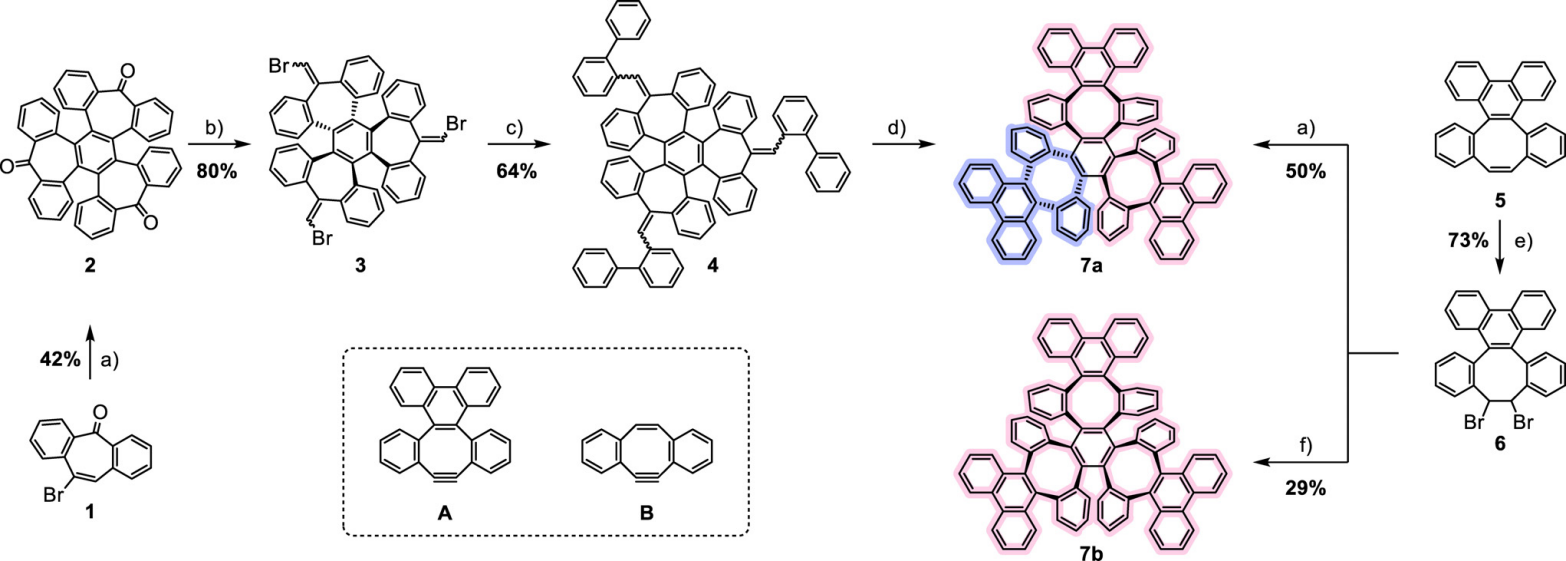
Possible synthetic approaches towards cyclotrimer **7 a**,**b** and strained cycloalkynes discussed herein. Reagents and conditions: a) KO^*t*^Bu, Pd(dba)_2_, PPh_3_, toluene, 100 °C, 3 d, 42 % yield **2**, 50 % yield **7 a**; b) (bromomethyl)triphenylphosphoniumbromide, NaHMDS, Et_2_O, −78 °C—rt, 16 h, 80 % yield; c) 2‐biphenylboronic acid, Pd(PPh_3_)_4_, K_2_CO_3_, THF/H_2_O, 75 °C, 16 h, 64 % yield; d) DDQ, Cu(OTf)_2_, chloroform, up to 150 °C, microwave, up to 48 h; e) Br_2_, CHCl_3_, 75 °C, 16 h, 73 % yield; f) [(C_5_H_5_)Ru(CH_3_CN)_3_]PF_6_, KO^*t*^Bu, DCM, rt, 2 d, 29 % yield **7 b**. Relative stereochemistry depicted.

In a first attempt, brominated dibenzosuberenone **1** was subjected to Pd catalyzed cyclotrimerization in the presence of triphenylphosphine under basic conditions to furnish **2** in 42 % yield. Benzannulated “tris‐tropone” **2**, previously prepared in Ar matrices^[19]^ or via demetallation of a platinum‐alkyne complex,^[20]^ thus became available on the gram scale. As its ring expansion^[21]^ failed, **2** was transformed into bromovinyl derivative **3** after Wittig reaction in 80 % yield. Subsequent Suzuki coupling with 2‐biphenylboronic acid furnished the hydrocarbon **4** (64 % yield), which was then subjected to an established tandem oxidative ring expansion in the presence of Cu(OTf)_2_.^[22]^ Even reactions at 150 °C in a pressurized microwave reactor for 48 h only resulted in traces of compound **7 a** among its hard‐to‐separate intermediates (see Supporting Information, section 2.1).

Compound **7 a** was, however, accessed via an alternative route: Bromination of dibenzo[3,4:7,8]cycloocta[1,2‐*l*]phenanthrene (**5**)^[22]^ led to dibromo derivative **6**. Double HBr elimination with KO^*t*^Bu generated the corresponding cyclooctatrienyne **A**, which cyclotrimerized in situ at 100 °C in the presence of Pd(dba)_2_ as catalyst and triphenylphosphine as ligand to furnish **7 a** in a yield of 50 % (at room temperature, only debromination was observed). The proton NMR spectrum of **7 a** (Figure 2, top) exhibited three characteristic downfield‐shifted resonances of the three different phenanthrenylene bay protons (*1*, *2* and *3*) at chemical shifts exceeding 8.5 ppm (phenanthrene: 8.75 ppm in CDCl_3_).^[23]^

**Figure 2:**
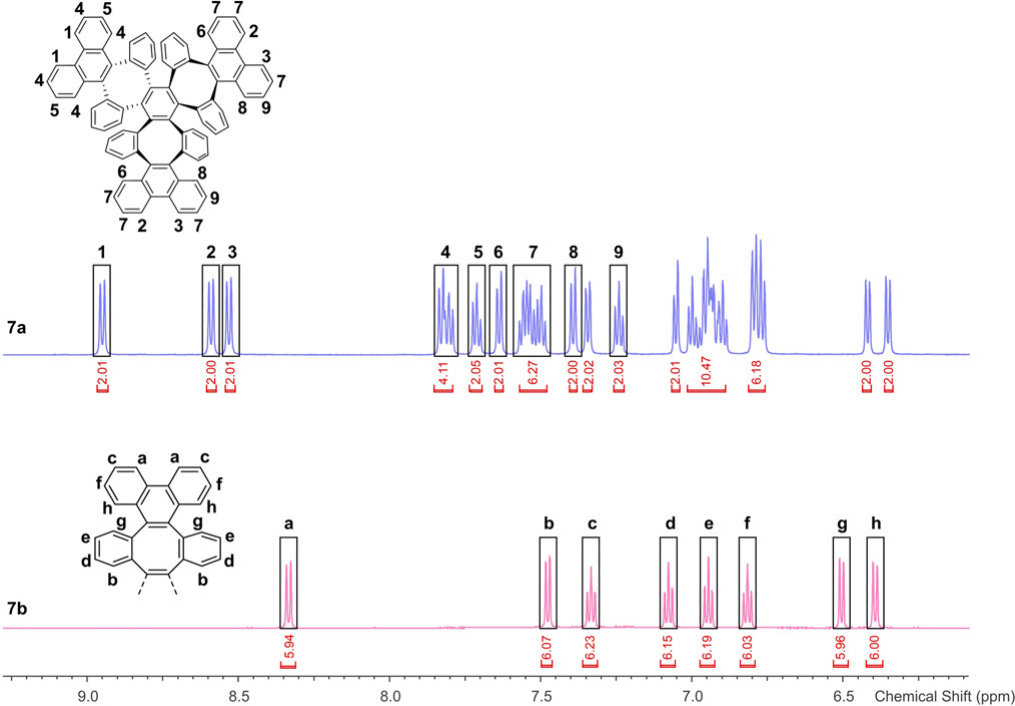
Aromatic region of the ^1^H NMR spectra of **7 a** (top, 600 MHz) and **7 b** (bottom, 600 MHz) in CD_2_Cl_2_.

Strikingly, treatment of **6** under basic conditions in the presence of [(C_5_H_5_)Ru(CH_3_CN)_3_]PF_6_ at room temperature furnished a different cyclotrimer in 29 % yield, albeit with a NMR spectrum suggesting a C_3_ symmetry with only one resonance for the phenanthrene bay protons (relative intensity of 6 for protons *a*; Figure 2, bottom)—we identified the second cyclotrimer as the (α,α,α)‐isomer **7 b**. When attempting olefin metathesis under similar conditions at room temperature, Nuckolls et al.^[24]^ crystallized the respective (α,α,β)‐cyclotrimer with 12 benzene rings less than **7 a,b** (Scheme 1) as a discrete by‐product, starting from **B**. By contrast, we only obtained the (α,α,β)‐isomer **7 a** upon Pd catalysis at 100 °C. These opposing selectivities may be a consequence of the different steric demands of the intermediately generated cycloalkynes **A** (boat‐shaped) and **B** (approximately planar) at the catalytic center. At 100 °C under Ru‐catalysis, selectivity is inverted and the more stable (α,α,β)‐cyclotrimer **7 a** is formed, albeit in only trace amounts among several decomposition products. Note that both new cyclotrimers **7 a** and **7 b** formed selectively through catalyst and temperature control.

Both isomers failed to show thermal or photochemical interconversion: Temperature‐dependent ^1^H NMR spectroscopy in tetrachloroethane from −35 °C to 70 °C exhibited slight downfield shifts of the resonances with increasing temperature, but did not indicate interconversion (see Supporting Information for details). Thermal isomerization was also not observed after keeping neat **7 a** and **7 b** at 400 °C for 10 minutes. Photoirradiation of solutions of **7 a** and **7 b** in cyclohexane in a Rayonet photoreactor (300 nm) under inert atmosphere for 24 h provided only starting materials.

Ring inversion of benzannulated cyclooctatetraenes occurs via planarization^[25]^ or a twisted, non‐planar transition state^[26]^ (symmetry reduction), which minimizes the *ortho*, *ortho′* hydrogen interactions—however the barrier remains as high as 75.8 kcal mol^−1^ for (in comparison to compounds **7 a**,**b** more flexible and less crowded) tetraphenylene.^[26c]^

A simple molecular model of **7 a**,**b** (see Supporting Information) with one manually planarized eight‐membered ring illustrates the repulsion of neighboring protons (*b*, *g*, *h* etc.) in the fjord regions and supports the difficulty of interconverting **7 a**,**b**. Density functional calculations (DFT, B3LYP/6–311G**) show that the (α,α,β)‐isomer **7 a** is 16 kcal mol^−1^ more stable than the (α,α,α)‐isomer **7 b** (see Supporting Information, section 2.7 for details).

The absorption spectra of **7 a**/**7 b** were essentially indistinguishable as expected for these conformers (see Supporting Information, Figure S38). The least intense absorption features at long wavelengths (*λ*_max,abs_=356/358 nm) were similar to those of phenanthrene,^[27]^ the largest π‐conjugated subunit in both compounds—COT‐annulation did not lead to a pronounced red‐shift.^[28]^ **7 a** and **7 b** fluoresced violet‐blue at *λ*_max,em_=360/365 nm.^[29]^

Single crystal analysis of **7 a** unambiguously confirmed the formation of the π‐extended tri[8]annulene consisting of three boat‐shaped COT moieties. One of them faces the opposite molecular hemisphere with respect to the central benzene ring compared to the other two (Figure 3, left). The phenanthrene moieties are oriented nearly perpendicular (80.4–91.3°) to the benzene core. These angles deviate from those spanned by the opposing phenylenes in tetrabenzocyclooctatetraene (84.4–85.1°),^[30]^ which is also observed in case of the single crystals of **7 b** (87.1–98.5°).

**Figure 3:**
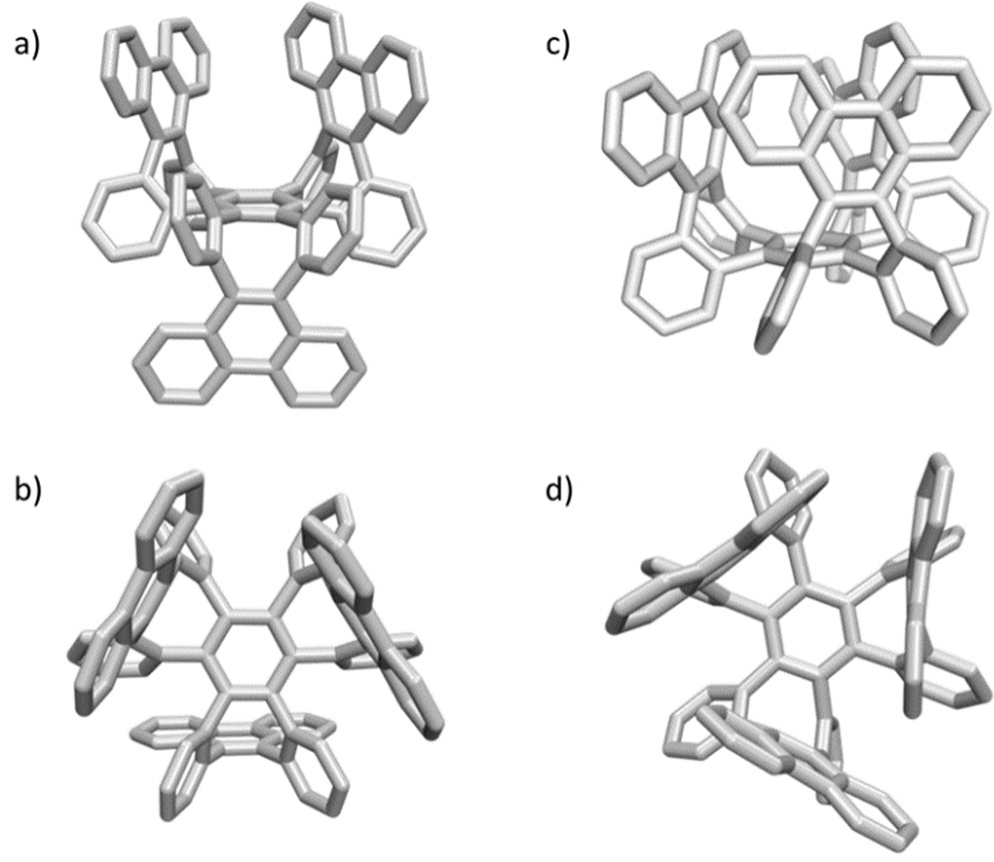
Side view of the single crystal X‐ray structure of **7 a** (a) and **7 b** (c). Top view, illustrating saddle‐shaped **7 a** (b) and the cavity within **7 b** (d) (for Packing see Supporting Information). Solvent molecules were omitted for clarity.^[31]^

Here, however, all three boat‐shaped eight‐membered rings face the same side with respect to the central benzene ring (Figure 3, right). The COT subunits form the sidewalls of chiral molecular cups—λ‐ and δ‐enantiomers are present in the crystal due to the head‐to‐face alignment of the non‐planar phenanthrylenes. The resulting triangle‐shaped cavities should be able to host a suitable alkali cation upon reduction, as their diameter is about 2.2 Å (Method 1) and 2.7 Å (Method 2) (see Supporting Information, section 2.6 for details), illustrating a feature of cubic graphite.

Both **7 a** and **7 b** are thus valid three‐dimensional model compounds of cubic graphite (cf. Figure 1). They consist of sterically congested alternating benzene and cyclooctatetraene rings. Whereas **7 a** illustrates the expansion in three‐dimensional space slightly better due to the different orientation of the boat‐shaped COTs, **7 b** models the cavities within cubic graphite.

In conclusion, we synthesized the two non‐interconverting conformers **7 a** and **7 b** as model compounds of cubic graphite in a selective fashion through catalyst control from an in situ generated strained cycloalkyne **A**. An alternative strategy via ring expansion by oxidative rearrangements of seven‐membered rings was not successful. Yet, a simple gram scale synthetic route to benzannulated “tris‐tropone” **2** became possible, a highly attractive building block for negatively curved polycyclic hydrocarbons. Future challenges include i) π‐extension of model compounds **7 a**, **b** toward higher homologues, ii) electron transfer reactions to explore the resulting anion structures and iii) host‐guest chemistry of the “molecular cup” **7 b**. A quantum‐chemical study of the cyclotrimerization reaction mechanisms involving **A** (and **B**) may provide mechanistic insights into the surprising selectivities.

## Conflict of interest

The authors declare no conflict of interest.

## Supporting information

As a service to our authors and readers, this journal provides supporting information supplied by the authors. Such materials are peer reviewed and may be re‐organized for online delivery, but are not copy‐edited or typeset. Technical support issues arising from supporting information (other than missing files) should be addressed to the authors.

Supporting Information
