# Supplementary material for: Benzo‐fused Tri[8]annulenes as Molecular Models of Cubic Graphite
Source: Angew Chem Int Ed Engl. 2021 Aug 6;60(37):20220–4. doi: 10.1002/anie.202106233 (PMC8457115; doi:10.1002/anie.202106233)
Supplement: Supplementary file 1 — Supporting Information [file ANIE-60-20220-s001.pdf]

Angewandte Chemie
Eine Zeitschrift der Gesellschaft Deutscher Chemiker

Supporting Information

Benzo-fused Tri[8]annulenes as Molecular Models of Cubic Graphite

Barbara Ejlli, Pascal Nußbaum, Frank Rominger, Jan Freudenberg, Uwe H. F. Bunz, and Klaus Müllen**

anie_202106233_sm_miscellaneous_information.pdf

Table of Contents

1. Experimental Procedures 1

1.1 General Remarks 1

1.2 Synthesis 3

2. Results and Discussion 14

2.1 Mass Spectrometry 14

2.2 NMR Spectroscopy 17

2.3 Temperature-Dependent ^{1}H-NMR studies 32

2.4 Absorption and Emission Spectra 34

2.5 Thermal and Light-Induced Stimuli 34

2.6 Determination of the Cavity Volume within **7b** 35

2.7 Calculations 37

2.8 Crystallographic Data 44

References 48

SUPPORTING INFORMATION

1. Experimental Procedures

1.1 General Remarks

Reagents and Solvents for Synthesis

All reagents and solvents were obtained from commercial suppliers (SigmaAldrich Laborchemikalien GmbH, TCI Deutschland GmbH and abcr GmbH) and used without further purification. Deuterated solvents for NMR analysis were purchased from SigmaAldrich Laborchemikalien GmbH. Absolute solvents were used directly from a solvent system (MB SPS-800) containing sufficient drying agents.

Column Chromatography

Flash column chromatography was carried out using silica gel (grain size 0.04 - 0.063 mm) produced by SigmaAldrich GmbH. As mobile phase the solvents named in the synthetic procedure were used. For thin layer chromatography Polygram Sil g/UV 254 plates from Macherey Nagel were used and examined under UV-light irradiation (254 nm and 365 nm).

Nuclear Magnetic Resonance Spectroscopy

All NMR spectra were recorded in deuterated solvents ($CDCl_3$, CD_2Cl_2, DMSO-d_6 and $C_2D_2Cl_4$) at room temperature (if not stated otherwise) on a Bruker Avance III (300 MHz), Bruker Avance III (400 MHz), Bruker Avance III (500 MHz), Bruker Avance III (600 MHz) or Bruker Avance III (700 MHz). ^{13}C NMR spectra were measured proton decoupled if not stated otherwise. Chemical shifts δ are reported in part per million (ppm) and coupling constants *J* in Hz. All spectra were referenced to the solvent signal.[S1] For the multiplicities, the following abbreviations are used: s = singlet, d = doublet, t = triplet, m = multiplet. The spectra were processed and integrated using ACD/Spectrus processor.

Mass Spectrometry

High-resolution mass spectra (HRMS) were obtained by matrix-assisted laser desorption ionization (MALDI) using DCTB as matrix or direct analysis in real time (DART) experiments on Bruker ApexQe hybrid 9.4 T FT-ICR or Bruker AutoFlex Speed time-of-flight spectrometers. EI^+ mass spectra were recorded on the JEOL JMS-700.

Thermogravimetric Analysis and Differential Scanning Calorimetry

Analyses were done using a Mettler Toledo TGA/DSC1 device and the ASCII files were exported and visualized by Origin Pro 2021.

IR Spectra

IR spectra were recorded on a JASCO FT/IR-4100 using the neat compounds at room temperature. The data was processed using JASCO Spectra ManagerTM II and all signals are reported in wavenumbers [cm^{-1}].

Melting Points

Melting points were determined in open glass capillaries with a Melting Point Apparatus MEL-TEMP (Electrothermal, Rochford, UK).

UV-Vis and Fluorescence Spectra

All UV-Vis spectra were recorded on a JASCO UV-Vis 660 and fluorescence spectra were recorded on a JASCO FP-6500.

Computational Studies

Computational studies were carried out using DFT/TD-DFT calculations on Turbomole 6.3.1 and Gaussian16 [S2] or Spartan 10.[S3] Geometry optimizations were performed using the B3LYP functional and def2-TZVP or def2-svp basis set. At this geometry, the absolute energy were assigned by a single-point approach at the B3LYP/6-311G** level of theory.

X-ray single-crystal structure analysis

X-ray single-crystal structure analyses were measured on a Bruker Smart APEX-II QuazarArea detector or STOE Stadivari CCD area detector diffractometer. Diffraction intensities were corrected for Lorentz and polarization effects. An empirical absorption correction was applied using SADABS[S4] based on the Laue symmetry of reciprocal space or using X-Area LANA 1.70.0.0 (STOE, 2017) based on the Laue symmetry of the reciprocal space. Hydrogen atoms were either isotropically refined or calculated. The structures were solved and refined using the SHELXT-2014 (Sheldrick 2014) [S5] and refined against F^2 with a Full-matrix least-squares algorithm using the SHELXL-2018/3 (Sheldrick, 2018) software.[S6]

Photochemical Reactor

The photoirradiation of the solutions was carried out using a Rayonet RPR-200 photoreactor with 14 watt light sources.

1.2 Synthesis

Route A

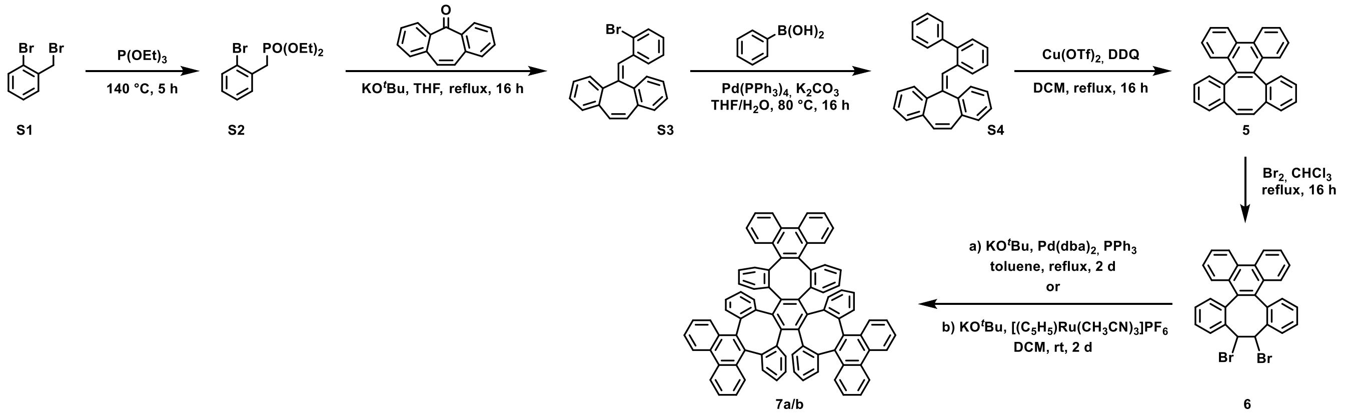

Route B

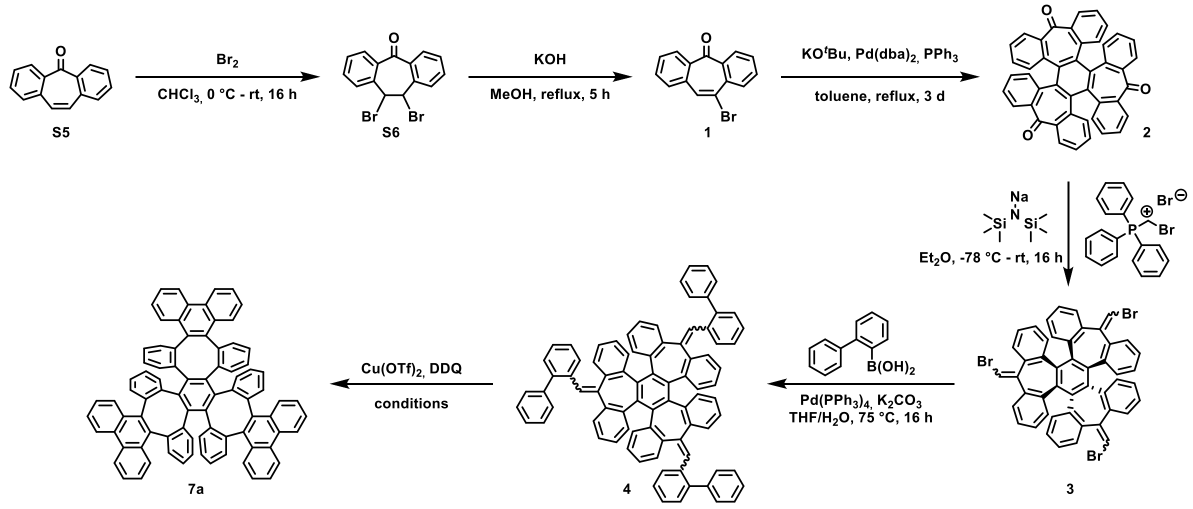

Scheme S1. Synthesis of cyclotrimers **S7a/b**, route A (top) and **S7a** route B (bottom).

Diethyl (2-bromobenzyl)phosphonate (**S2**)

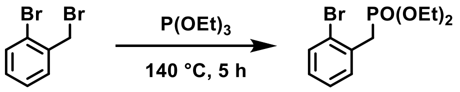

S1 S2

S2 was synthesized according to a literature known procedure.[7] A mixture of 1-bromo-2-(bromomethyl)benzene (7.70 g, 30.8 mmol, 1.00 eq.) and triethyl phosphite (5.55 mL, 5.37 g, 32.3 mmol, 1.05 eq.) was heated at 140 °C for 5 h. Surplus triethyl phosphite was removed under reduced pressure and the resulting diethyl (2-bromobenzyl)phosphonate **S2** (9.40 g, 30.6 mmol, 99%) was used directly to the next step without further purification.

5-(2-Bromobenzylidene)-5*H*-dibenzo[*a,d*][7]annulene (**S3**)

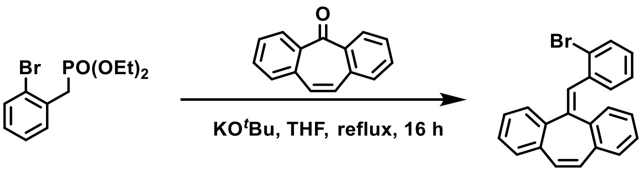

S2 S3

In a heatgun dried Schlenk tube under an atmosphere of argon **S2** (9.40 g, 30.6 mmol, 1.00 eq.) and dibenzosuberenone (6.31 g, 30.6 mmol, 1.00 eq.) were dissolved in 120 mL anhydrous tetrahydrofuran. Potassium *tert*-butoxide solution (1 mol L^{-1} in tetrahydrofuran, 45.9 mL, 45.9 mmol, 1.50 eq.) was added dropwise and the reaction mixture was stirred at 70 °C overnight. The resulting reaction mixture was filtered through a plug of silica and thereafter tetrahydrofuran was removed under reduced pressure. The residue was purified by column chromatography (SiO_2) using petroleum ether/ethyl acetate (10:1) as eluent. The product **S3** was isolated as a colorless solid (8.27 g, 22.9 mmol, 75%).

R$_f$ = 0.68 (SiO_2; petroleum ether/ethyl acetate 10:1).

^{1}H NMR ($CDCl_3$, 300 MHz, rt): δ = 7.65 (d, *J*=7.36 Hz, 1H), 7.55 (dd, ^4J=1.26 Hz, ^3J=7.87 Hz 1H), 7.43-7.48 (m, 1H), 7.33-7.37 (m, 3H), 7.18-7.22 (t, *J*=7.50 Hz, 1H), 7.07 (t, *J*=7.50 Hz 1H), 6.87-7.01 (m, 5H), 6.67 (s, 1H), 6.57 (dd, ^4J=1.72 Hz, ^3J=7.68 Hz 1H) ppm.

^{13}C {^{1}H} NMR ($CDCl_3$, 75 MHz, rt): δ = 144.2, 137.8, 137.29, 135.4, 132.8, 131.8, 131.5, 129.7, 129.0, 128.8, 128.8, 127.7, 127.6, 126.9, 125.0 ppm.

Data in accordance with the literature.[S7]

5-([1,1'-Biphenyl]-2-ylmethylene)-5*H*-dibenzo[*a,d*][7]annulene (**S4**)

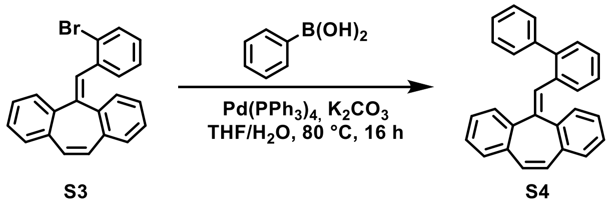

In a heatgun dried Schlenk tube under an atmosphere of argon a mixture of **S3** (1.00 g, 2.78 mmol, 1.00 eq.) and phenylboronic acid (510 mg, 4.18 mmol, 1.50 eq.) was dissolved in 10 mL freshly degassed tetrahydrofuran/water (4:1). Then, $Pd(PPh_3)_4$ (160 mg, 140 µmol, 5 mol%.) and K_2CO_3 (770 mg, 5.57 mmol, 2.00 eq.) were added, and the reaction mixture was stirred at 80 °C overnight. The mixture was cooled to room temperature, filtered through a plug of silica and the solvent was removed under reduced pressure. After flash column chromatography (SiO_2; petroleum ether) the product **S4** was isolated as a colorless solid (910 mg, 2.53 mmol, 91%).

R_f = 0.62 (SiO_2; petroleum ether/ethyl acetate 9:1).

^{1}H NMR (DMSO-d_6, 400 MHz, rt): δ = 7.57-7.58 (m, 4H), 7.38-7.46 (m, 2H), 7.22-7.36 (m, 8H), 7.13-7.15 (m, 1H), 6.92-7.03 (m, 3H), 6.56-6.58 (m, 1H), 6.22 (s, 1H) ppm.

^{13}C {^{1}H} NMR (DMSO-d_6, 101 MHz, rt): δ = 141.9, 141.4, 140.8, 140.6, 136.9, 135.1, 134.53, 134.2, 132.6, 131.3, 131.2, 129.5, 129.2, 129.0, 128.9, 128.8, 128.6, 128.5, 128.1, 127.7, 127.4, 126.8 ppm.

Data in accordance with the literature.[S7]

(*Z*)-Dibenzo[3,4:7,8]cycloocta[1,2-*l*]phenanthrene (**5**)

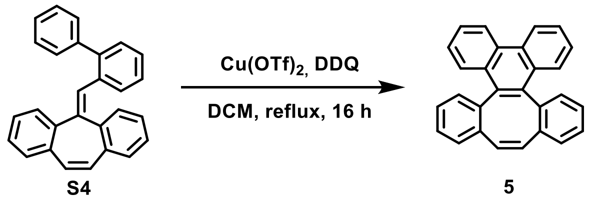

In a heatgun dried Schlenk tube under an atmosphere of argon **S4** (500 mg, 1.40 mmol, 1.00 eq.), DDQ (790 mg, 3.51 mmol, 2.50 eq.) and $Cu(OTf_2)$ (200 mg, 560 µmol, 0.40 eq.) were dissolved in 7 mL anhydrous dichloromethane and then stirred at 40 °C overnight. The resulting reaction mixture was cooled to room temperature, filtered through a plug of silica and the solvent was removed under reduced pressure. After flash column chromatography (SiO_2; petroleum ether) the product **5** was isolated as a colorless solid (400 mg, 1.13 mmol, 81%).

R_f = 0.68 (SiO_2; petroleum ether/ethyl acetate 9:1).

^{1}H NMR (DMSO-d_6, 500 MHz, rt): δ = 8.95 (d, *J*=8.23 Hz, 2H), 7.70 (t, *J*= 7.69 Hz, 2H), 7.53 (t, *J*=7.76, 2H), 7.30-7.35 (m, 4H), 7.21-7.26 (m, 6H), 6.82 (s, 2H) ppm.

^{13}C {^{1}H} NMR (DMSO-d_6, 126 MHz, rt): δ = 138.8, 138.2, 133.2, 131.0, 130.7, 130.0, 128.1, 127.4, 127.3, 127.2, 127.1, 126.94, 126.8, 123.3 ppm.

Data in accordance with the literature.[S7]

9,10-Dibromo-9,10-dihydrodibenzo[3,4:7,8]cycloocta[1,2-*l*]phenanthrene (**6**)

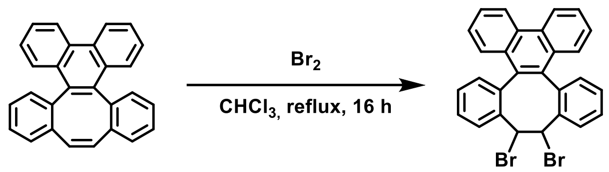

5 **6**

5 (1.45 g, 4.09 mmol, 1.00 eq.) was dissolved in 60 mL chloroform and Br_2 (250 µL, 780 mg, 4.91 mmol, 1.20 eq.) in chloroform (40 mL) was added dropwise at room temperature. The mixture was then subjected to a pre-heated oil bath and stirred at 75 °C overnight. After cooling to room temperature the reaction mixture was washed with $Na_2S_2O_4$ and extracted with dichloromethane. The combined organic layers were dried over $MgSO_4$ and the solvent was removed under reduced pressure. After flash column chromatography (SiO_2; petroleum ether) the product **6** was isolated as a colorless solid (1.53 g, 2.98 mmol, 73%).

R_f = 0.41 (SiO_2; petroleum ether/ethyl acetate 9:1).

^{1}H NMR ($CDCl_3$, 400 MHz, rt): δ = 8.86 (dd, ^4J=8.32 Hz, ^3J=4.65 Hz, 2H), 7.84 (d, J=7.89 Hz, 1H), 7.71-7.76 (m 3H), 7.55-7.60 (m, 3H), 7.32-7.36 (m, 1H), 7.18-7.24 (m, 4H), 7.11-7.17(m, 1H), 7.00-7.02 (m, 1H), 5.44 (dd, ^4J=8.92 Hz, ^3J=18.95 Hz, 2H) ppm.

^{13}C {^{1}H} NMR ($CDCl_3$, 101 MHz, rt): δ = 139.3, 138.9, 137.2, 134.5, 132.6, 129.1, 129.0, 127.6, 127.57, 127.5, 127.4, 126.9, 126.7, 126.7, 122.5, 122.1, 62.5, 58.2 ppm.

Data in accordance with the literature.[S7]

(α, α, β isomer) Dibenzo[*a*,*c*]bisdibenzo[3,4:7,8]phenanthro[9',10':5,6]cycloocta[1,2-*m*:1',2'-*o*]tetraphenylene (**7a**)

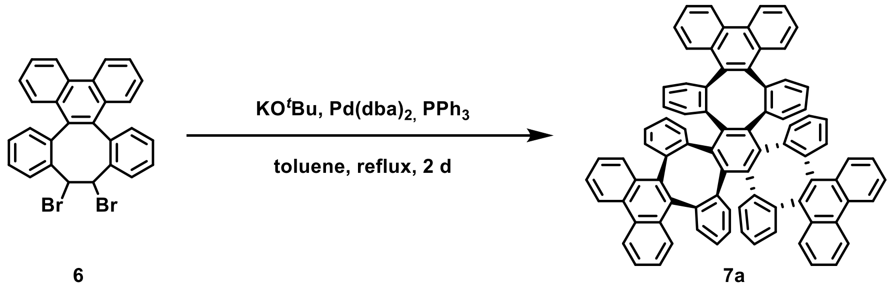

6 **7a**

In a heatgun dried Schlenk tube under an atmosphere of nitrogen (glovebox) **6** (100 mg, 194 µmol, 1.00 eq.) was dissolved in 5 mL anhydrous toluene. Then, $Pd(dba)_2$ (11.0 mg, 20.0 µmol, 10 mol%.) and PPh_3 (2.55 mg, 9.72 µmol, 5 mol%.) were added. Potassium *tert*-butoxide solution (1 mol L^{-1} in tetrahydrofuran, 580 µL, 580 µmol, 3.00 eq.) was rapidly added dropwise and the mixture was stirred at 100 °C over 3 d. After cooling to room temperature the mixture was washed with NH_4Cl and extracted with dichloromethane. The combined organic layers were dried over $MgSO_4$ and the solvent was removed under reduced pressure. After flash column chromatography (SiO_2; petroleum ether/ethyl acetate 9:1 – 6:1) the product **7a** was isolated as a colorless solid (34.0 mg, 32.1 µmol, 50%).

R_f = 0.18 (SiO_2; petroleum ether/ethyl acetate 9:1).

Mp: >300 °C, decomposition from 500 °C.

^{1}H NMR (CD_2Cl_2, 600 MHz, rt): δ = 8.95 (d, *J*=8.44 Hz, 2H), 8.59, (d, *J*=8.32 Hz, 2H), 8.53 (d, *J*=8.50 Hz, 2H), 7.79-7.84 (m, 4H), 7.71 (t, *J*=7.87 Hz, 2H), 7.64 (d, *J*=8.07 Hz, 2H), 7.50-7.57 (m, 6H), 7.39 (d, *J*=8.07 Hz, 2H), 7.35 (d, *J*=7.74 Hz, 2H), 7.24 (t, *J*=7.87 Hz, 2H), 7.04 (d, *J*=7.53 Hz, 2H), 6.89-7.00 (m, 10H), 6.78 (dd, ^4J=7.67 Hz, ^3J=8.95 Hz, 6H), 6.42 (d, *J*=7.60 Hz, 2H), 6.36 (d, *J*=7.74 Hz, 2H) ppm.

^{13}C {^{1}H} NMR (CD_2Cl_2, 151 MHz, rt): δ = 142.6, 142.0, 140.6, 140.5, 140.0, 139.6, 139.5, 139.4, 138.8, 137.51, 137.2, 136.9, 133.4, 132.2, 131.9, 131.4, 130.9, 130.1, 130.0, 129.1, 128.9, 127.9, 126.9, 126.4, 126.4, 126.3, 126.2, 126.0, 125.9, 125.8, 125.6, 124.9,122.7, 122.5, 122.2 ppm.

IR (ATR): $\tilde{\nu}$ = 3059, 3019, 2953, 2922, 2867, 2849, 1489, 1483, 1448, 1438, 1417, 1394 cm^{-1}.

UV-Vis (DCM, rt): $\lambda_{max,\, abs}$ = 356 nm, $\lambda_{max,\, em}$ = 360 nm.

HRMS ($MALDI^+$) m/z: $[M]^{\cdot+}$: calcd. for $[C_{84}H_{48}]^{\cdot+}$: 1056.3756; found 1056.3747; correct isotope distribution.

Crystal data

Brick-shaped colorless single crystals were obtained by slow evaporation of a $CHCl_3$/MeOH solution of **7a**.

Colorless crystal (brick), dimensions 0.102 x 0.052 x 0.029 mm^3, crystal system monoclinic, space group $P2_1/c$, Z=4, *a*=14.5431(7) Å, *b*=15.6779(5) Å, *c*=29.8695(12) Å, *α*=90 °, *β*=29.8695(12) °, *γ*=90 °, *V*=6727.4(5) $Å^3$, *ρ*=1.515 g/cm^3, *T*=200(2) K, Θ_{max}=68.294°, 28574 reflections measured, 7941 unique (R_{int}=0.1522), 4319 observed ($I > 2\sigma(I)$), *μ*=4.93mm^{-1}, T_{min}=0.43, T_{max}=1.00, 901 parameters refined, hydrogen atoms were treated using appropriate riding models, goodness of fit 1.02 for observed reflections, final residual values R1(F)=0.099, wR(F^2)=0.264 for observed reflections, residual electron density -0.55 to 0.87 $eÅ^{-3}$.

(α, α, α isomer) Dibenzo[*a*,*c*]bisdibenzo[3,4:7,8]phenanthro[9',10':5,6]cycloocta[1,2-*m*:1',2'-*o*]tetraphenylene (**7a**)

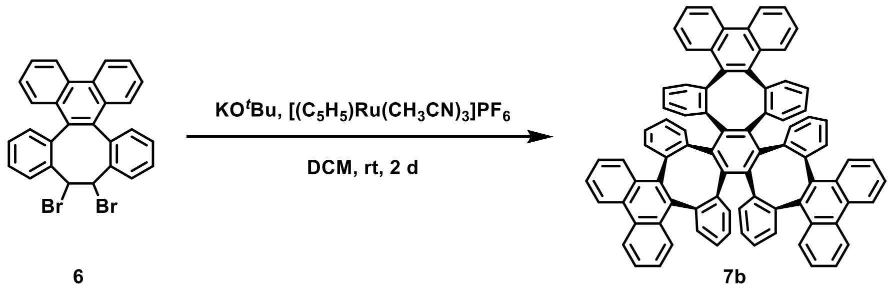

6 **7b**

In a heatgun dried Schlenk tube under an atmosphere of nitrogen (glovebox) **6** (510 mg, 992 µmol, 1.00 eq.) was dissolved in anhydrous 25 mL dichloromethane. Then potassium *tert*-butoxide (334 mg, 2.98 mmol, 3.00 eq.) and $[(C_5H_5)Ru(CH_3CN)_3]PF_6$ (40.0 mg, 100 µmol, 10 mol%.) was added rapidly one after another at room temperature. The mixture was stirred at room temperature for two days. The resulting reaction mixture was filtered through a plug of silica and the solvent was removed under reduced pressure. After flash column chromatography (SiO_2; petroleum ether/ethyl acetate 15:1) the product **7b** was isolated as a pale yellow solid (100 mg, 94.6 µmol, 29%).

R_f = 0.24 (SiO_2; petroleum ether/ethyl acetate 9:1).

Mp: decomposition from 475 °C.

^{1}H NMR (CD_2Cl_2, 500 MHz, rt): δ = 8.34 (d, *J*=8.47 Hz, 6H), 7.48 (d, *J*=7.78 Hz, 6H), 7.34 (t, *J*=7.67 Hz, 6H), 7.08 (t, *J*=7.55 Hz, 6H), 6.95 (t, *J*=7.55 Hz, 6H), 6.82 (t, *J*=7.44 Hz, 6H), 6.51 (d, *J*=7.78 Hz, 6H), 6.40 (d, *J*=8.24 Hz, 6H) ppm.

^{13}C {^{1}H} NMR (CD_2Cl_2, 176 MHz, rt): δ = 141.7, 140.8, 139.00, 137.7, 132.8, 131.6, 131.4, 128.1, 126.6, 126.0, 125.9, 125.6, 122.3 ppm.

IR (ATR): $\tilde{\nu}$ = 2963, 2928, 2359, 2339, 1487, 1449, 1418 cm^{-1}.

UV-Vis (DCM, rt): $\lambda_{max, abs}$ = 358 nm, $\lambda_{max, em}$ = 365 nm.

HRMS ($MALDI^{+}$) m/z: $[M]^{\cdot+}$: calcd. for $[C_{84}H_{48}]^{\cdot+}$: 1056.3756; found 1056.3746; correct isotope distribution.

Crystal data

Plank-shaped, pale yellow single crystals were obtained by slow evaporation of a concentrated THF solution of **7b**.

Pale yellow crystal (plank), dimensions 0.078 x 0.067 x 0.038 mm^3, crystal system monoclinic, space group C_2/c, Z=8, *a*=51.1347(18) Å, *b*=11.0185(2) Å, *c*=25.2123(9) Å, *α*=90 °, *β*=107.776(3) °, *γ*=90 °, *V*=13527.1(8) $Å^3$, *ρ*=1.251g/cm^3, **T**=200(2) K, Θ_{max}= 57.257 °, 43451 reflections measured, 8990 unique (R_{int}=0.0703), 5337 observed (I > 2σ(I)), *μ*=0.57mm^{-1}, $\mathbf{T}_{min}$=0.70, $\mathbf{T}_{max}$=1.45, 915 parameters refined, hydrogen atoms were treated using appropriate riding models, goodness of fit 0.98 for observed reflections, final residual values R1(F)=0.058, wR(F^2)=0.136 for observed reflections, residual electron density -0.28 to 0.26 e$Å^{-3}$.

10,11-Dibromo-10,11-dihydro-5*H*-dibenzo[*a,d*][7]annulen-5-one (**S6**)

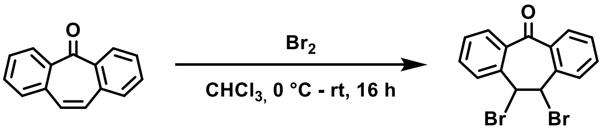

S5 S6

S5 (18.5 g, 89.7 mmol, 1.00 eq.) was dissolved in 150 mL chloroform and cooled with an ice-bath. Then Br_2 (5.18 mL, 16.1 g, 100 mmol, 1.12 eq.) in chloroform (50 mL) was added dropwise and the mixture was stirred at room temperature overnight. The resulting precipitation was filtered and washed several times with chloroform. After drying *in vacuo* the product **S6** was isolated as a colorless solid (26.8 g, 73.3 mmol, 82%).

$\mathbf{R}_f$ = 0.39 (SiO_2; petroleum ether/ethyl acetate 9:1).

^{1}H NMR ($CDCl_3$, 400 MHz, rt): δ = 8.10 (dd, ^{4}J=1.43 Hz, ^{3}J=6.38 Hz, 2H), 7.49-7.59 (m, 4H), 7.42 (dd, ^{4}J=1.42 Hz, ^{3}J=6.40 Hz, 2H), 5.81 (s, 2H) ppm.

^{13}C {^{1}H} NMR ($CDCl_3$, 101 MHz, rt): δ = 192.4, 138.27, 137.0, 133.0, 131.8, 131.3, 130.0, 53.1 ppm.

Data in accordance with the literature.[S8]

10-Bromo-5*H*-dibenzo[*a,d*][7]annulen-5-one (**1**)

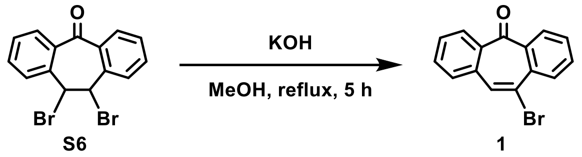

S6 (15.5 g, 42.2 mmol, 1.00 eq.) was added to a solution of potassium hydroxide (7.11 g, 127 mmol, 3.00 eq.) in methanol (160 mL). The mixture was stirred at 70 °C over a period of 5 h. After cooling to room temperature, the mixture was washed with water and extracted with dichloromethane. The combined organic layers were dried over $MgSO_4$ and the solvent was removed under reduced pressure. After drying *in vacuo* the product **1** was isolated as a colorless solid (11.3 g, 39.6 mmol, 94%).

$\mathbf{R_f}$ = 0.63 (SiO_2; petroleum ether/ethyl acetate 4:1).

^{1}H NMR (CD_2Cl_2, 400 MHz, rt): δ = 8.16 (dd, ^4J=0.83 Hz, ^3J=7.29 Hz, 1H), 7.87-7.92 (m, 2H), 7.82 (s, 1H), 7.53-7.69 (m, 4H), 7.47 (d, *J*=7.68 Hz, 1H) ppm.

^{13}C {^{1}H} NMR (CD_2Cl_2, 101 MHz, rt): δ = 195.0, 140.4, 139.9, 135.4, 133.9, 132.7, 133.5, 131.8, 130.9, 130.1, 129.6, 129.4, 126.0 ppm.

Data in accordance with the literature.[S8]

5*H*-Hexabenzo[*c*,*c'*,*c''*,*f*,*f'*,*f''*]benzo[1,2-*a*:3,4-*a'*:5,6-*a''*]tri[7]annulene-5,14,23-trione (**2**)

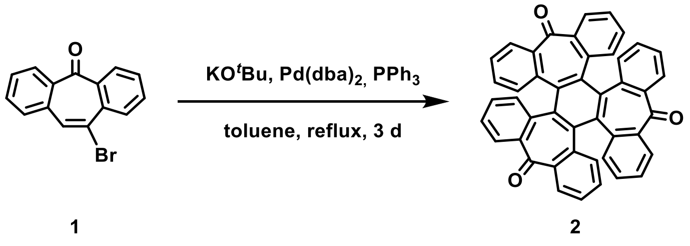

In a heatgun dried Schlenk tube under an atmosphere of argon **1** (10.0 g, 35.1 mmol, 1.00 eq.) was dissolved in 50 mL freshly degassed toluene. Then, $Pd(dba)_2$ (1.01 g, 1.75 mmol, 5 mol%.) and PPh_3 (920 mg, 3.51 mmol, 10 mol%.) were added. Potassium *tert*-butoxide solution (1 mol L^{-1} in tetrahydrofuran, 42.1 mL, 42.1 mmol, 1.20 eq.) was rapidly added dropwise and the mixture was stirred at 100 °C over 3 d. After cooling to room temperature the mixture was washed with NH_4Cl and extracted with dichloromethane. The combined organic layers were dried over $MgSO_4$ and the solvent was removed under reduced pressure. After flash column chromatography (SiO_2; petroleum ether/ethyl acetate 9:1 – 3:2) the product **2** was isolated as a pale yellow solid (3.00 g, 4.90 mmol, 42%).

$\mathbf{R_f}$ = 0.14 (SiO_2; petroleum ether/ethyl acetate 4:1).

Mp: decomposition from 430 °C.

^{1}H NMR ($CDCl_3$, 400 MHz, rt): δ = 7.30 (d, *J*=7.61 Hz, 6H), 7.17 (t, *J*=7.39 Hz, 6H), 6.91-6.97 (m, 12H) ppm.

^{13}C {^{1}H} NMR ($CDCl_3$, 101 MHz, rt): δ = 198.9, 147.5, 137.7, 134.3, 133.0, 129.1, 128.3, 124.6 ppm.

IR (ATR): $\tilde{\nu}$ = 3058, 1707, 1685, 1589, 1286, 1244, 1160 cm^{-1}.

HRMS (DART$^+$) m/z: $[M + NH_4]^{\cdot +}$: calcd. for $[C_{45}H_{28}NO_3]^{\cdot +}$: 630.2064; found 630.2074; correct isotope distribution.

Data in accordance with the literature.[S9]

Note that, at room temperature, **2** adopts a C_3 symmetric conformation according to the resonance pattern in solution. In our hands, **2** crystallized as the (α, α, β) isomer, a conformer differing of the previously reported one.[S9]

(isomeric mixture) 5,14,23-Tris(bromomethylidene)-14,23-dihydro-5*H*-hexabenzo[*c*,*c'*,*c''*,*f*,*f'*,*f''*]benzo[1,2-*a*:3,4-*a'*:5,6-*a''*]tri[7]annulene (**3**)

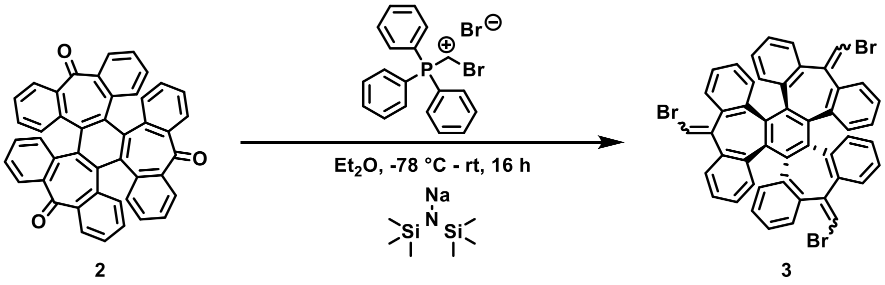

To a stirred suspension of (bromomethyl)triphenylphosphonium bromide (570 mg, 1.31 mmol, 4.00 eq.) in anhydrous diethylether (16 mL) under argon was added sodium bis(hexamethylsilyl)amide (1 mol L^{-1} in tetrahydrofuran, 1.31 mL, 1.31 mmol, 4.00 eq.) at −78 °C. The resulting yellow solution was stirred at −78 °C for 1 h, and then a solution of **2** (200 mg, 330 µmol, 1.00 eq.) in diethylether (4 mL) was added. It was allowed to warm slowly to room temperature and stirred overnight. The reaction mixture was quenched with H_2O and extracted with ethyl acetate. The combined organic layers were dried over $MgSO_4$ and the solvent was removed under reduced pressure. After flash column chromatography (SiO_2; petroleum ether/ethyl acetate 9:1) the product **3** was isolated as a colorless solid and as a mixture of isomers (220 mg, 260 µmol, 80%).

R*f* = 0.50 (SiO_2; petroleum ether/ethyl acetate 4:1).

Mp: decomposition from 375 °C.

^{1}H NMR ($CDCl_3$, 400 MHz, rt): δ = 7.80-7.92 (m, 1H), 7.16-7.27 (m, 4H), 7.00-7.13 (m, 10H), 6.44-6.81 (m, 10H), 6.20-6.33 (1H) ppm.

^{13}C {^{1}H} NMR ($CDCl_3$, 101 MHz, rt): δ = 148.4, 148.1, 147.8, 17.7, 147.4, 147.4, 147.3, 147.2, 147.1, 147.0, 146.2, 138.8, 138.4, 138.3, 137.1, 136.9, 135.8, 135.2, 135.2, 135.1, 135.0, 134.5, 134.1, 134.1, 134.0, 133.1, 132.5, 128.6, 127.9, 127.8, 127.7, 126.4, 126.4, 126.0, 125.9, 125.7, 125.6, 103.9, 103.8, 103.8, 103.7, 103.5, 103.5, 103.3, 103.2, 103.2 ppm.

IR (ATR): $\tilde{\nu}$ = 3055, 3019, 3013, 2955, 2919, 1474, 1438, 1381, 1258, 1092, 1083, 1050, 1033 cm^{-1}.

HRMS (MALDI$^+$) m/z: $[M]^{\cdot +}$: calcd. for $[C_{48}H_{27}Br_3]^{\cdot +}$: 839.9657; found 839.9654; correct isotope distribution.

Crystal data

Plank-shaped, colorless single crystals were obtained by slow evaporation of a concentrated CH_2Cl_2 solution of **3**.

Colorless crystal (plank), dimensions 0.176 x 0.154 x 0.045 mm3, crystal system triclinic, space group P $\bar{1}$, Z=2, *a*=10.5054(5) Å, *b*=13.7386(7) Å, *c*=15.4406(8) Å, *α*=108.6828(13) °, *β*=103.9446(13) °, *γ*=92.9724(13) °, *V*=2028.60(18) Å^3, *ρ*=1.576 g/cm^3, *T*=200(2) K, Θ_{max}= 24.107 °, 24112 reflections measured, 6452 unique (R_{int}=0.0330), 4289 observed (I > 2σ(I)), *μ*=03.22mm^{-1}, T_{min}=0.77, T_{max}=0.90, 562 parameters refined, hydrogen atoms were treated using appropriate riding models, goodness of fit 1.05 for observed reflections, final residual values R1(F)=0.085, wR(F^2)=0.212 for observed reflections, residual electron density -1.23 to 1.13 eÅ^{-3}. Crystals are disordered as a consequence of the cocrystallization of more than one isomer.

(isomeric mixture) 5,14,23-Tris[([1,1'-biphenyl]-2-yl)methylidene]-14,23-dihydro-5*H*-hexabenzo[*c*,*c'*,*c''*,*f*,*f'*,*f''*]benzo[1,2-*a*:3,4-*a'*:5,6-*a''*]tri[7]annulene (**4**)

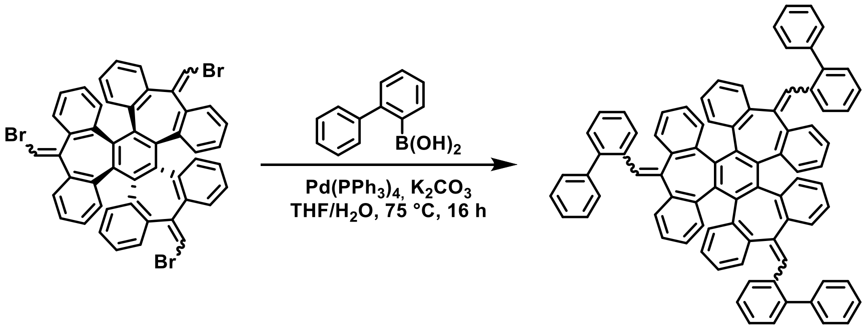

3 **4**

In a heatgun dried Schlenk tube under an atmosphere of argon a mixture of **3** (40.0 mg, 50.0 µmol, 1.00 eq.) and biphenylboronic acid (140 mg, 710 µmol, 15.0 eq.) was dissolved in 8 mL freshly degassed tetrahydrofuran/water (7:1). Then, $Pd(PPh_3)_4$ (10.0 mg, 10.0 µmol, 20 mol%.) and K_2CO_3 (100 mg, 710 µmol, 15.0 eq.) were added, and the reaction mixture was stirred at 75 °C overnight. The mixture was cooled to room temperature, filtered through a plug of silica and the solvent was removed under reduced pressure. After flash column chromatography (SiO_2; petroleum ether/dichloromethane 4:1) the product **4** was isolated as a colorless solid and as mixture of isomers (30.0 mg, 30.0 µmol, 64%).

R_f = 0.35 (SiO_2; petroleum ether/ethyl acetate 4:1).

Mp: decomposition from 450 °C.

IR (ATR): $\tilde{\nu}$ = 3054.21, 3020, 2961, 2921, 2852, 1477, 1434, 1379, 1261, 1094, 1073, 1033, 1008 cm^{-1}.

HRMS (MALDI$^+$) m/z: [M]$^{\cdot+}$: calcd. for $[C_{84}H_{54}]^{\cdot+}$: 1062.4220; found 1062.4207; correct isotope distribution.

The measured ^{1}H- and ^{13}C-NMR spectra are complicated due to the presence of many isomers (see Figure S34/S35).

(α, α, β isomer) Dibenzo[*a*,*c*]bisdibenzo[3,4:7,8]phenanthro[9',10':5,6]cycloocta[1,2-*m*:1',2'-*o*]tetraphenylene (**7a**)

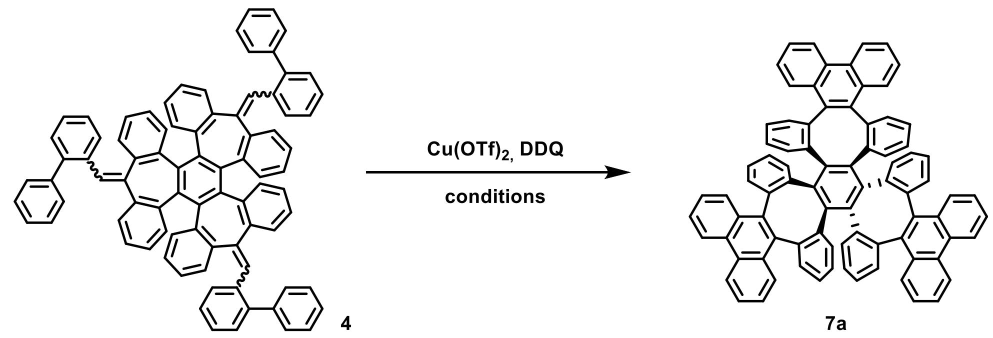

In a heatgun dried microwave glass vial (G10) under an atmosphere of argon **4** (mixture of isomers) (5.00 mg, 4.70 µmol, 1.00 eq.), DDQ and $Cu(OTf_2)$ were dissolved in 2 mL anhydrous solvent. The resulting reaction mixture was cooled to room temperature, filtered through a plug of silica and the solvent was removed under reduced pressure. The conditions are listed in Table S1. Only entry 7, 10 and 11 afforded traces of **7a** based on mass spectrometry and thin layer chromatography.

Table S1: Screening of reaction conditions for ring expansion.

entry	equiv. DDQ, $Cu(OTf)_2$	conditions	result
**1**	5.00, 1.00	dichloromethane, 40 °C, 2 d	**4**
**2**	10.0, 3.00	trichloromethane, 70 °C, 2 d	**4**
**3**	20.0, 10.0	trichloromethane, 70 °C, 7 d	**4**
**4**	20.0, 10.0	dichloroethane, 90 °C, 21 d	**4** + decompostion products[a]
**5**	20.0, 10.0	trichloromethane, 150 °C, microwave, 4 h	**4** + 1-fold ring expansion[a]
**6**	20.0, 10.0	trichloromethane, 150 °C, microwave, 6 h	**4** + 1-and 2-fold ring expansion[a]
**7**	20.0, 10.0	trichloromethane, 150 °C, microwave, 12 h	byproducts + traces of **7a**[a]
**8**	20.0, 10.0	mesitylene, 200 °C, microwave, 4 h	decomposition[a]
**9**	20.0, 10.0	mesitylene, 150 °C, microwave, 48 h	decomposition[a]
**10**	20.0, 10.0	chlorobenzene, 135 °C, microwave, 48 h	byproducts + traces of **7a**[a, b]
**11**	20.0, 10.0	chlorobenzene, 135 °C, microwave, 62 h	byproducts + traces of **7a**[a, b]

[a]Detected by mass spectrometry

[b]Detected by thin layer chromatography

Preliminary Experiments for Li Cation Binding

To demonstrate binding of Li cations in solution we did the following preliminary experiments:

2 mg of **7b** were placed in a NMR-tube under inert conditions, dissolved in anhydrous deuterated dichloromethane and LiOTf or $LiClO_4$ were added in excess. To increase the solubility, the reaction with $LiClO_4$ was performed also in deuterated dimethylsulfoxide. The ^1H NMR spectra failed to show shifts of the signals - even after several hours. This preliminary study leads to the assumption that no spontaneous complex formation/binding was observed under our conditions.

2. Results and Discussion

2.1 Mass Spectrometry

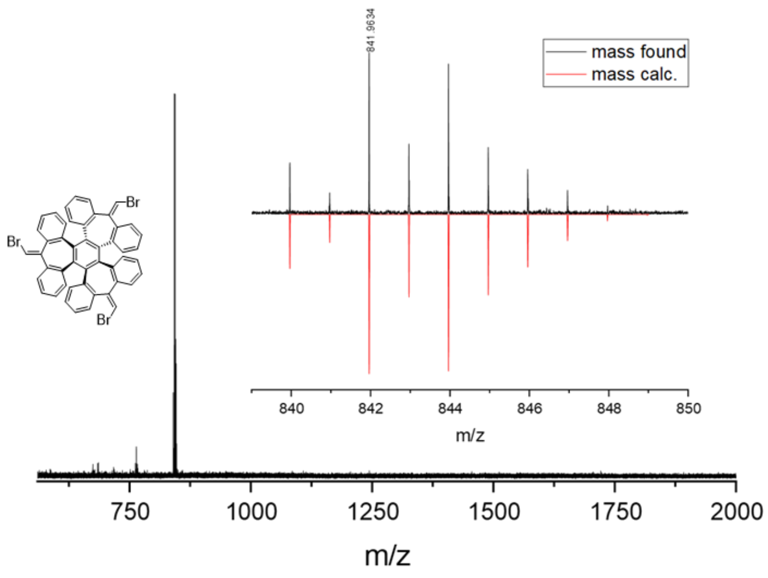

Figure S1: Mass spectrum (HR-MALDI) of **3** (mixture of isomers).

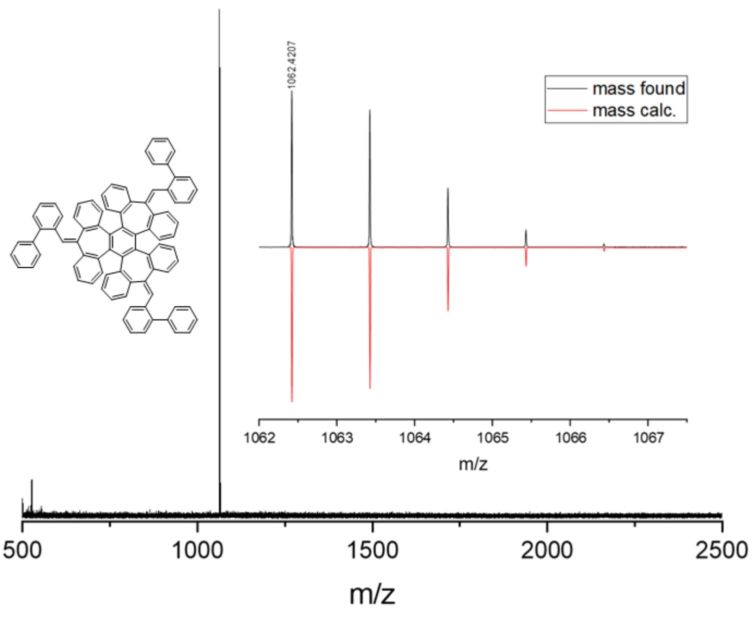

Figure S2: Mass spectrum (HR-MALDI) of **4** (mixture of isomers).

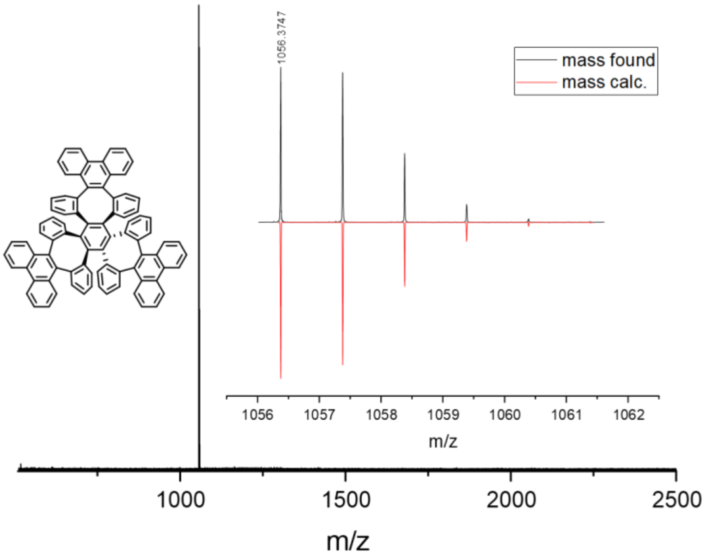

Figure S3: Mass spectrum (HR-MALDI) of **7a**.

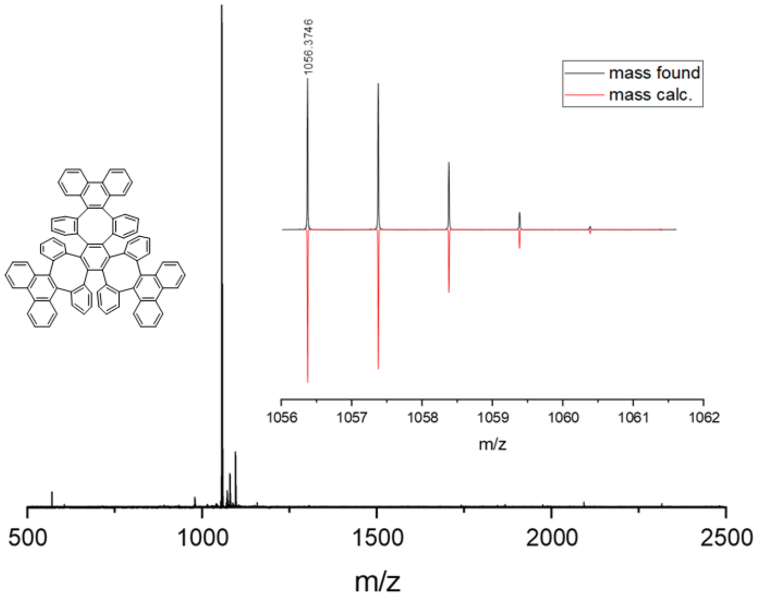

Figure S4: Mass spectrum (HR-MALDI) of **7b**.

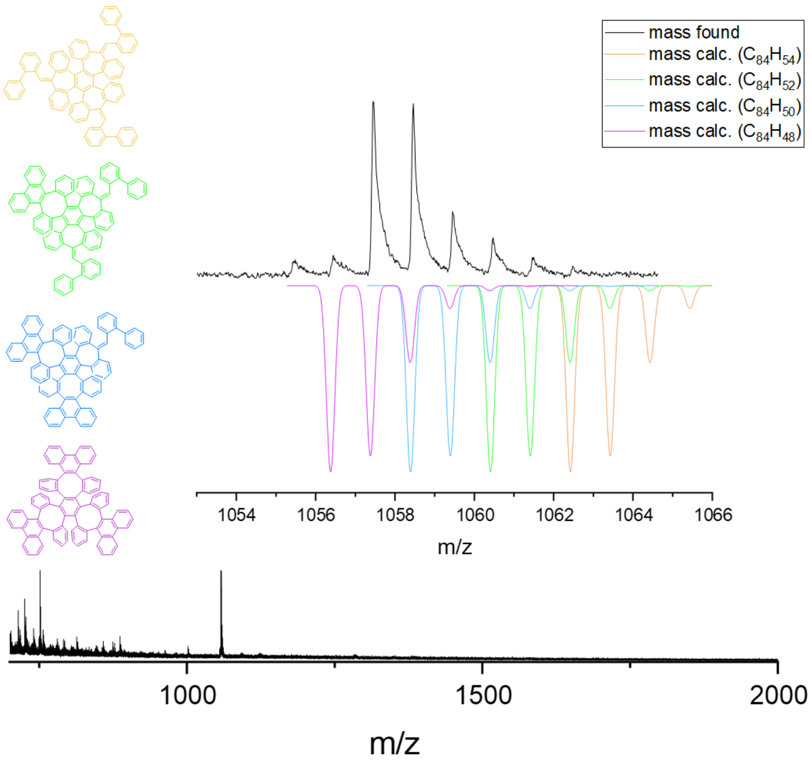

Figure S5: Mass spectrum (MALDI) of the crude product **7a** (from oxidative ring expansion of **4** (mixture of isomers) in chloroform, 150 °C, microwave, 12 h).

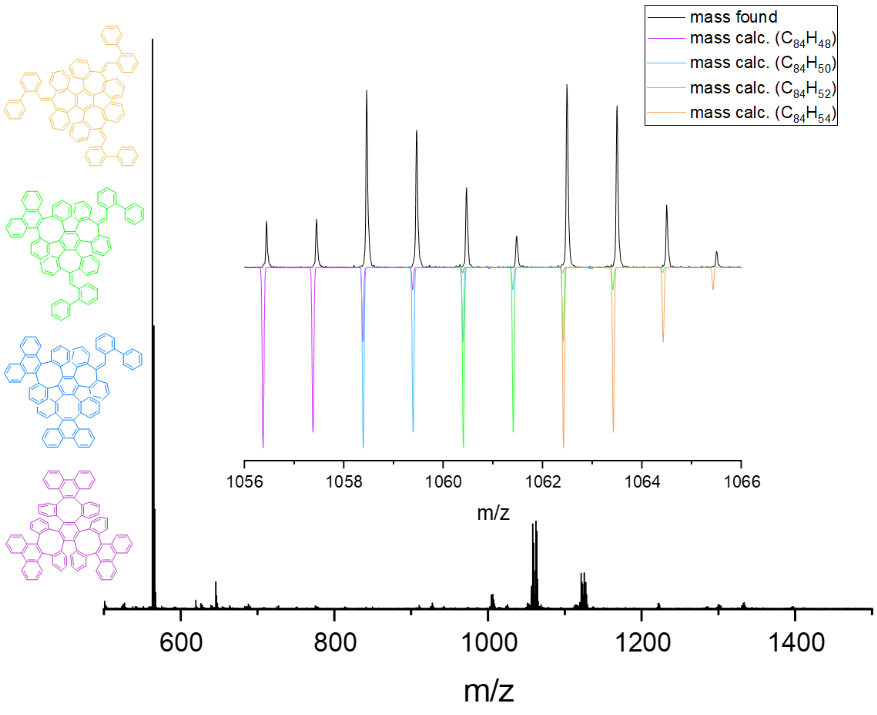

Figure S6: Mass spectrum (MALDI) of the crude product **7a** (from oxidative ring expansion of **4** (mixture of isomers) in chlorobenzene, 135 °C, microwave, 48 h).

2.2 NMR Spectroscopy

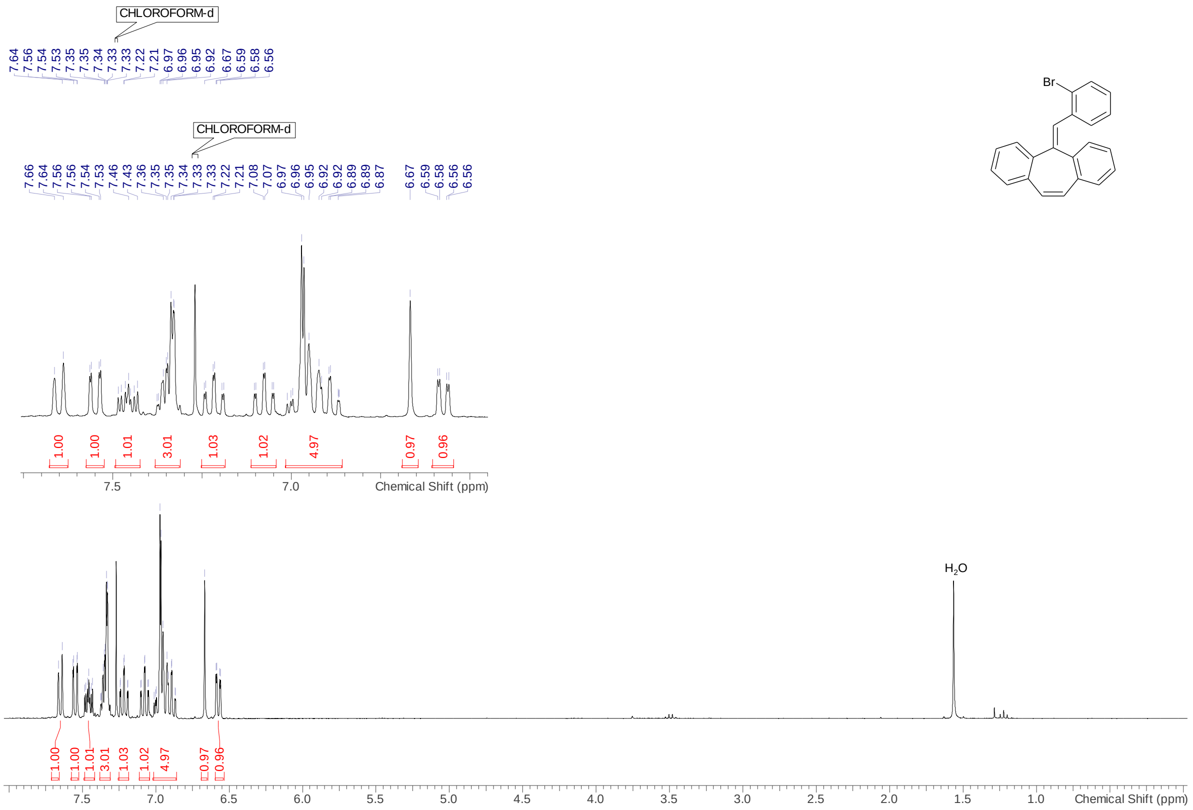

Figure S7: ^{1}H NMR spectrum (300 MHz) of **S3** in CDCl₃.

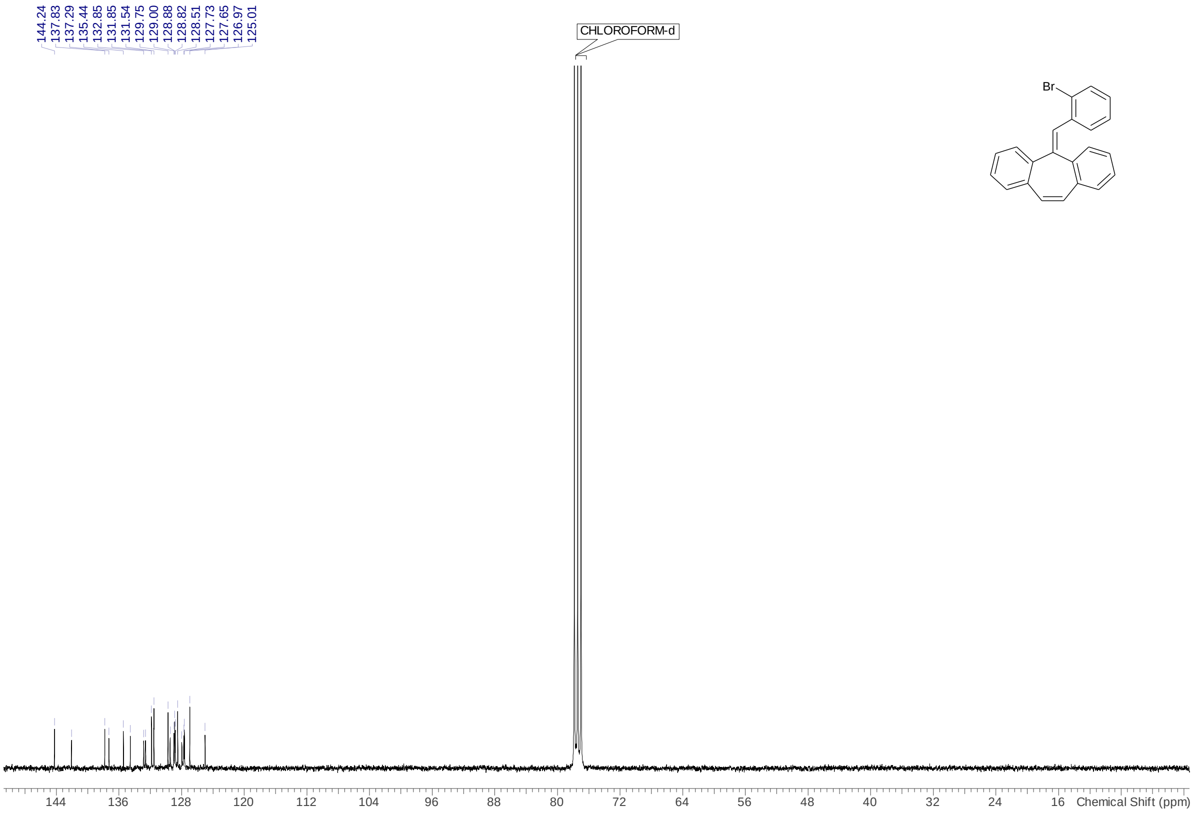

Figure S8: ^{13}C {^{1}H} NMR spectrum (75 MHz) of **S3** in CDCl₃.

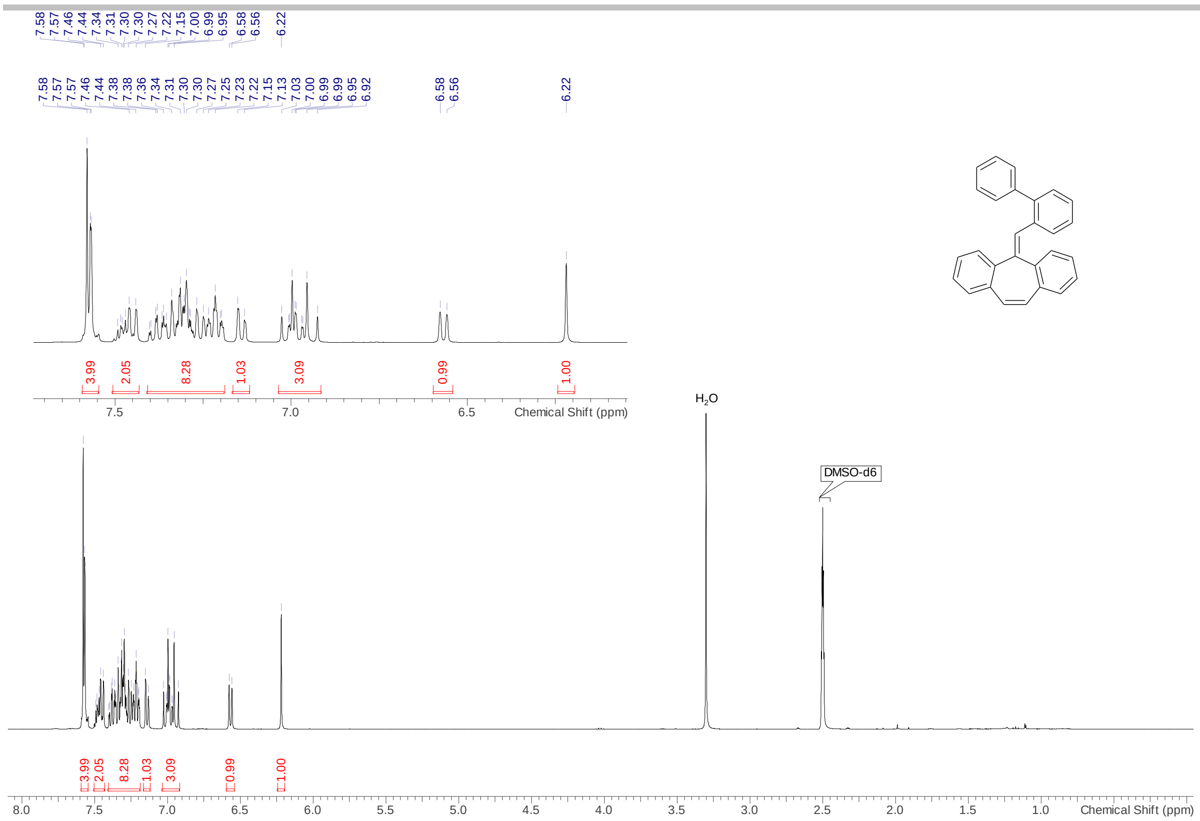

Figure S9: ^{1}H NMR spectrum (400 MHz) of **S4** in DMSO-d_6.

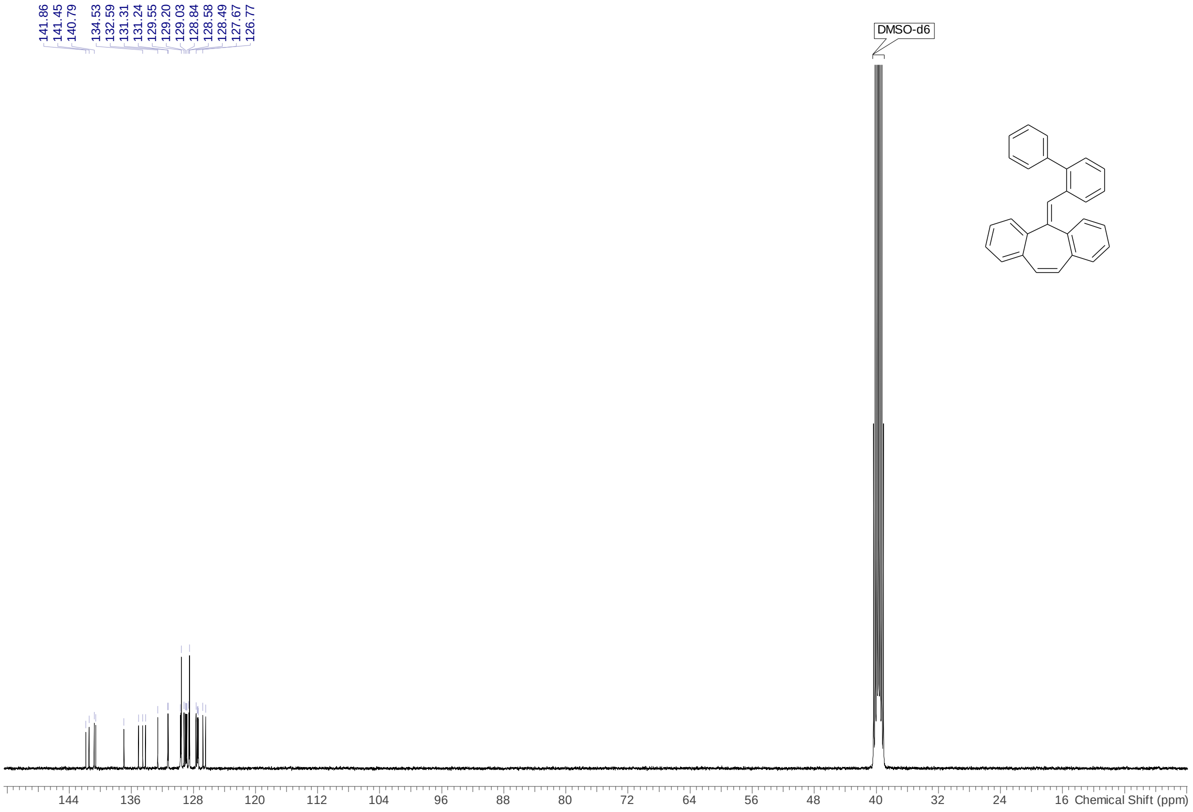

Figure S10: $^{13}C\{^{1}H\}$ NMR spectrum (101 MHz) of **S4** in DMSO-d_6.

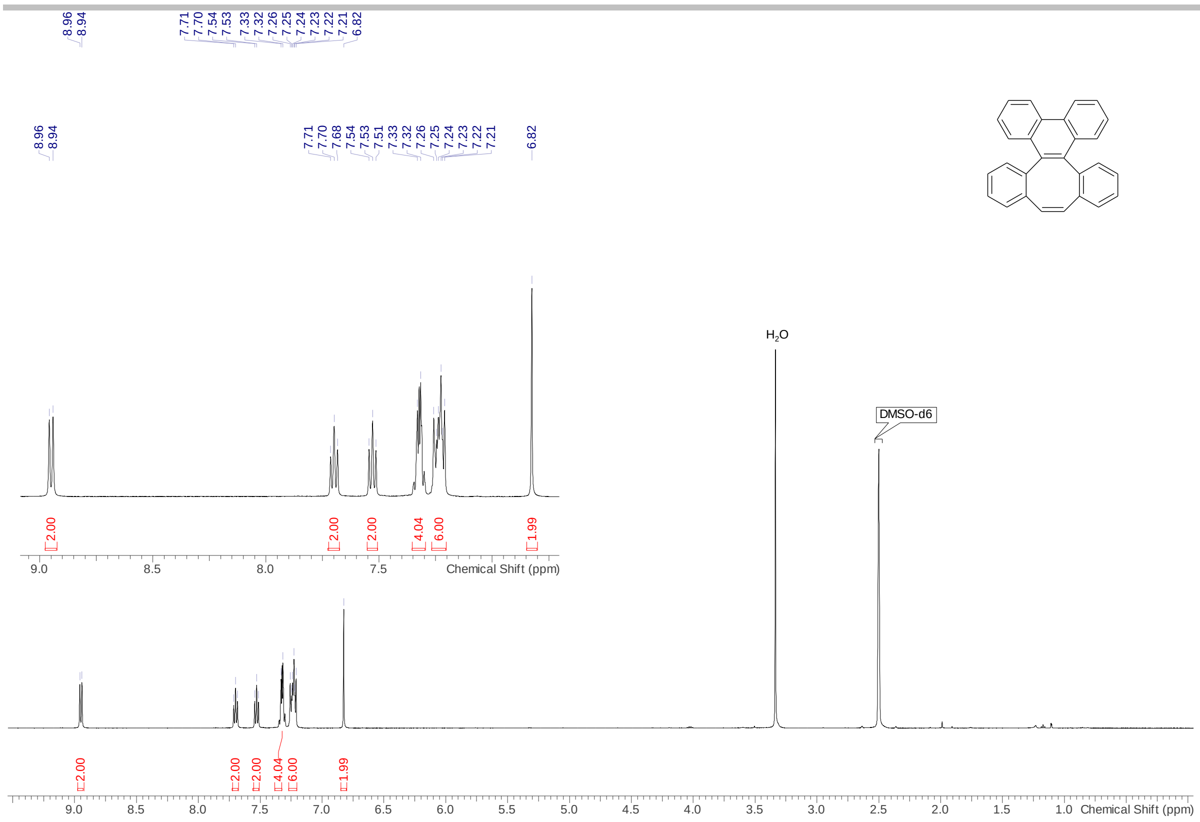

Figure S11: ^{1}H NMR spectrum (500 MHz) of **5** in DMSO-d$_6$.

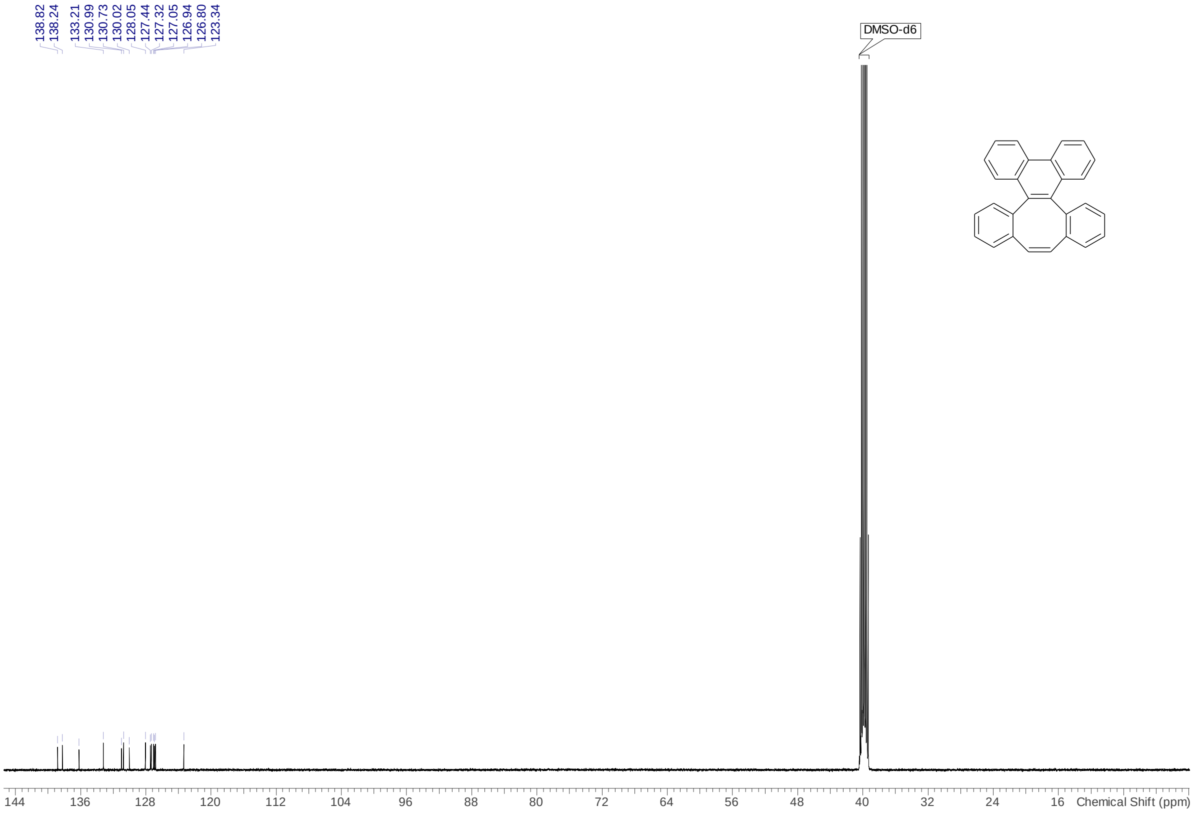

Figure S12: ^{13}C {^{1}H} NMR spectrum (126 MHz) of **5** in DMSO-d$_6$.

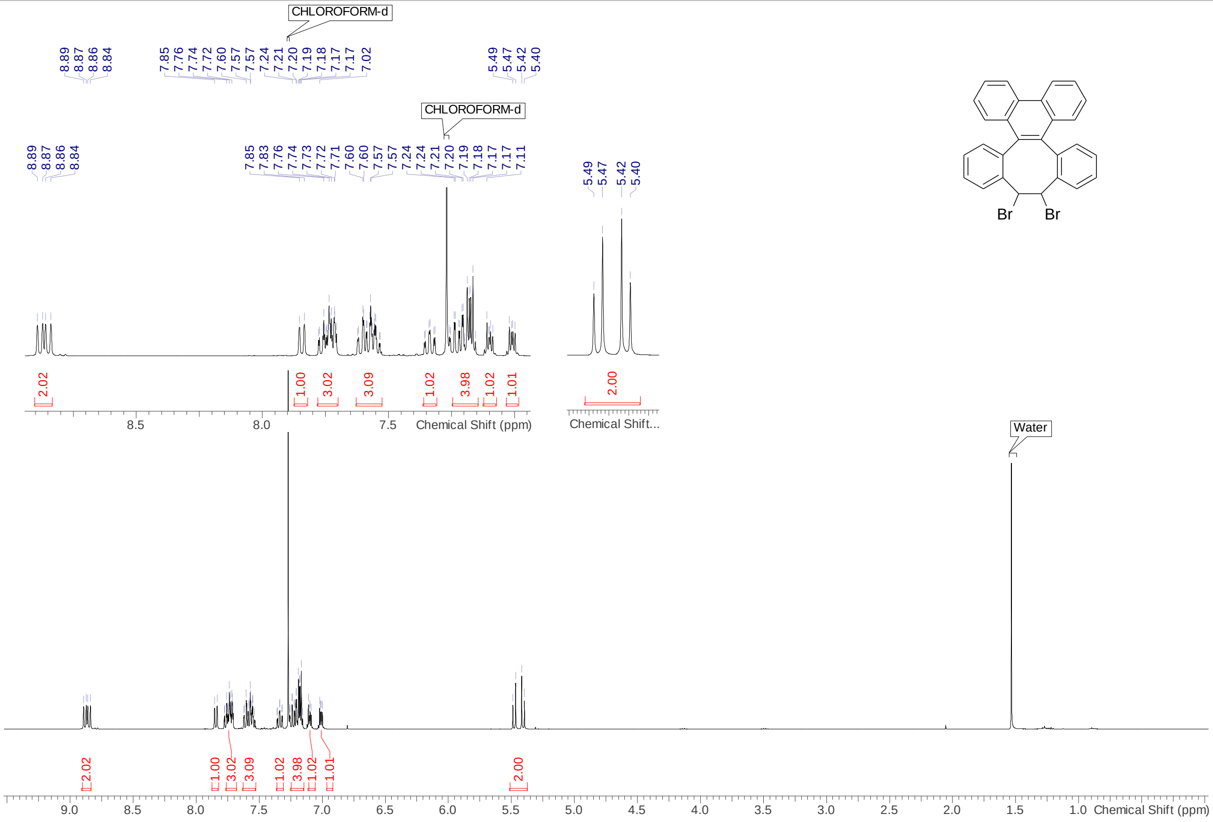

Figure S13: ^{1}H NMR spectrum (400 MHz) of **6** in $CDCl_3$.

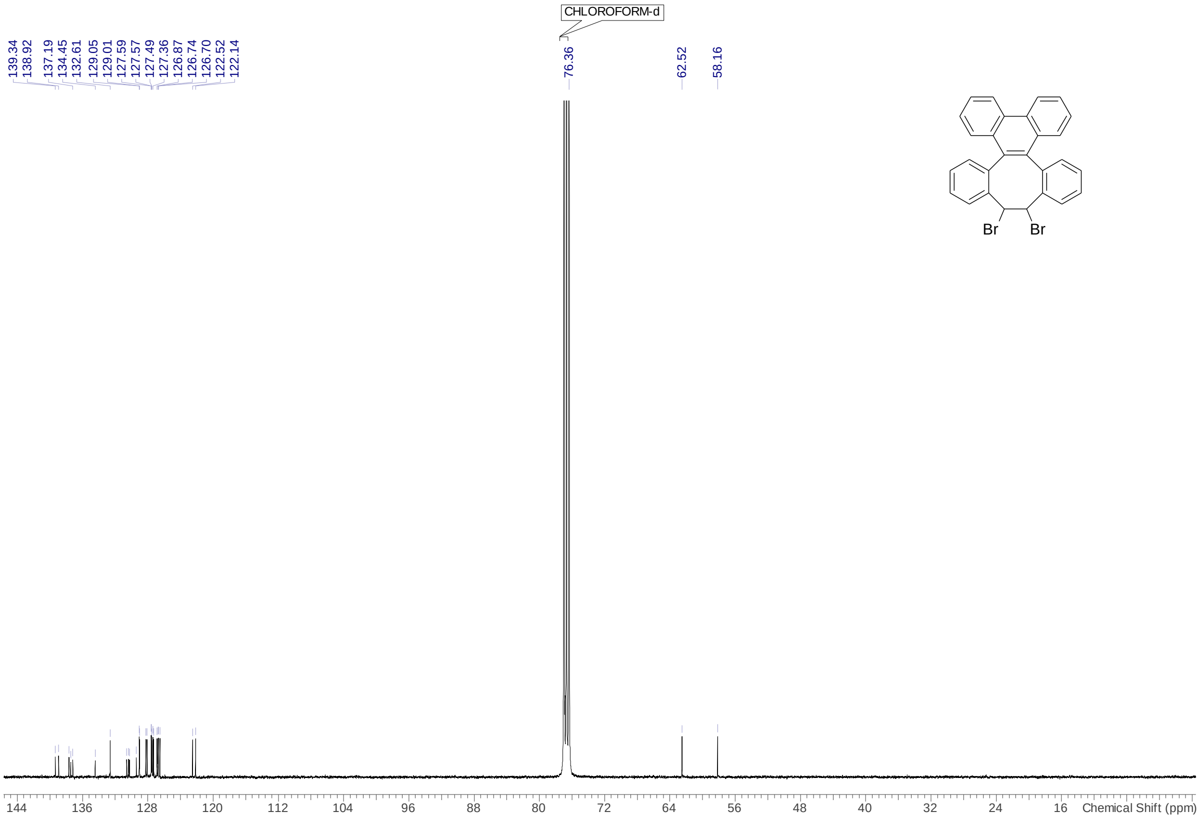

Figure S14: ^{13}C {^{1}H} NMR spectrum (101 MHz) of **6** in $CDCl_3$.

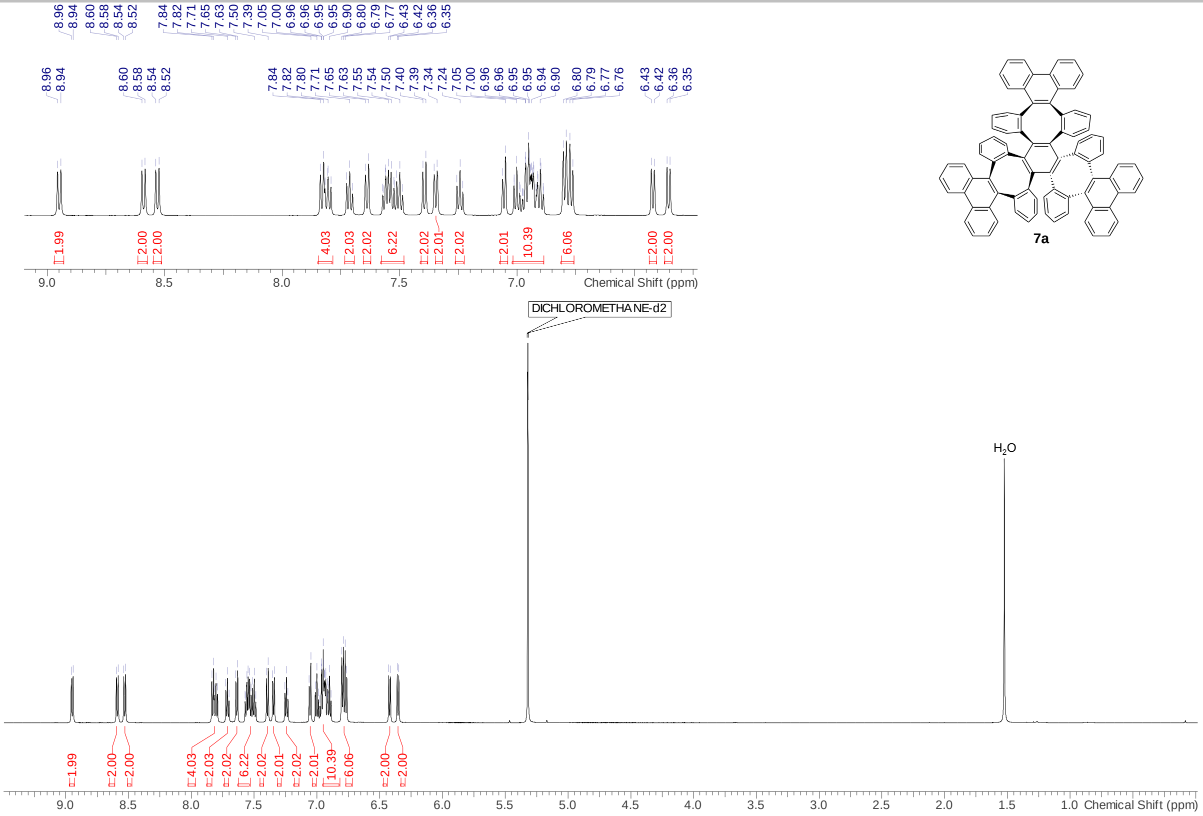

Figure S15: ^{1}H NMR spectrum (600 MHz) of **7a** in CD_2Cl_2.

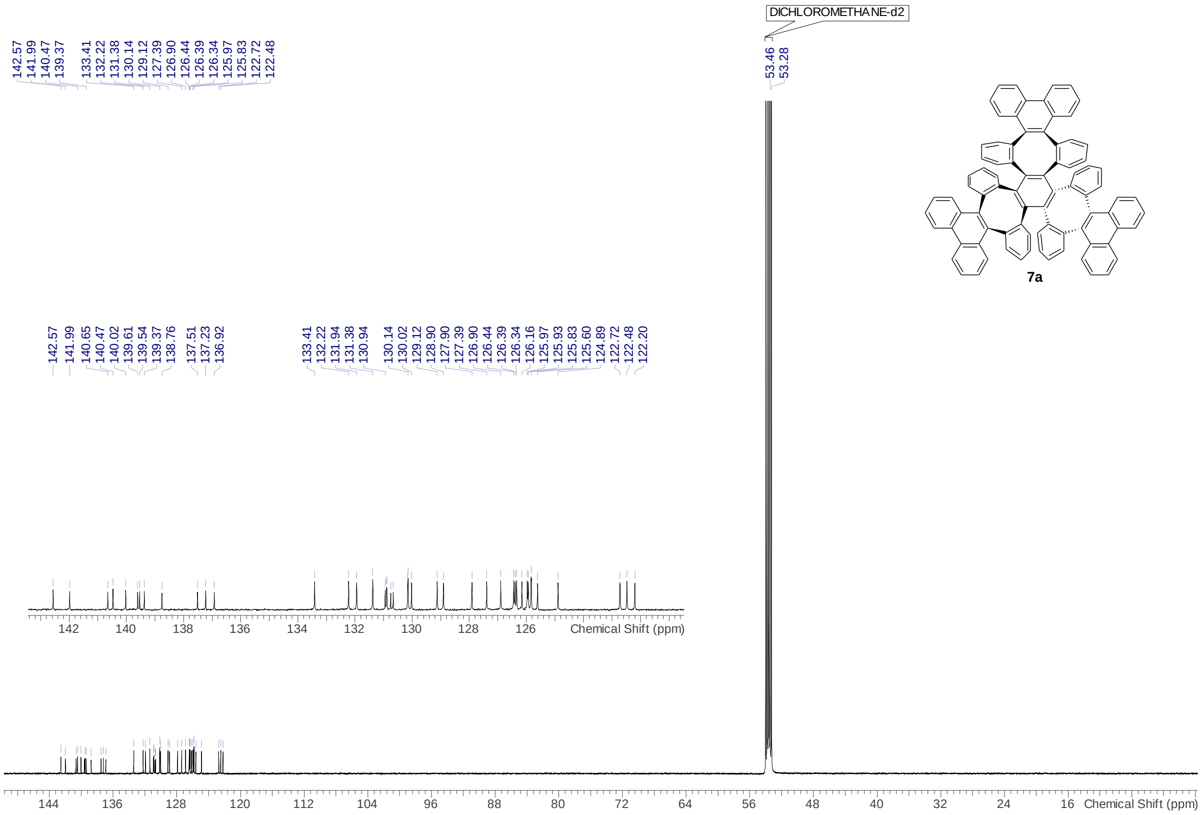

Figure S16: ^{13}C {^{1}H} NMR spectrum (151 MHz) of **7a** in CD_2Cl_2.

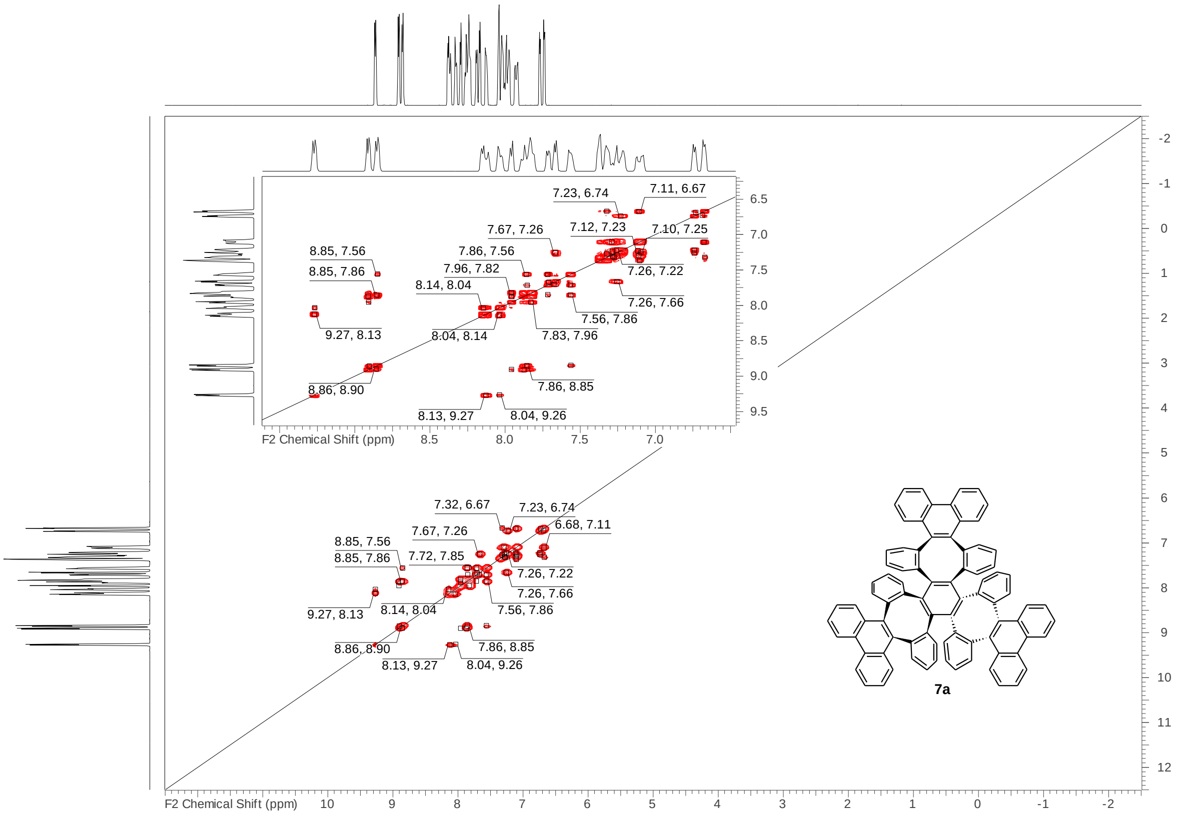

Figure S17: ^{1}H,^{1}H COSY NMR spectrum (600 MHz) of **7a** in CD_2Cl_2.

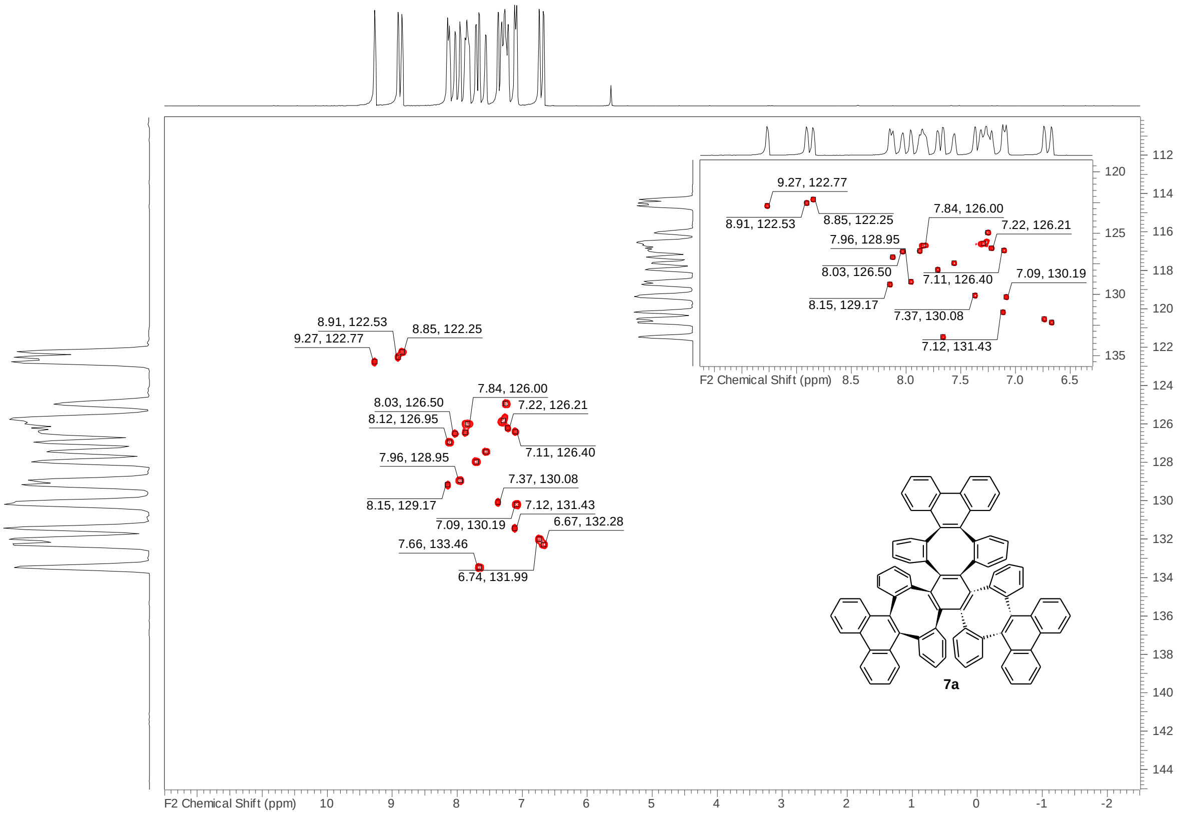

Figure S18: ^{1}H,^{13}C HSQC (me) NMR spectrum (600 MHz/151 MHz) of **7a** in CD_2Cl_2.

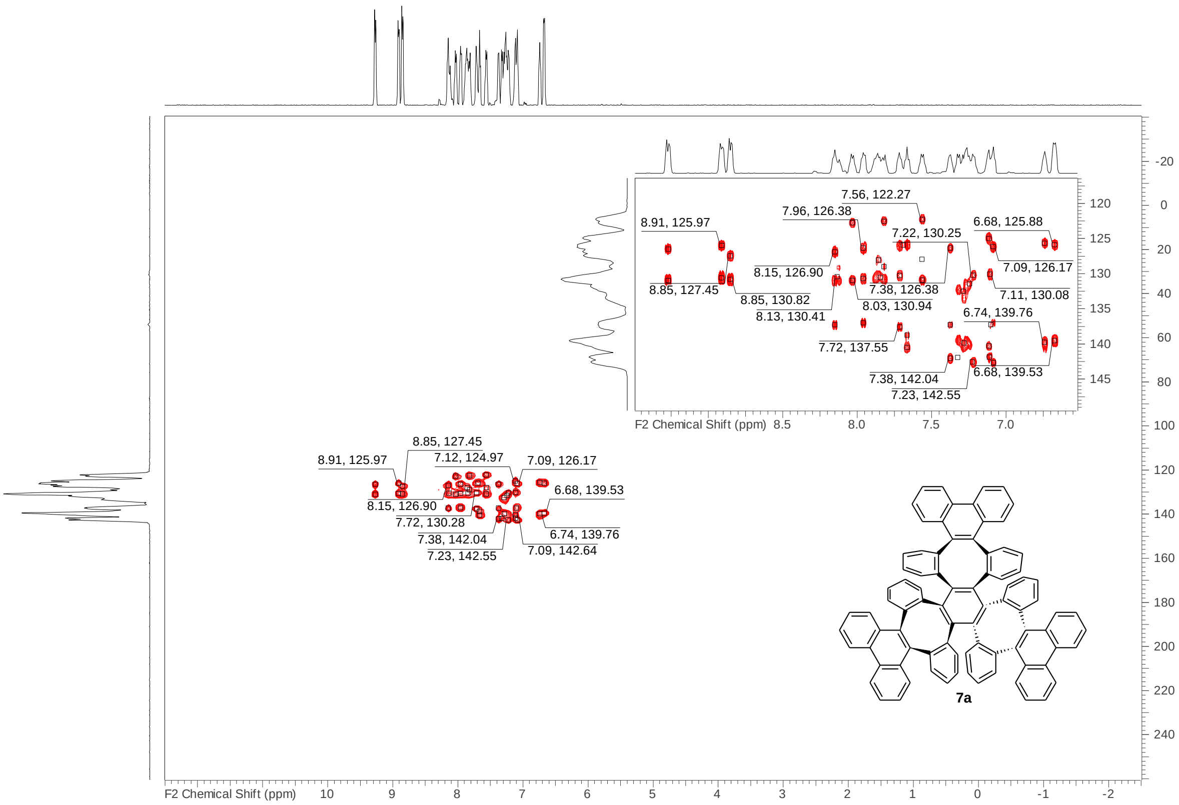

Figure S19: $^{1}H,^{13}C$ HMBC NMR spectrum (600 MHz/151 MHz) of **7a** in CD_2Cl_2.

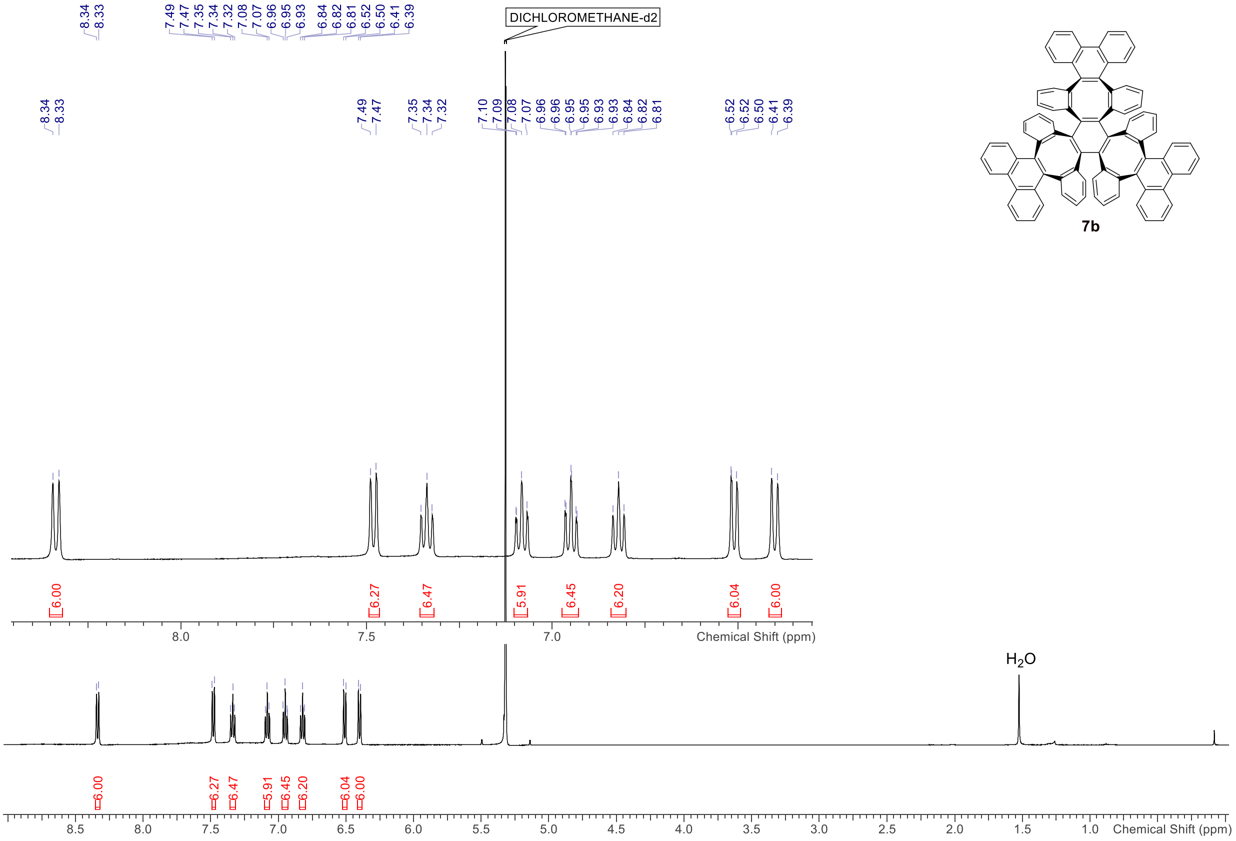

Figure S20: ^{1}H NMR spectrum (500 MHz) of **7b** in CD_2Cl_2.

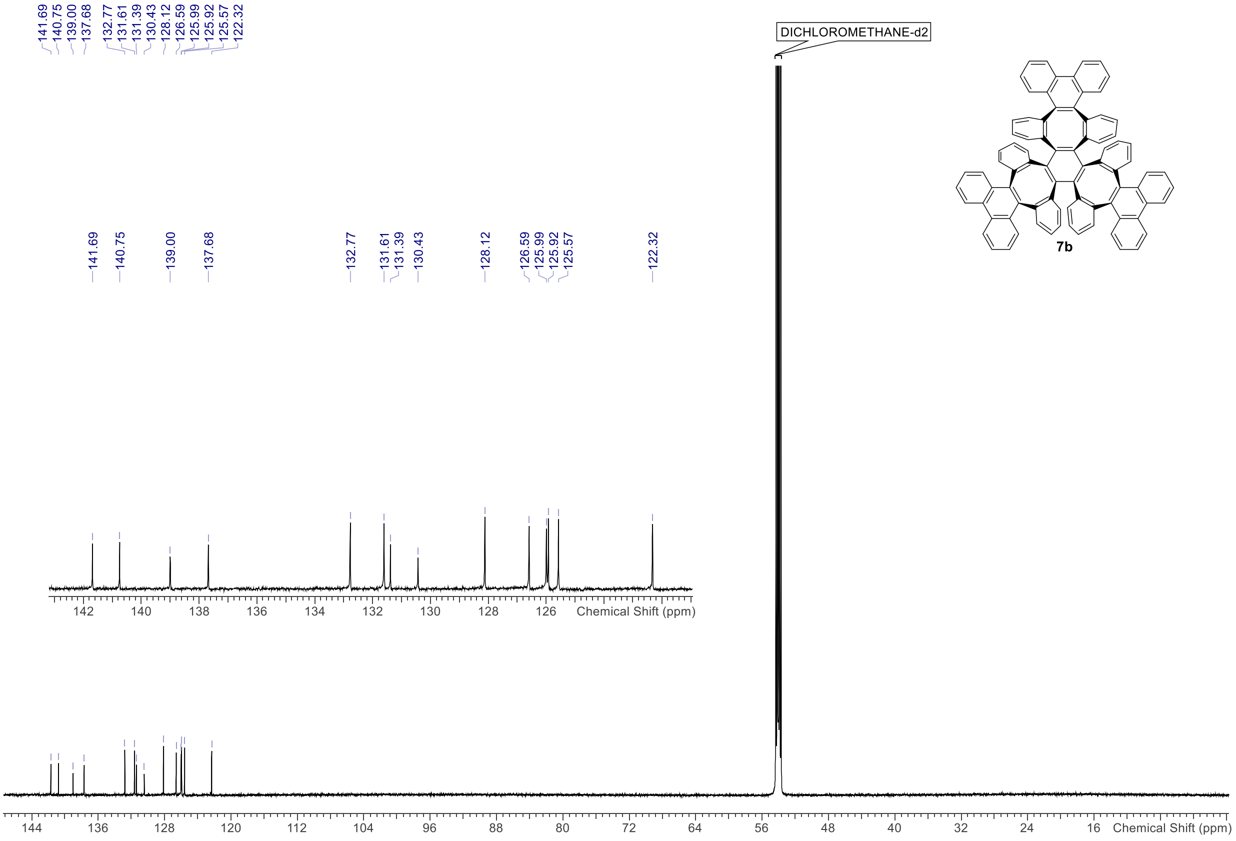

Figure S21: ^{13}C {^{1}H} NMR spectrum (176 MHz) of **7b** in CD_2Cl_2.

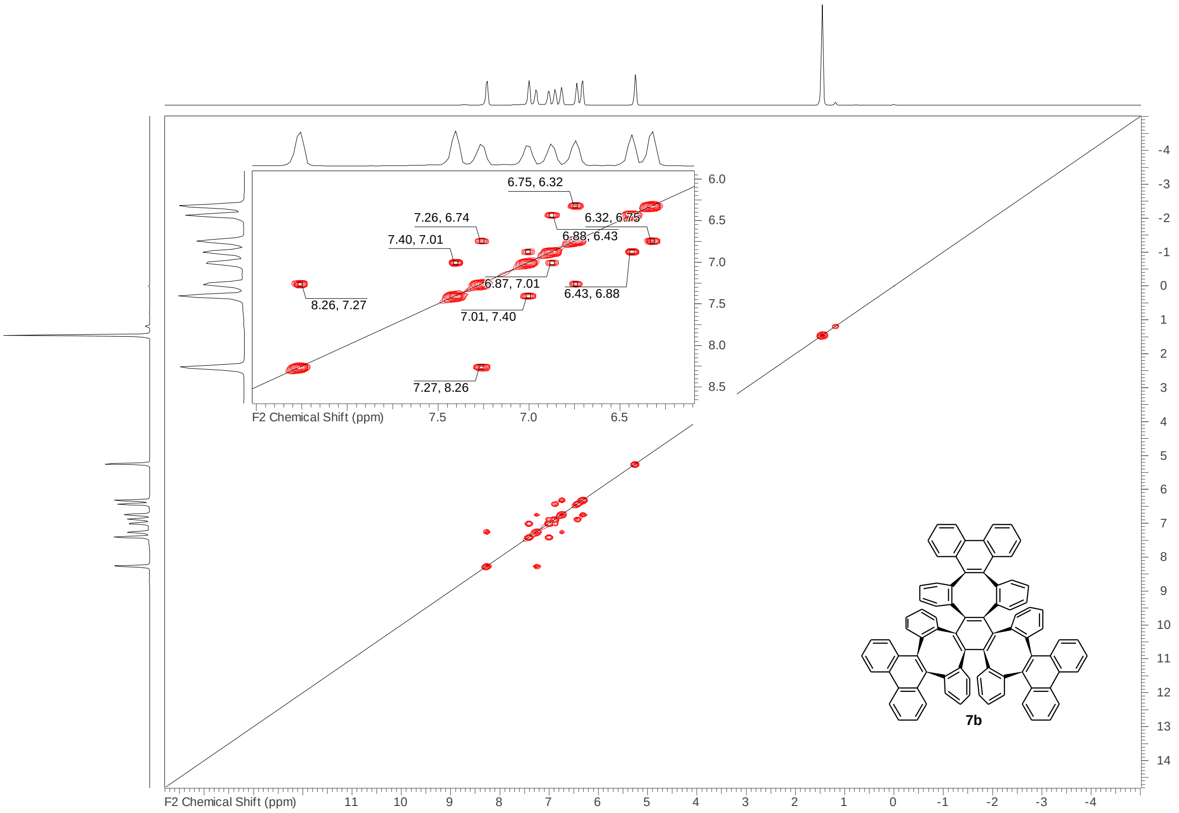

Figure S22: ^{1}H, ^{1}H COSY NMR spectrum (700 MHz) of **7b** in CD_2Cl_2.

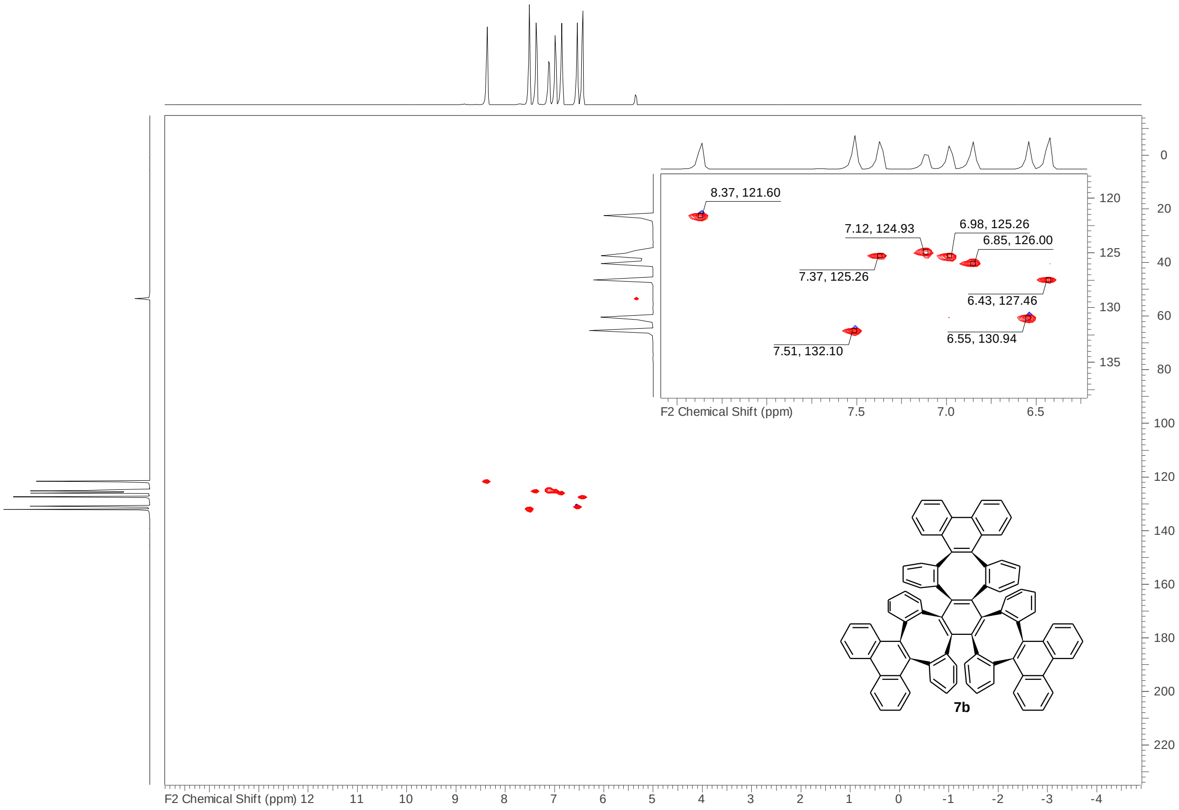

Figure S23: ^{1}H, ^{13}C HSQC (me) NMR spectrum (700 MHz/151 MHz) of **7b** in CD_2Cl_2.

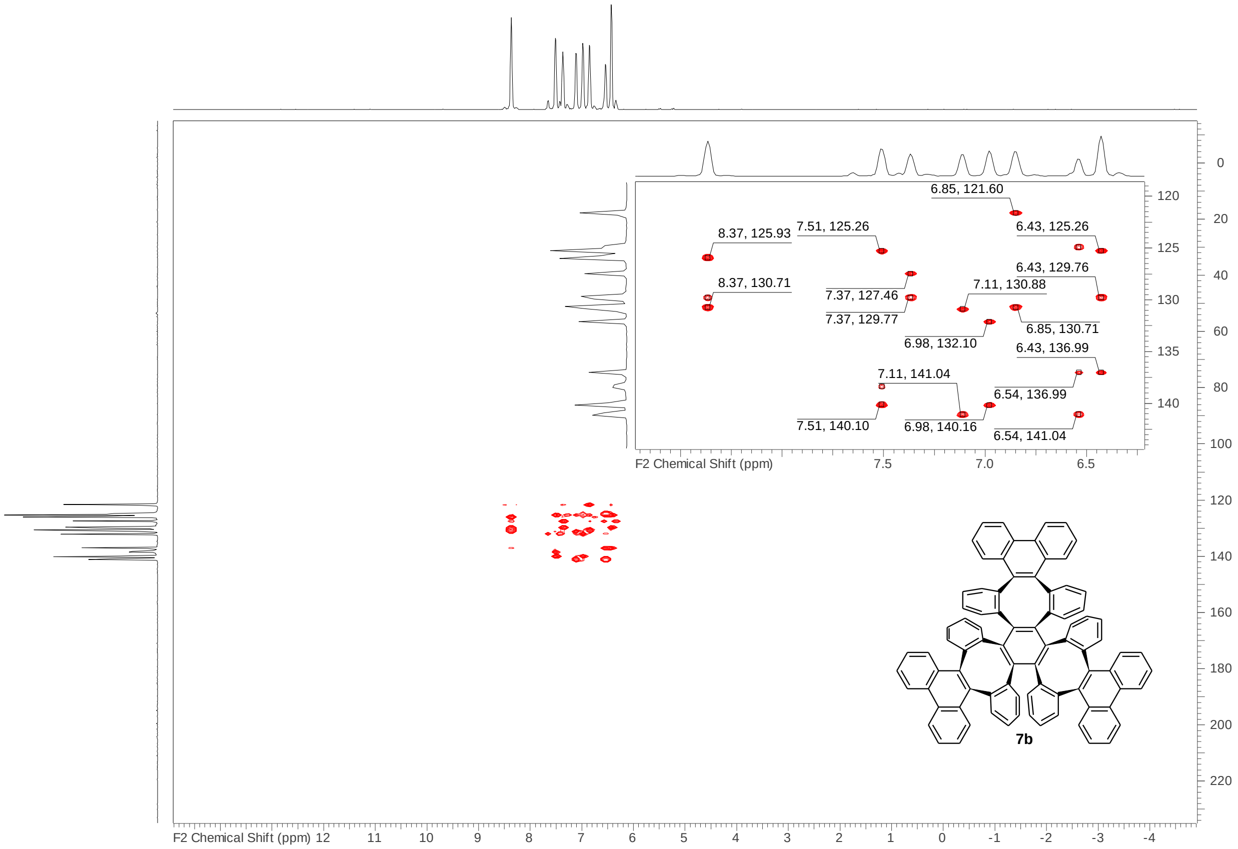

Figure S24: ^{1}H, ^{13}C HMBC NMR spectrum (700 MHz/151 MHz) of **7b** in CD_2Cl_2.

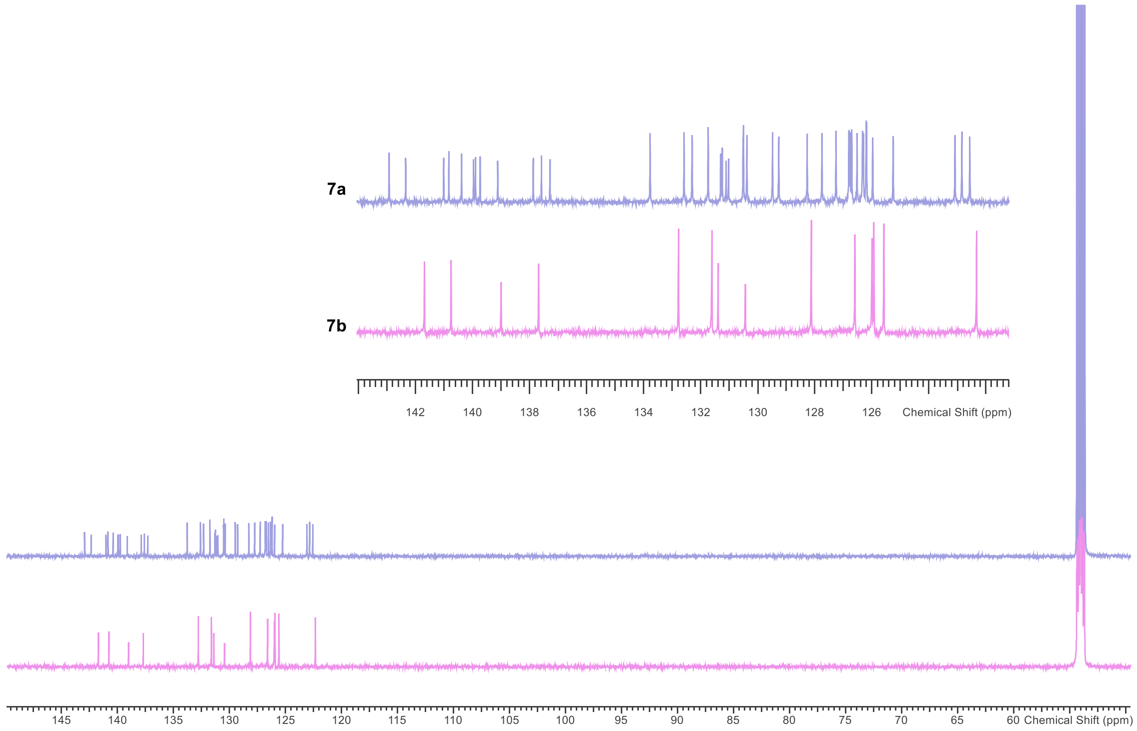

Figure S25: ^{13}C {^{1}H} NMR spectra of **7a** (151 MHz) and **7b** (176 MHz) in CD_2Cl_2.

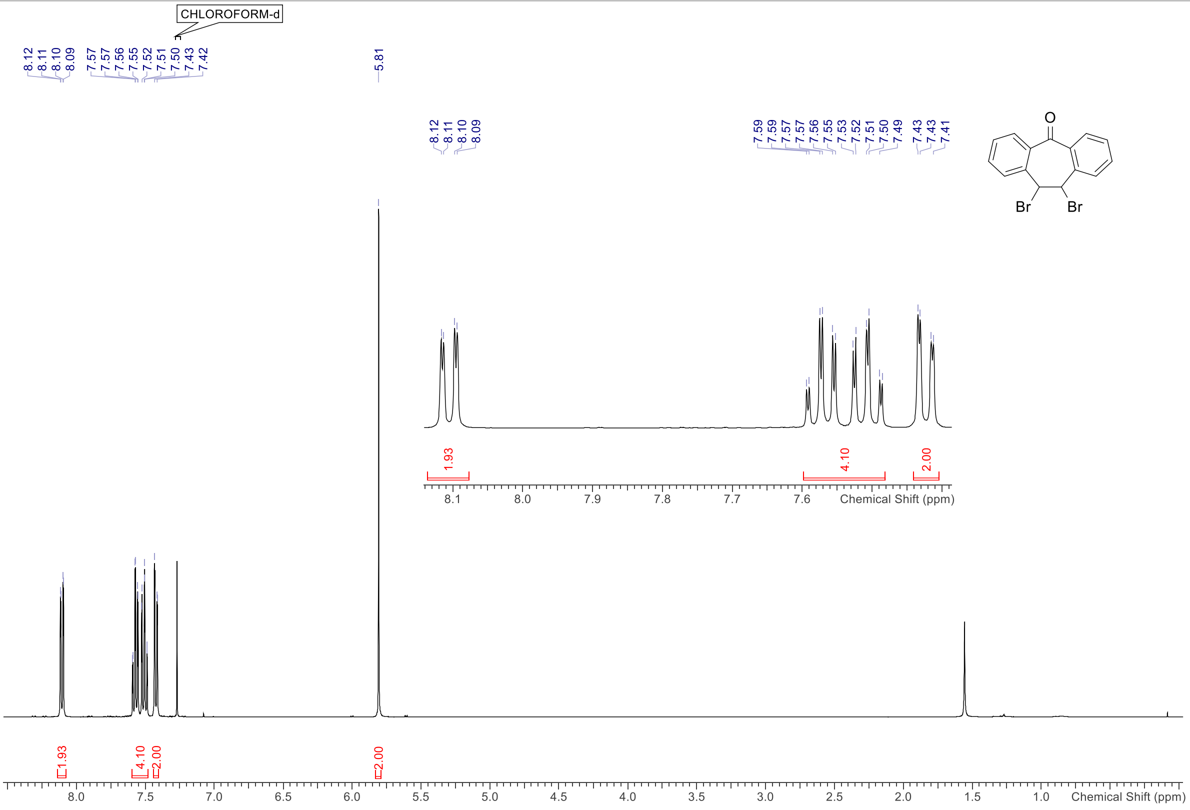

Figure S26: ^{1}H NMR spectrum (400 MHz) of **S5** in $CDCl_3$.

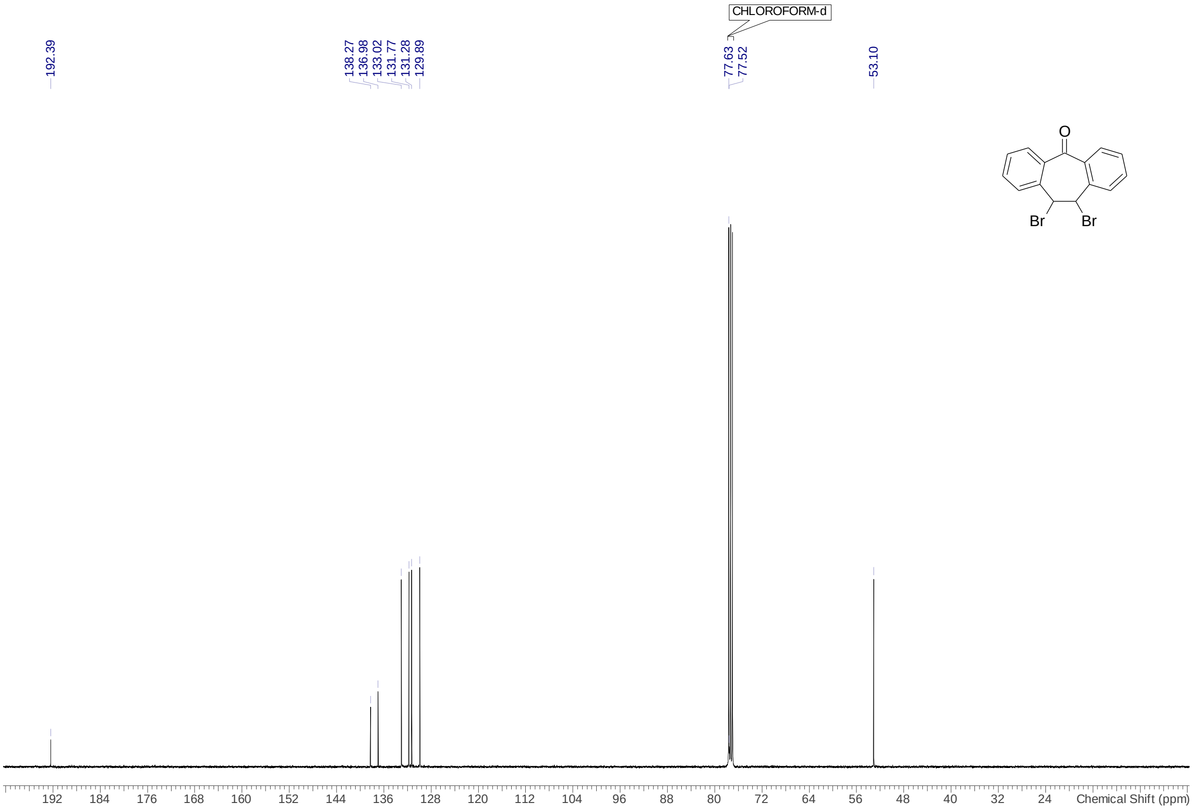

Figure S27: ^{13}C {^{1}H} NMR spectrum (101 MHz) of **S5** in $CDCl_3$.

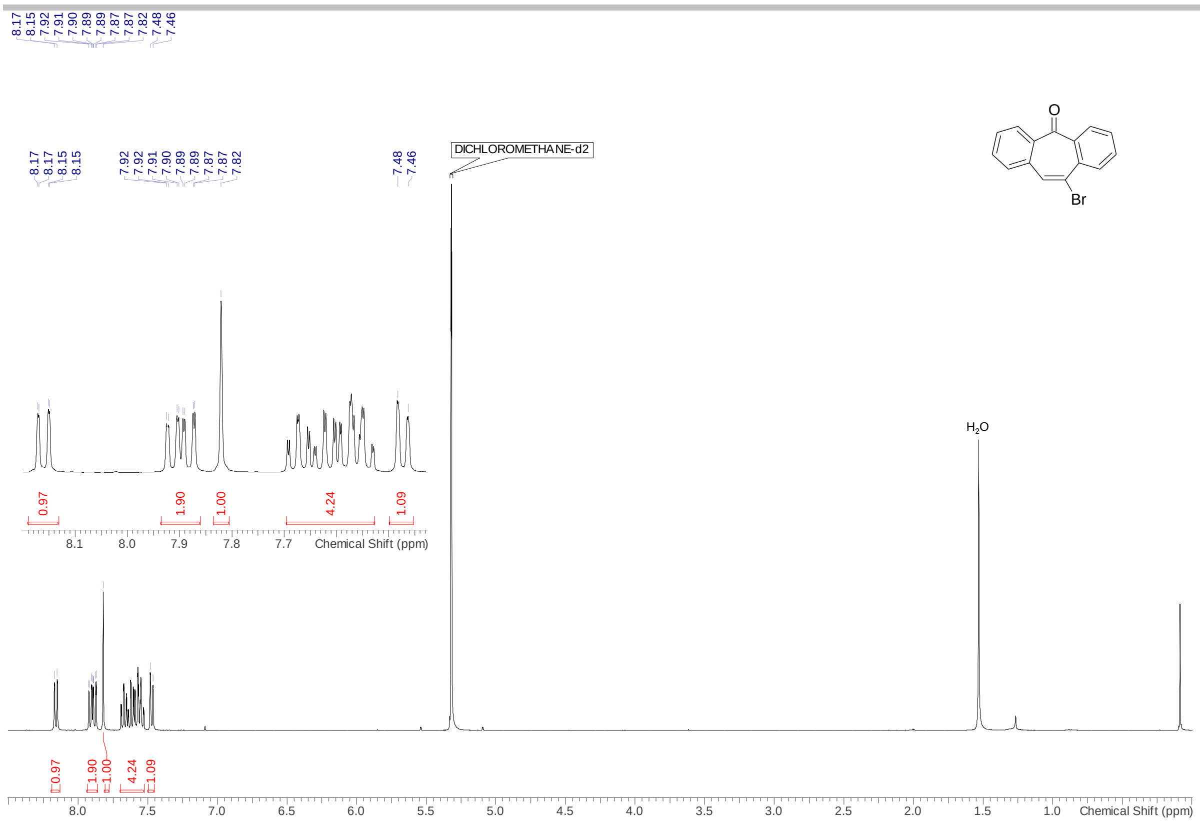

Figure S28: ^{1}H NMR spectrum (400 MHz) of **1** in CD_2Cl_2.

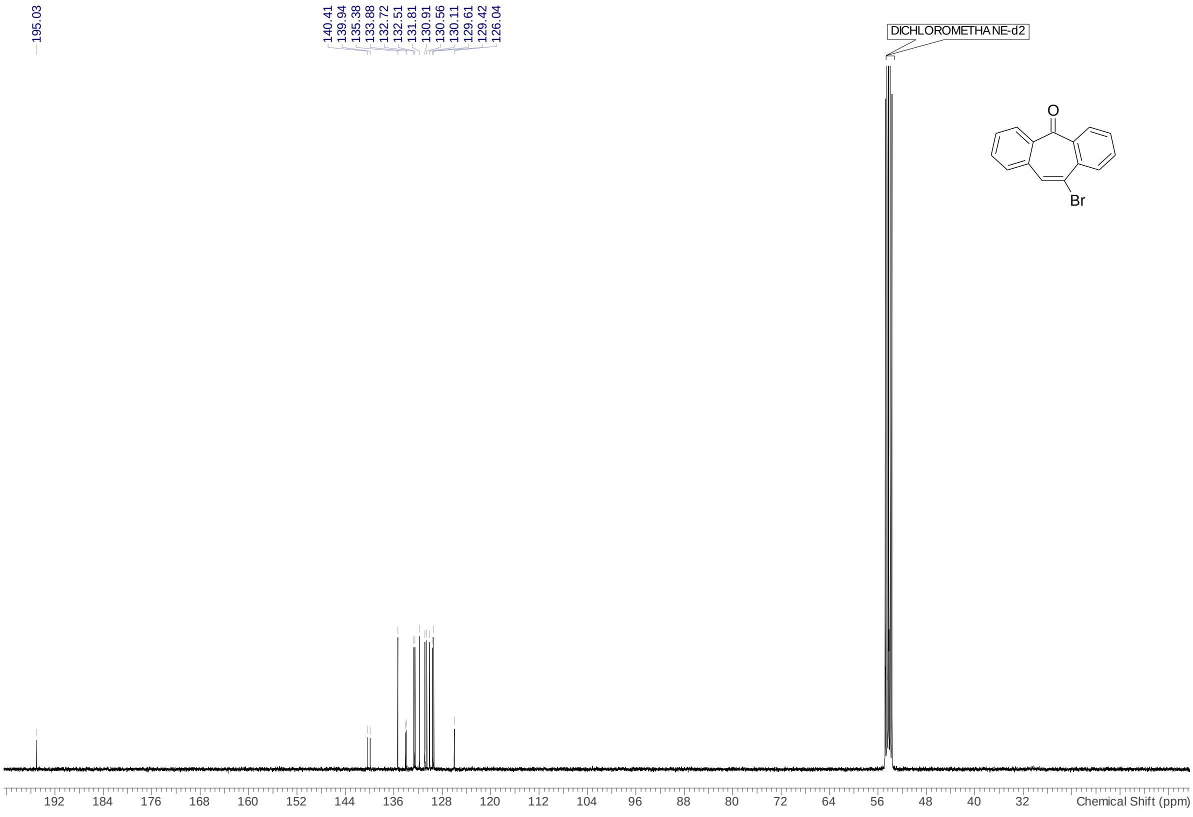

Figure S29: ^{13}C {^{1}H} NMR spectrum (101 MHz) of **1** in CD_2Cl_2.

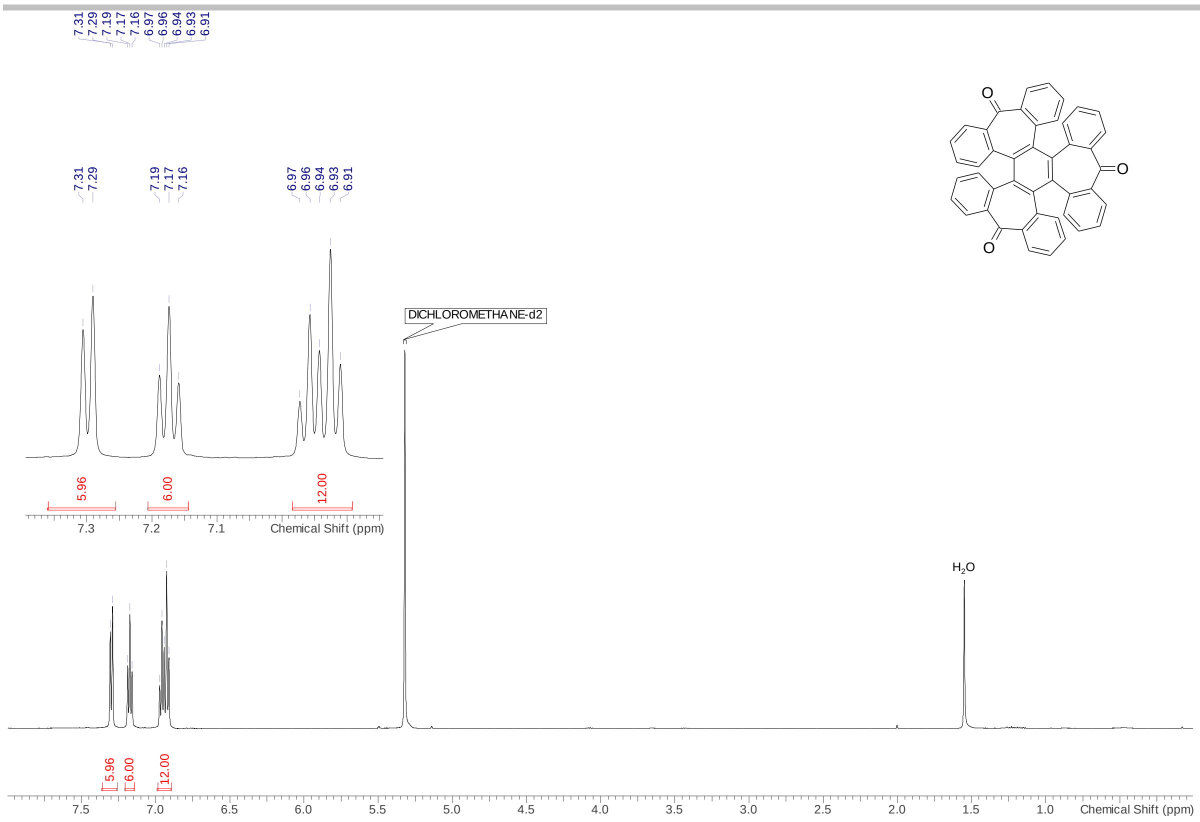

Figure S30: ^{1}H NMR spectrum (500 MHz) of **2** in CD_2Cl_2.

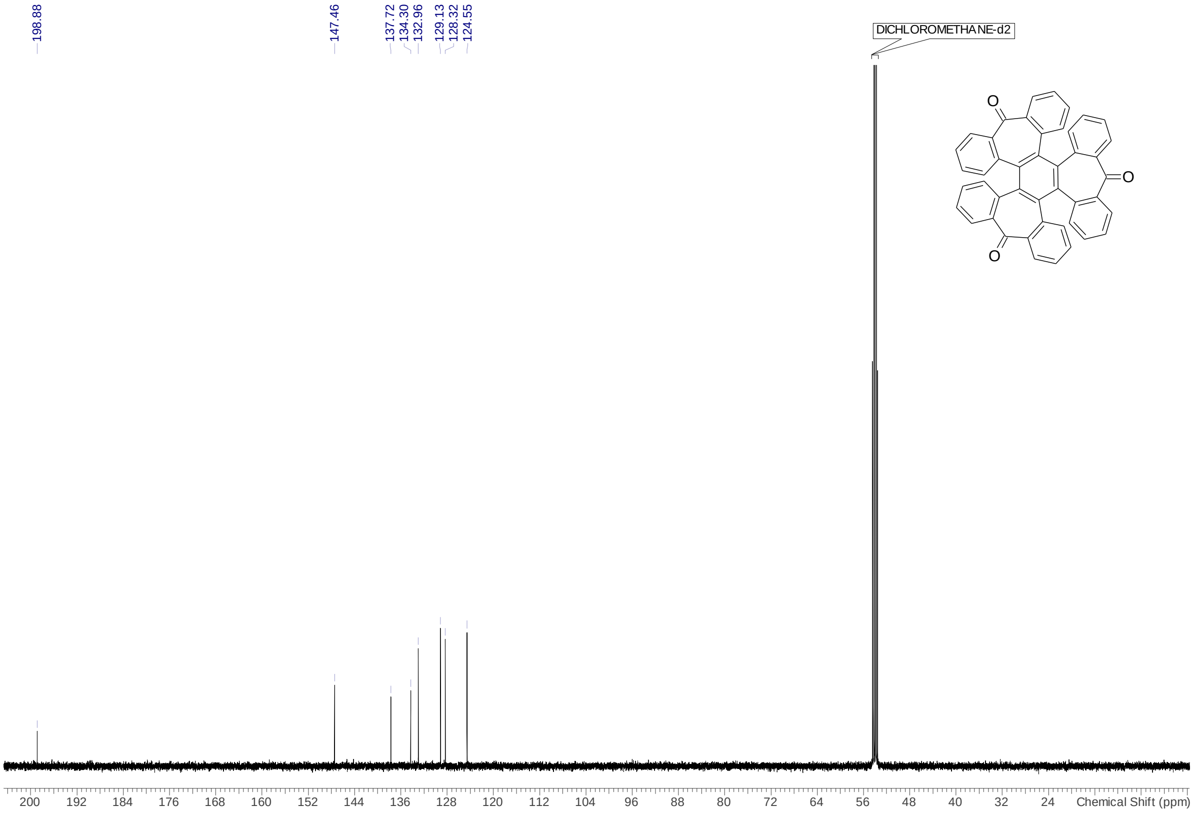

Figure S31: ^{13}C {^{1}H} NMR spectrum (126 MHz) of **2** in CD_2Cl_2.

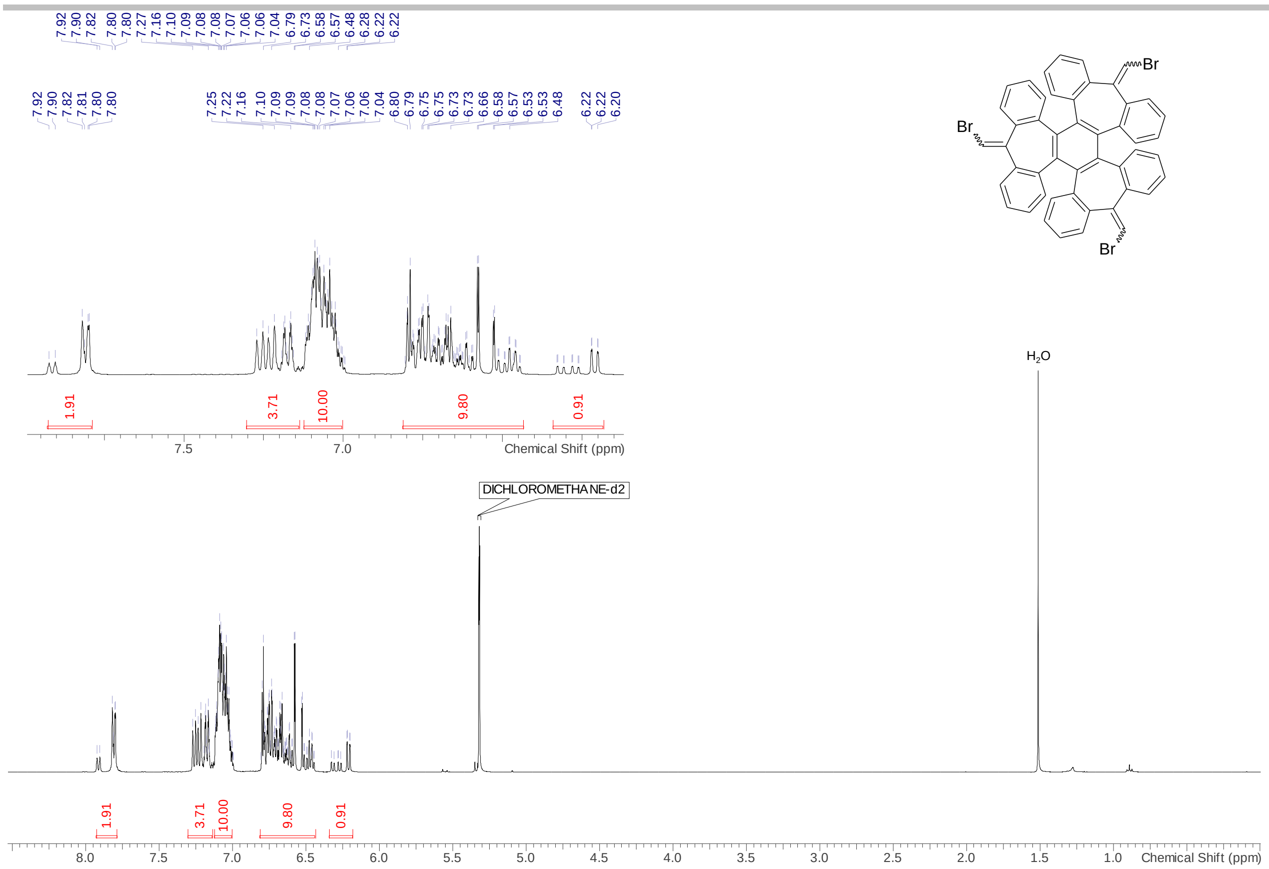

Figure S32: ^{1}H NMR spectrum (400 MHz) of **3** in CD_2Cl_2.

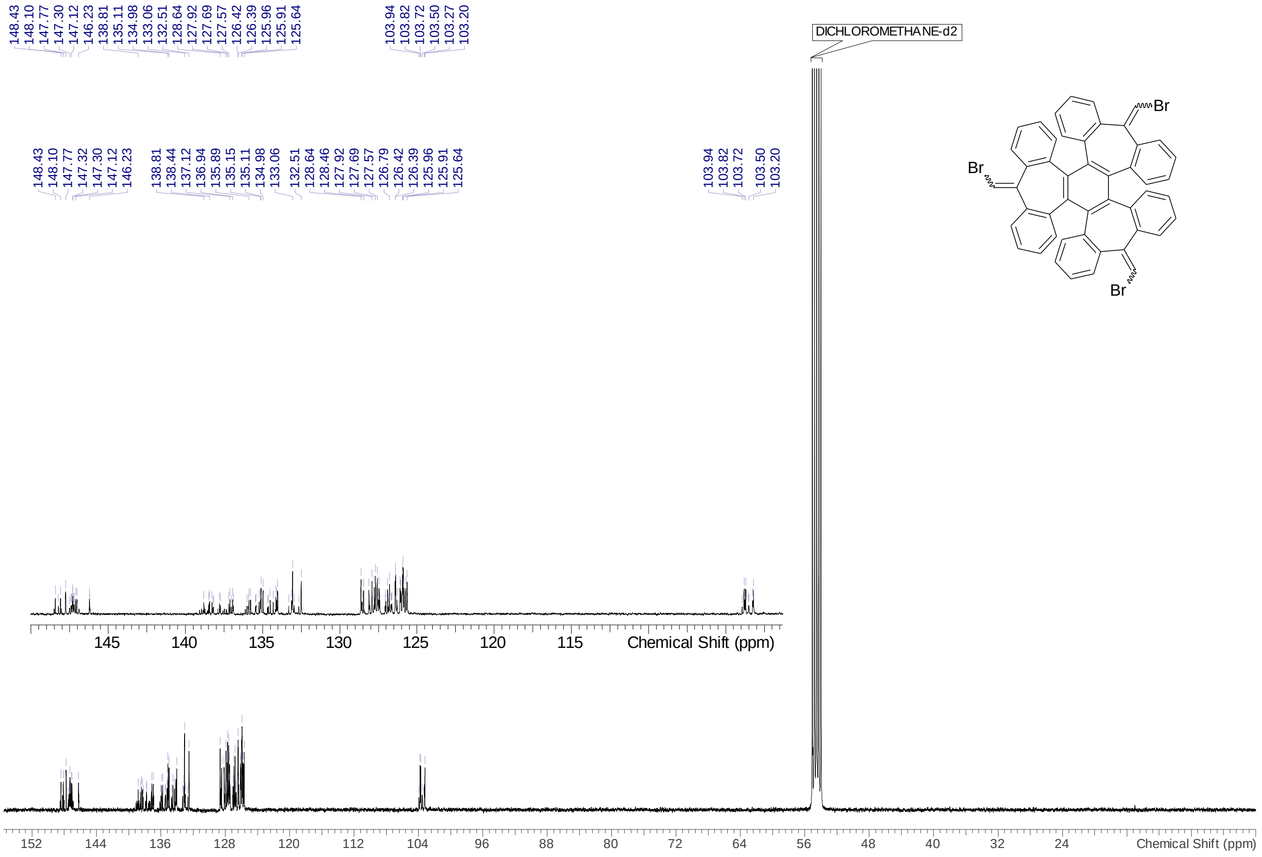

Figure 33: $^{13}C\{^{1}H\}$ NMR spectrum (101 MHz) of **3** in CD_2Cl_2.

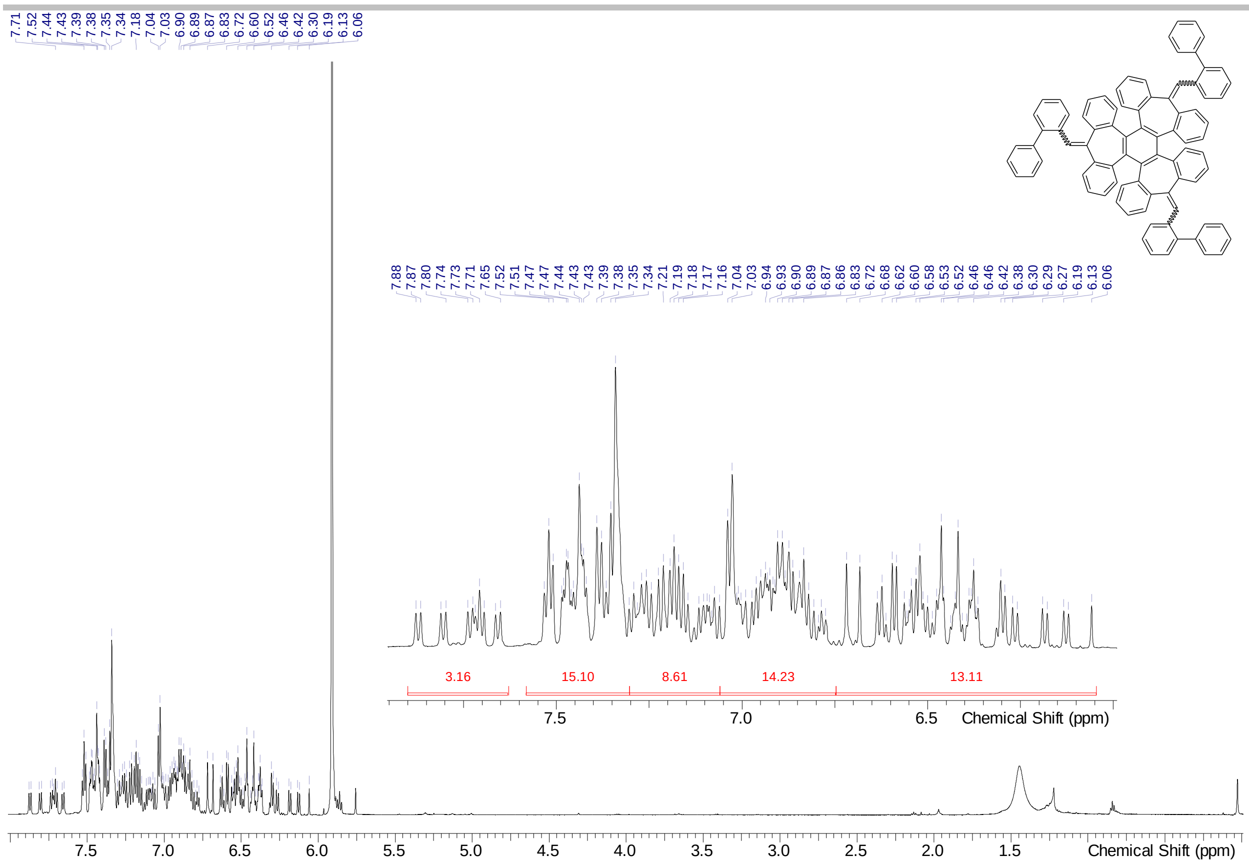

Figure S34: ^{1}H NMR spectrum (600 MHz, 70 °C) of **4** in $C_2D_2Cl_4$.

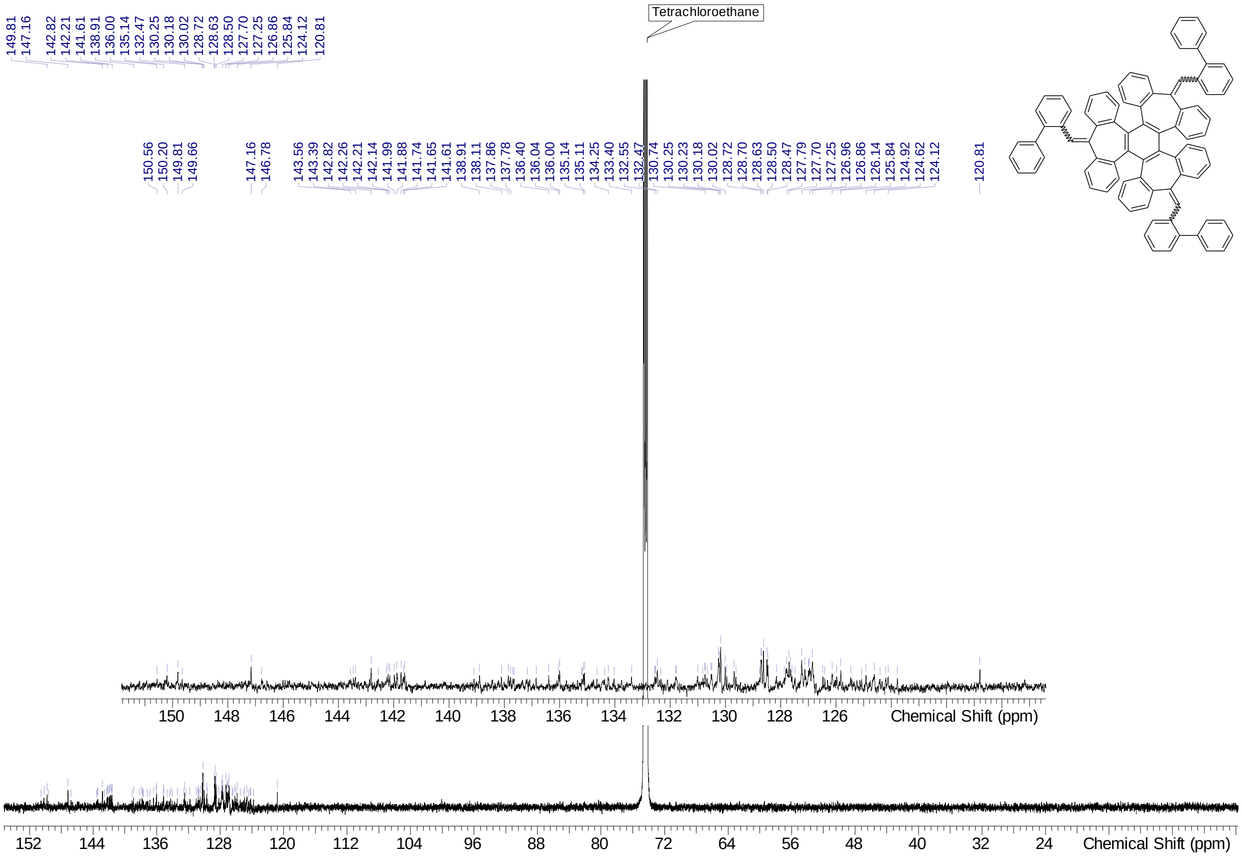

Figure S35: ^{13}C {^{1}H} NMR spectrum (151 MHz, 70 °C) of **4** in $C_2D_2Cl_4$.

2.3 Temperature-Dependent ^{1}H-NMR studies

We studied the feasibility of an (inter)conversion between cyclotrimers **7a** and **7b** experimentally. Temperature-dependent ^{1}H NMR in tetrachloroethane from -35 °C to 70 °C of **7a** (Figure S36) solely resulted in slight downfield shifts with increasing temperature – no sign of COT ring inversion was detected, which also was the case for **7b** (Figure S37). At -35 °C the ^{1}H NMR spectra showed broad low intensity peaks and ill-defined multiplicities due to viscosity increase. The resonance pattern of **7a** and of **7b** are temperature invariant, suggesting no conversion in this temperature range in solution.

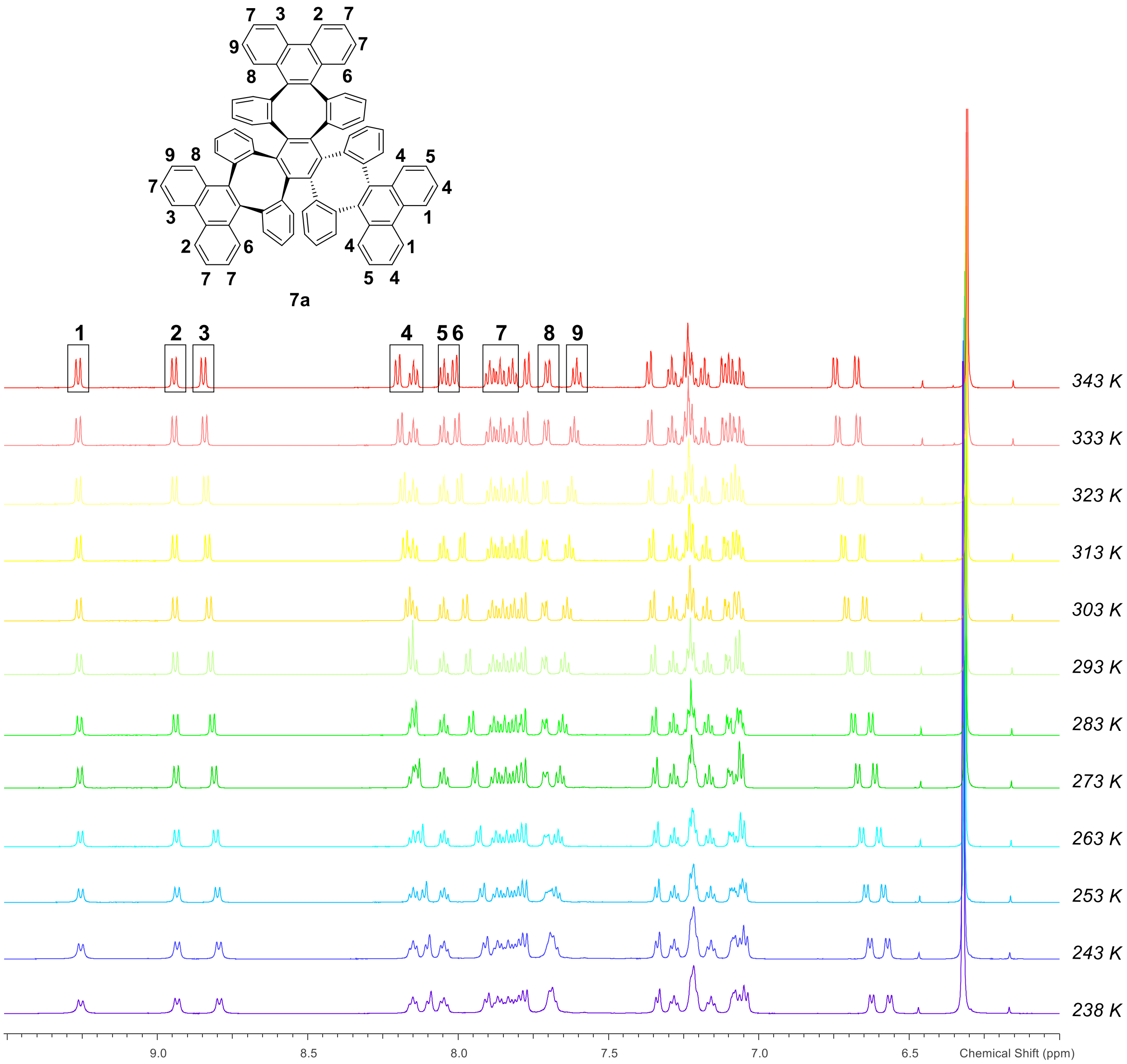

Figure S36: Temperature-dependent ^{1}H NMR spectra (600 MHz) of **7a** in $C_2D_2Cl_4$.

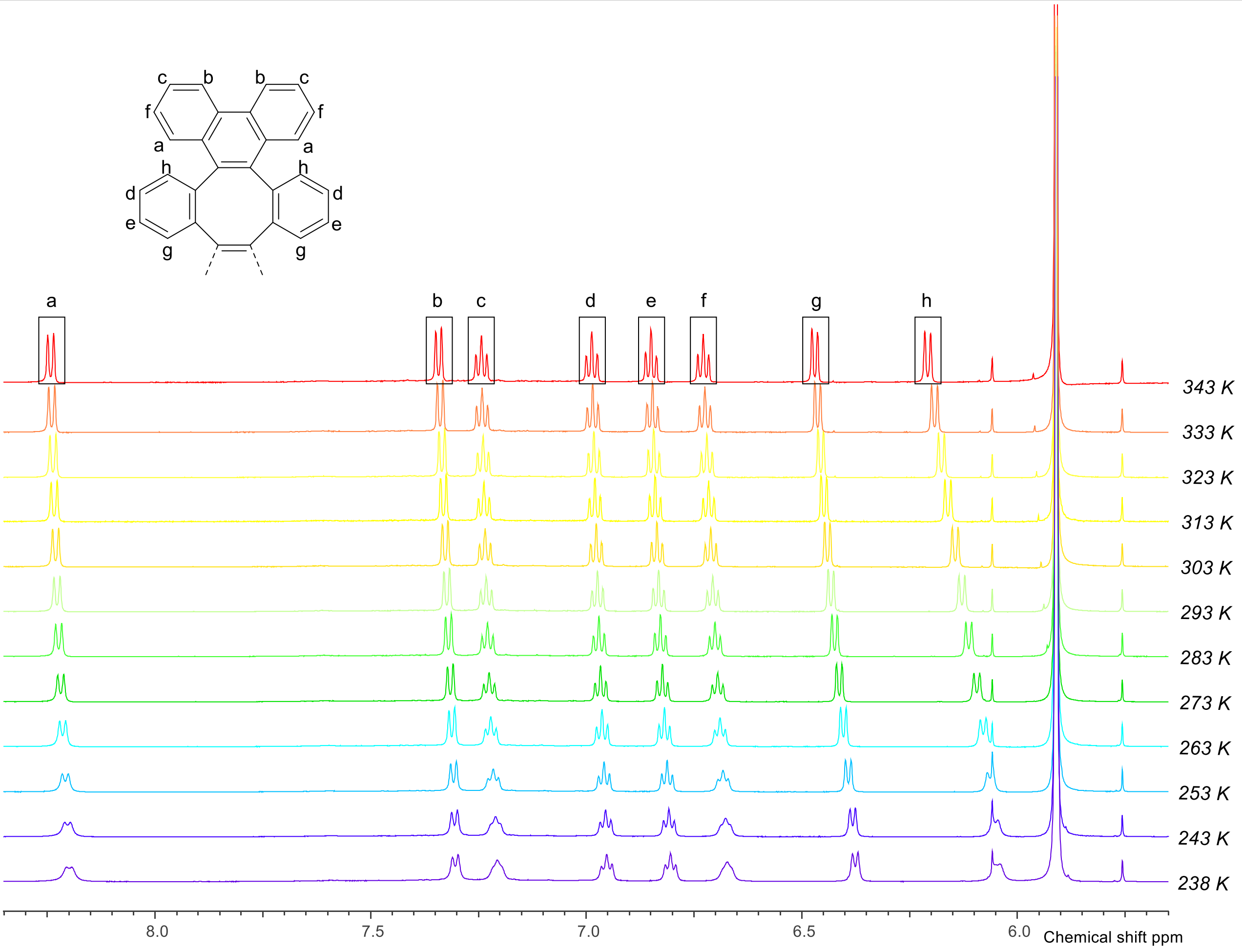

Figure S37: Temperature-dependent ^{1}H NMR spectra (600 MHz) of **7b** in $C_2D_2Cl_4$.

2.4 Absorption and Emission Spectra

All UV/vis and emission spectra were taken in dilute solutions (dichloromethane) in quartz cuvettes at room temperature. **7a**/**7b** show prominent absorption bands at 254/256 nm respectively, with shoulders at 300/304 nm and faint lowest wavelength absorption maxima at 356 and 358 nm (Figure S38). Their absorption spectra are quasi indiscernible as expected for conformers – **7b**'s absorption is slightly red-shifted by 2 nm. They fluoresce in the violet-blue regime at $\lambda_{max,em}$ = 360/365 nm – their emission spectra display well-resolved vibronic fine structures differing in intensity for the vibronic 0-0 transition. Stokes shifts are small (4 nm/312 cm^{-1} for **7a**, 7 nm/535 cm^{-1} for **7b**) due to the rigidity of both compounds consisting of 19 annulated six- or eight-membered rings.

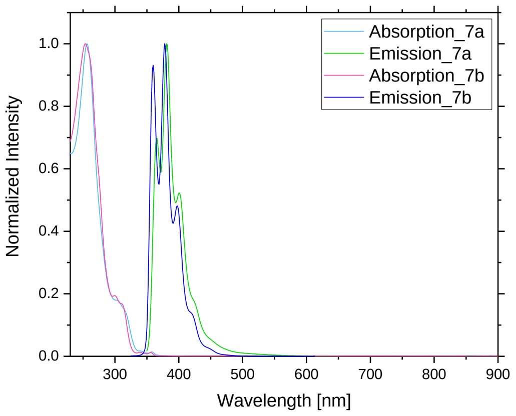

Figure S38: Absorption and Emission spectra of **S7a** and **S7b** in DCM (λ_{ex} = 311 nm for **7a** and 339 nm for **7b**).

2.5 Thermal and Light-Induced Stimuli

We attempted thermal isomerization in the bulk via thermogravimetric analysis. After keeping **7a** and **7b**, which show no sign of mass loss (only solvent evaporation) up to 500 °C and 475 °C (Figure S39) respectively, at 400 °C for 10 minutes, analysis via ^{1}H NMR and thin-layer chromatography indicated no (inter)conversion. Also photoirradiation of solutions of **7a** and **7b** in cyclohexane in a Rayonet photoreactor (300 nm) for 24 h provided only starting materials. Thus both isomers undergo neither a photo- nor a thermal isomerization, explicable by the steric congestion in both conformers.

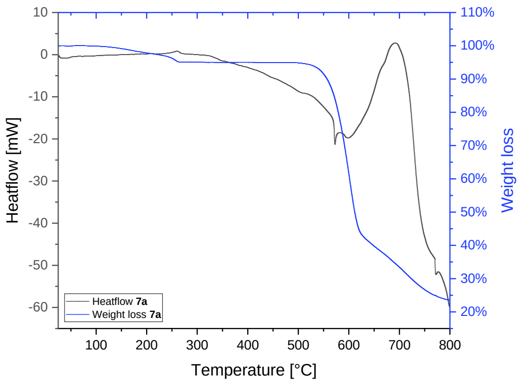

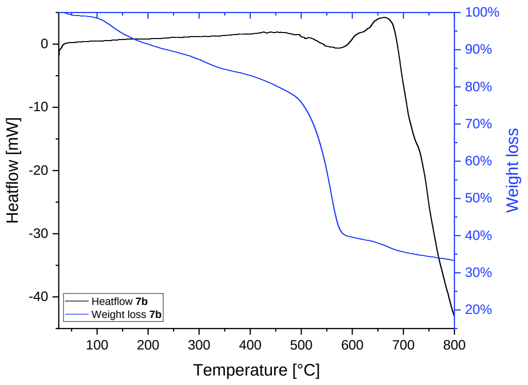

Figure S39: Thermogravimetric analysis and Differential Scanning Calorimetry of **7a** (left) and **7b** (right).

2.6 Determination of the Cavity Volume within 7b

The determination of the cavity volume was carried out using Mercury 4.2.0 or Chem3D 20.0. Method 1: One dummy atom was added to the **7b** and positioned in different levels of the cavity determining the 2.2 Å radius. The Van der Waals radius of the dummy atom was enlarged until it almost touched the space filling model of the phenanthrene-moieties of **7b** (Figure S40). Method 2: Another method to determine the volume was the solvent accessible surface via Chem3D, starting from 1.4 Å (water) and enlarging the radius until the triangle-like hole area disappeared (Figure S43), yielding a cavity volume of 2.7 Å. The triangle-shaped cavity spanned by the phenanthrenylene side walls should be suitable for host-guest-chemistry and can encompass e.g. a lithium ion (0.74 Å) [S10].

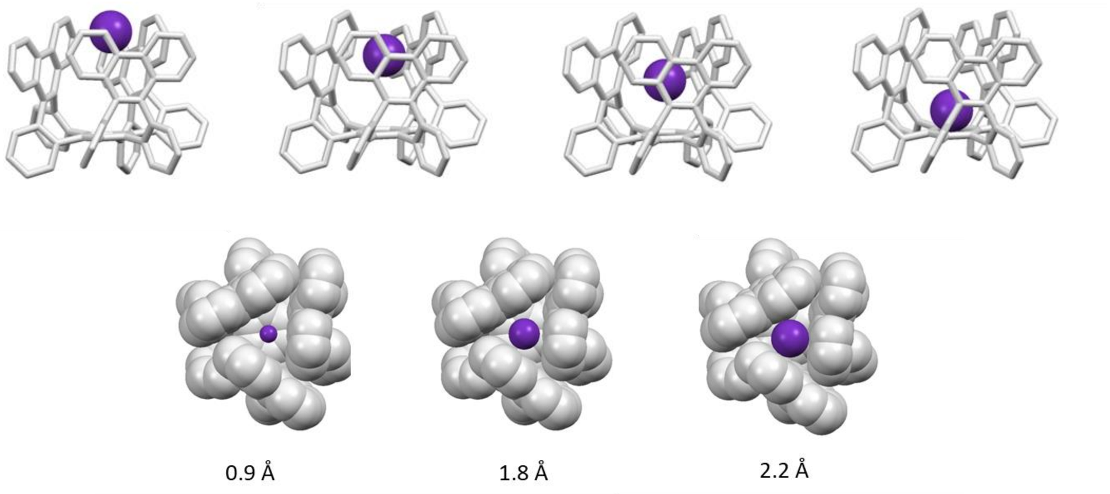

Figure S40: Insertion of a dummy atom into **7b** in different positions (top) and space-filling model of **7b** with different sizes of the dummy atom (bottom). Hydrogen-atoms were omitted for clarity.

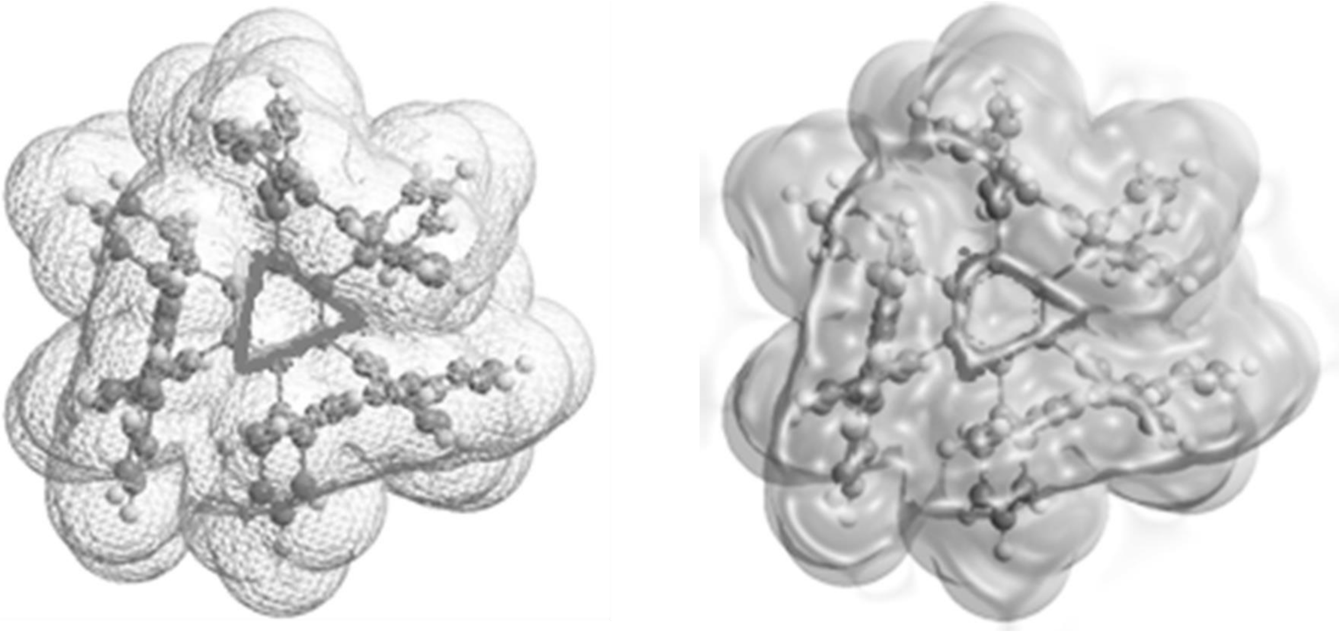

Figure S41: Illustration of solvent accessible surface area with a triangle-like cavity as (Chem3D) wire mash model (left) and as translucent model (right) (1.4 Å). The perimeter of the cavity was highlighted in grey.

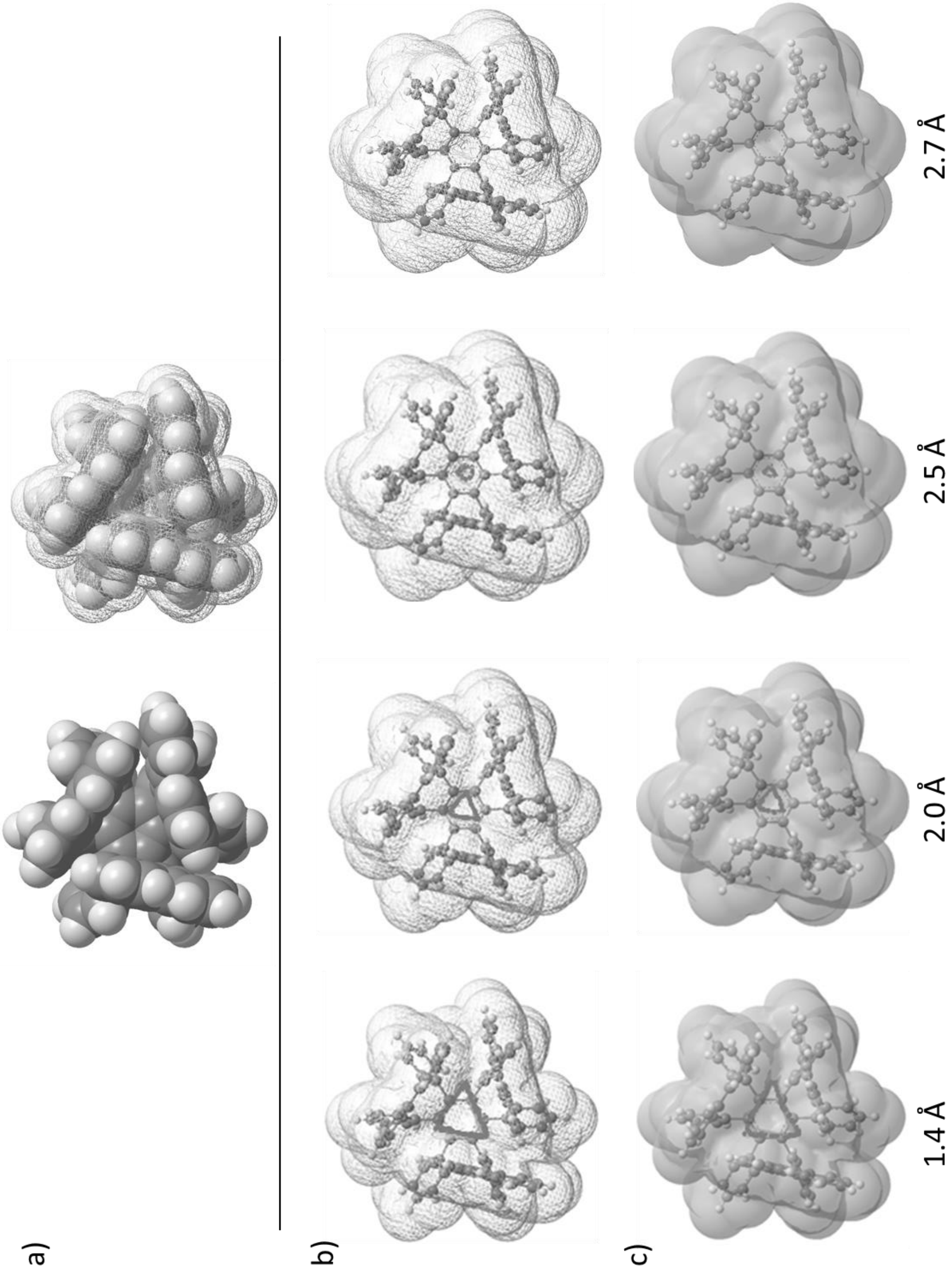

Figure S42: Illustration of solvent accessible surface area in different radius sizes. a) Space-filling model of **7b** (bottom) and with solvent surface area as wire mash model (top), ball and stick model of **7b** with solvent accessible surface area as (Chem3D) wire mash model (b) and as translucent model (c) from 1.4 – 2.7 Å. The perimeter of the cavity was highlighted in grey.

2.7 Calculations

Both cyclotrimers **7a** and **7b** were investigated also computationally, using Spartan 10 geometry optimizations (DFT, B3LYP/6-311G**). At this geometry, the absolute energies were assigned at the B3LYP/6-311G** level of theory. The α,α,β-isomer **7a** is by 68 kJ/mol more stable than the α,α,α-isomer **7b**.

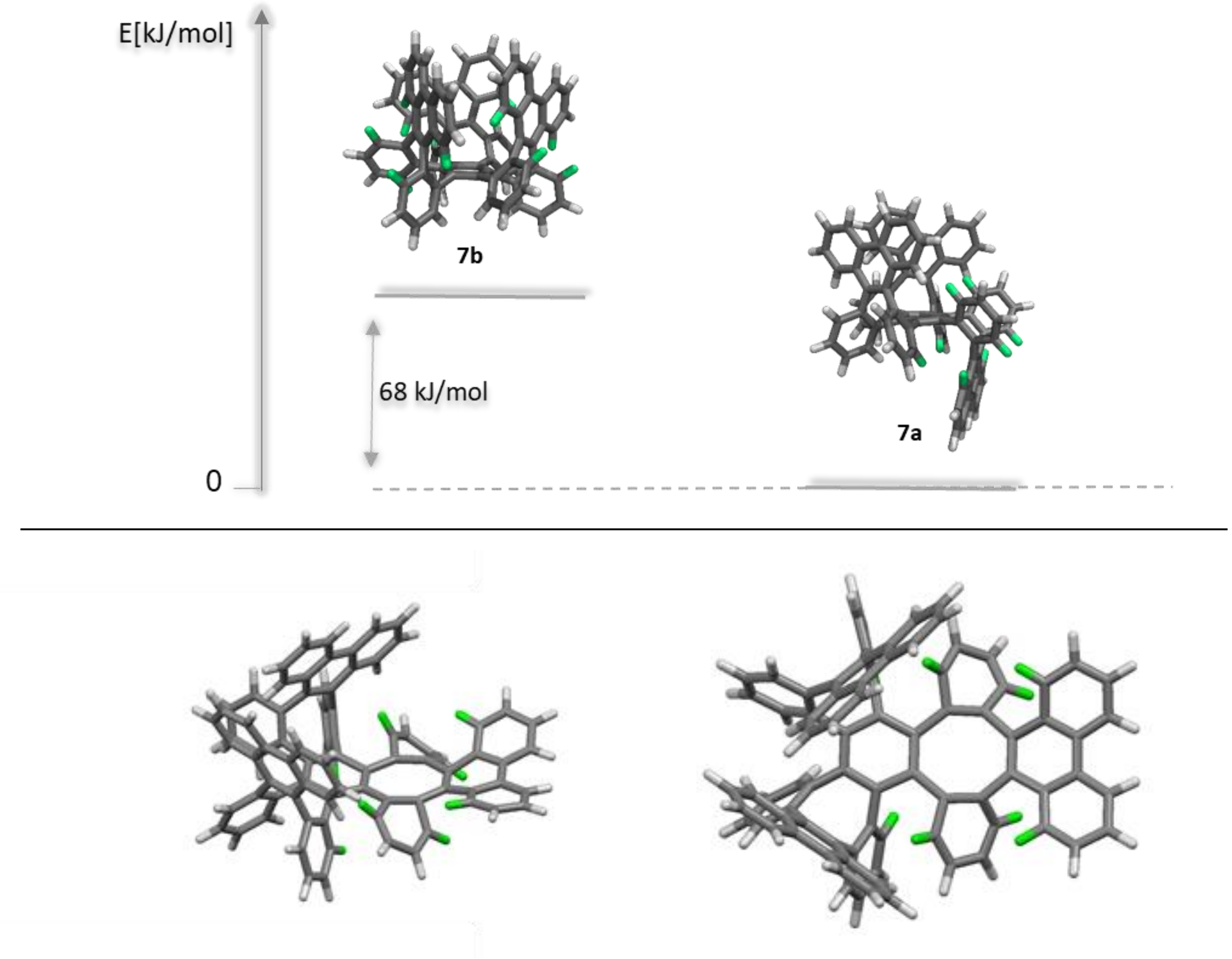

Figure S43: Top: Calculated absolute energies of the geometry optimized structures of **7a** (right), **7b** (left), bottom: structure of a partially planarized assumed transition state with only one planar COT moiety, side view (left) and top view (right). Green atoms indicate neighbouring protons in the overcrowded fjord regions. Note that the geometry of the assumed transition state was manually enforced and no result of automated optimization.

Isomer **7a**

Atom	x	y	z
C	1.17672	-0.670353	0.730328
C	1.290267	0.723546	0.474829
C	0.621735	1.294264	-0.648413
C	-0.232705	0.495578	-1.480543
C	-0.261545	-0.921595	-1.283659
C	0.43265	-1.483884	-0.166833
C	1.830483	-1.240604	1.943946
C	1.014464	-1.632092	3.023441
C	1.561233	-2.113738	4.212174
C	2.94045	-2.191791	4.35661
C	3.771637	-1.788572	3.311643
C	3.242409	-1.320901	2.089656
C	4.186115	-0.865253	1.021726
C	5.002728	-1.796683	0.315435
C	4.95052	-3.193472	0.574507
C	5.735455	-4.12012	-0.106455
C	6.60638	-3.67631	-1.078073
C	6.684465	-2.310984	-1.356444
C	5.90206	-1.332177	-0.681749
C	5.996958	0.074953	-0.968734
C	6.876548	0.607227	-1.953105
C	6.980934	1.972082	-2.22654
C	6.203182	2.865471	-1.521403
C	5.327643	2.383691	-0.552207
C	5.196332	1.001247	-0.250419
C	4.291077	0.530364	0.74323
C	3.477814	1.482151	1.552196
C	4.126384	2.254177	2.54103
C	3.408691	3.087073	3.396883
C	2.025484	3.141502	3.299965
C	1.365228	2.380066	2.334874
C	2.067655	1.56483	1.429272
C	0.889452	2.701983	-1.057592
C	2.083439	2.977559	-1.752856
C	2.358579	4.248822	-2.256011
C	1.430535	5.268826	-2.096868
C	0.231718	5.014542	-1.431904
C	-0.053123	3.742315	-0.89023
C	-1.358941	3.510375	-0.215437
C	-1.653155	4.15004	1.028782
C	-0.718347	4.997773	1.687105
C	-0.985888	5.630576	2.898389
C	-2.206949	5.429682	3.502956

C	-3.147003	4.604328	2.885165
C	-2.914399	3.948188	1.641978
C	-3.896317	3.122773	0.996786
C	-5.196701	2.925095	1.536606
C	-6.18001	2.18008	0.89159
C	-5.887232	1.58485	-0.315421
C	-4.610747	1.713555	-0.856508
C	-3.598243	2.490813	-0.244
C	-2.325213	2.677292	-0.854954
C	-2.036404	2.163931	-2.237705
C	-2.721036	2.798687	-3.30455
C	-2.442982	2.50519	-4.63606
C	-1.438397	1.601169	-4.935229
C	-0.737299	0.974092	-3.902937
C	-1.039984	1.187917	-2.541992
C	-0.848489	-1.874667	-2.280837
C	-0.079004	-2.161784	-3.433781
C	-0.476137	-3.10436	-4.382254
C	-1.636403	-3.831887	-4.178726
C	-2.379478	-3.625207	-3.018615
C	-2.011711	-2.654479	-2.053374
C	-2.798988	-2.604015	-0.784778
C	-4.176589	-2.239151	-0.788985
C	-4.846623	-1.84211	-1.977768
C	-6.221504	-1.628672	-2.03222
C	-6.969033	-1.764162	-0.880555
C	-6.328386	-2.073795	0.320239
C	-4.930104	-2.316047	0.413769
C	-4.281684	-2.658904	1.651775
C	-4.949499	-2.646119	2.907777
C	-4.329481	-3.012591	4.103845
C	-3.009099	-3.412385	4.089758
C	-2.319646	-3.431848	2.880749
C	-2.919476	-3.051977	1.650306
C	-2.204746	-3.07335	0.42243
C	-0.849816	-3.706538	0.342681
C	-0.80189	-5.057606	0.423458
C	0.441309	-5.777391	0.228007
C	1.554134	-5.107698	-0.106882
C	1.515182	-3.665442	-0.241855
C	0.389112	-2.965608	0.020231
H	-4.418508	1.192967	-1.792569
H	-6.644352	1.013351	-0.839717
H	-7.165775	2.072212	1.333778
H	-5.489233	3.372363	2.481194
H	-4.088056	4.486205	3.413763

H	-2.437875	5.906973	4.451038
H	-0.235509	6.265163	3.360636
H	0.270472	5.164314	1.262208
H	0.277817	2.39366	2.309455
H	1.451291	3.75803	3.985847
H	3.927372	3.6667	4.155053
H	5.206451	2.184635	2.658059
H	4.720382	3.122948	-0.031283
H	6.266689	3.931649	-1.71857
H	7.670307	2.325024	-2.988508
H	7.517095	-0.04231	-2.542183
H	7.39128	-2.027348	-2.130504
H	7.227881	-4.381212	-1.623267
H	5.657314	-5.178281	0.125601
H	4.266743	-3.591504	1.322862
H	-0.066054	-1.53396	2.940872
H	0.907468	-2.407024	5.028694
H	3.372338	-2.549923	5.28698
H	4.850359	-1.830364	3.451995
H	-1.277123	-3.745054	2.910884
H	-2.509295	-3.703511	5.008921
H	-4.88417	-2.981435	5.037434
H	-5.985516	-2.329629	2.98554
H	-6.967725	-2.150335	1.194746
H	-8.04365	-1.606402	-0.900516
H	-6.694555	-1.355598	-2.97101
H	-4.292725	-1.708494	-2.905715
H	-3.251957	-4.253748	-2.843986
H	-1.945058	-4.586275	-4.896613
H	0.139288	-3.28713	-5.258476
H	0.876754	-1.659908	-3.578132
H	-0.498633	5.816408	-1.338222
H	1.62823	6.256875	-2.502771
H	3.286808	4.429838	-2.790513
H	2.797896	2.175926	-1.929892
H	-3.470008	3.559027	-3.08601
H	-2.98284	3.010824	-5.431244
H	-1.177409	1.395344	-5.969624
H	0.092449	0.334251	-4.182229
H	-1.69357	-5.652834	0.597726
H	0.438553	-6.856212	0.334486
H	2.495132	-5.617392	-0.278876
H	2.422733	-3.172929	-0.571355

Isomer **7b**

Atom	x	y	z
C	-0.790157	1.179149	-1.881008
C	-1.361424	-0.124352	-1.997368
C	-0.544991	-1.293369	-1.914215
C	0.87572	-1.140311	-1.973233
C	1.47843	0.148082	-1.866238
C	0.628579	1.292758	-1.928647
C	-1.705985	2.363167	-1.715293
C	-1.692511	3.399716	-2.674329
C	-2.508952	4.528348	-2.576726
C	-3.403703	4.641553	-1.528272
C	-3.494869	3.611672	-0.596894
C	-2.668263	2.459208	-0.658149
C	-2.984449	1.37362	0.345321
C	-2.833641	1.590338	1.745894
C	-2.163747	2.728127	2.256106
C	-2.05926	2.990255	3.620604
C	-2.593654	2.101838	4.528008
C	-3.244052	0.962728	4.060976
C	-3.398481	0.672627	2.678442
C	-4.101154	-0.490426	2.214926
C	-4.770776	-1.399701	3.083259
C	-5.406548	-2.558981	2.633847
C	-5.383038	-2.864323	1.290052
C	-4.767802	-1.981074	0.407546
C	-4.159136	-0.765911	0.829559
C	-3.637785	0.184159	-0.098345
C	-3.862553	-0.005777	-1.549121
C	-5.174305	0.072715	-2.068381
C	-5.422841	-0.025024	-3.436342
C	-4.360886	-0.171116	-4.316721
C	-3.057991	-0.226428	-3.821816
C	-2.785277	-0.177473	-2.434948
C	-1.117002	-2.678736	-1.758998
C	-1.969936	-3.202526	-2.754929
C	-2.540841	-4.473743	-2.665553
C	-2.243343	-5.287545	-1.587381
C	-1.353841	-4.835091	-0.61753
C	-0.769026	-3.543011	-0.670709
C	0.281634	-3.255236	0.377126
C	-0.045361	-3.206139	1.763113
C	-1.387808	-3.191734	2.211919
C	-1.728322	-3.208166	3.562801
C	-0.734165	-3.201969	4.516697

C	0.597434	-3.196628	4.10995
C	0.988602	-3.211768	2.743948
C	2.367213	-3.242528	2.343343
C	3.449125	-3.343007	3.264447
C	4.79001	-3.324682	2.874553
C	5.103395	-3.18487	1.539698
C	4.07191	-3.114885	0.607724
C	2.697559	-3.186526	0.969825
C	1.657433	-3.232095	-0.005304
C	1.999073	-3.357742	-1.440537
C	2.607711	-4.541871	-1.912938
C	2.874211	-4.732253	-3.267734
C	2.507753	-3.755427	-4.182171
C	1.884751	-2.590584	-3.734284
C	1.648525	-2.354373	-2.360387
C	2.959105	0.345796	-1.671007
C	3.870132	-0.141767	-2.632806
C	5.253003	0.000125	-2.499919
C	5.774328	0.676479	-1.411614
C	4.907216	1.231785	-0.475555
C	3.498809	1.090799	-0.57355
C	2.685066	1.867684	0.432396
C	2.764993	1.59861	1.828494
C	3.426186	0.451597	2.329316
C	3.569195	0.202478	3.692623
C	3.022208	1.076926	4.606408
C	2.34792	2.205827	4.147944
C	2.205138	2.513664	2.768319
C	1.535894	3.70184	2.314917
C	1.041328	4.708406	3.192902
C	0.352331	5.839519	2.749004
C	0.115246	6.001283	1.400601
C	0.611004	5.054049	0.509665
C	1.359453	3.918752	0.929814
C	1.961672	3.01801	0.006778
C	1.958697	3.361654	-1.442942
C	2.734451	4.511833	-1.935992
C	2.826026	4.783802	-3.246903
C	2.172236	3.922483	-4.20317
C	1.464747	2.866128	-3.772891
C	1.311957	2.567738	-2.334672
H	-5.843379	-3.77515	0.918926
H	-5.895782	-3.223219	3.340486
H	-4.809345	-1.222202	4.153882
H	-4.766193	-2.261427	-0.644984
H	-3.628279	0.29151	4.822401

H	-2.504364	2.283546	5.594696
H	-1.554065	3.884728	3.966389
H	-1.701265	3.441833	1.577936
H	1.184516	4.63059	4.266556
H	-0.009822	6.573908	3.46279
H	-0.442888	6.857167	1.032929
H	0.400174	5.214361	-0.546886
H	1.922984	2.847194	4.913529
H	3.109772	0.885953	5.671654
H	4.101163	-0.678931	4.032048
H	3.846288	-0.282586	1.645208
H	4.361379	-2.998185	-0.435815
H	6.138139	-3.136784	1.213752
H	5.576998	-3.398198	3.619628
H	3.266508	-3.437981	4.330654
H	1.335173	-3.17395	4.905642
H	-0.984699	-3.195607	5.573092
H	-2.770066	-3.218417	3.861551
H	-2.206114	-3.1654	1.495531
H	-1.086349	-5.512302	0.193208
H	-1.054202	3.328935	-3.547799
H	-2.451575	5.304746	-3.334548
H	-4.0538	5.507819	-1.449581
H	-4.245183	3.692697	0.189226
H	-6.012693	0.230913	-1.391766
H	-2.232613	-0.295968	-4.528525
H	-4.53998	-0.219189	-5.387091
H	-6.440883	0.039155	-3.81
H	-2.670127	-6.283442	-1.514343
H	-3.201708	-4.826757	-3.452328
H	-2.182419	-2.62947	-3.650388
H	1.563196	-1.853598	-4.468356
H	2.684118	-3.905729	-5.243494
H	3.342527	-5.652398	-3.605648
H	2.860044	-5.334862	-1.210698
H	5.333467	1.812825	0.341727
H	6.847311	0.806166	-1.305915
H	5.914061	-0.403757	-3.261664
H	3.508967	-0.620554	-3.535835
H	0.996777	2.190645	-4.482136
H	2.289406	4.133282	-5.26011
H	3.403652	5.626303	-3.610026
H	3.243967	5.132837	-1.205572

2.8 Crystallographic Data

Brick-shaped colorless single crystals were obtained by slow evaporation of a $CHCl_3$/MeOH solution of **7a**. Four molecules of **7a** co-crystallize with 16 additional $CHCl_3$ molecules (space group $P2_1/c$) per unit cell. Plank-shaped, pale yellow single crystals of **7b** were cultivated by slow evaporation of a concentrated THF solution - **7b** (space group C_2/c) co-crystallizes with three additional THF molecules (Z = 8). Due to steric congestions, the phenanthrenylenes in **7b** deviate from planarity – torsion angles between carbons ∢ chc'h' (see Figure S37) range between 10.1° to 12.7°. Within one molecule, each edge of one phenanthrylene is directed to the face of an adjacent one, thus giving rise to λ- and δ-enantiomers in the single crystal, which are oriented with their cavity pairwise down- and upwards.

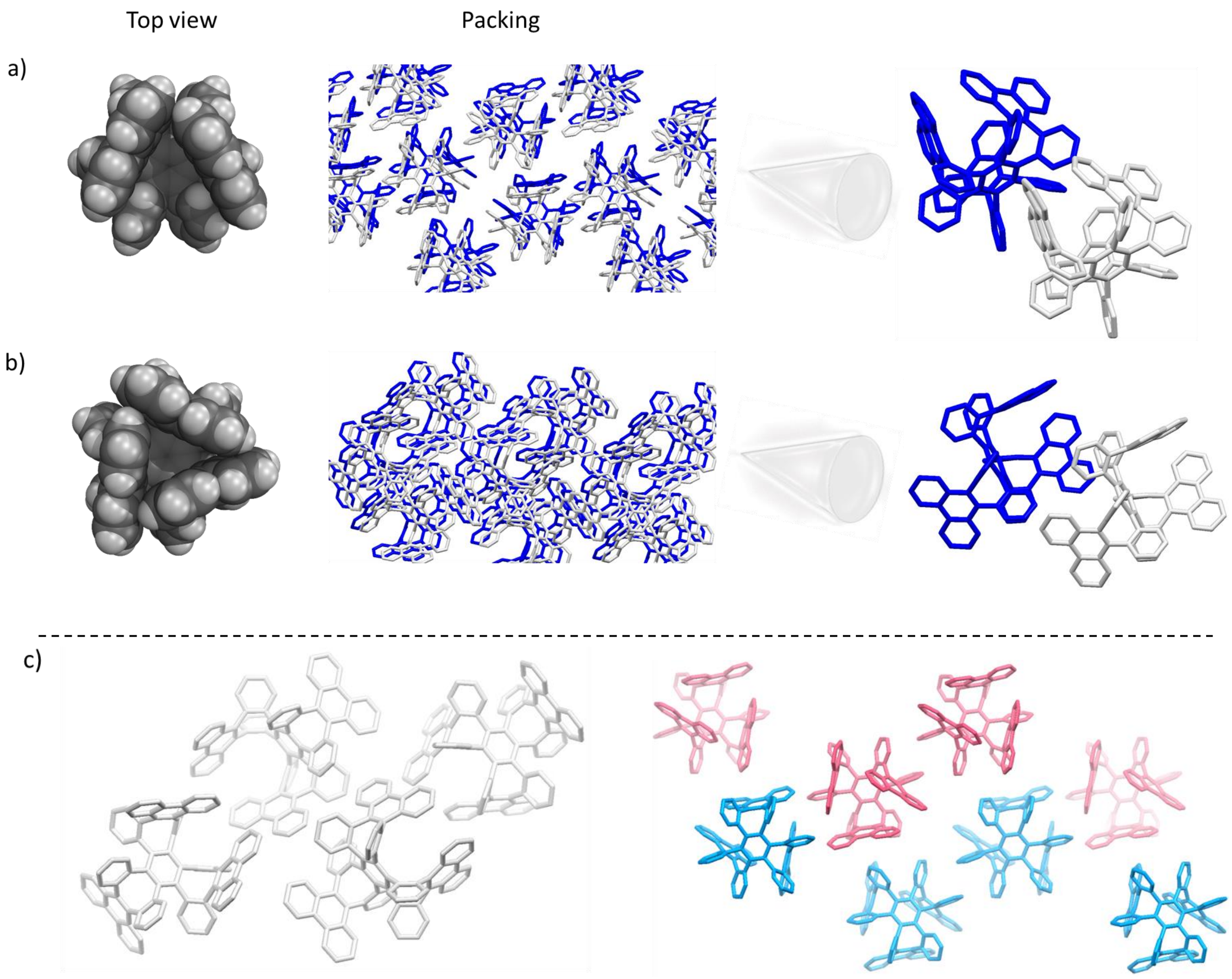

Figure S44: Solid state structures as space-filling model and packing of **7a** (a) and **7b** (b), unit cell (c) of **7a** (left) and **7b** (right), molecules were color-coded with respect to the orientation of the three phenanthrylene moieties. Pink: upwards, blue: downwards. Solvent molecules were omitted for clarity.

SUPPORTING INFORMATION

Table S2: Crystal structure, crystal data and structure refinement for **7a** (CCDC: 2080525).

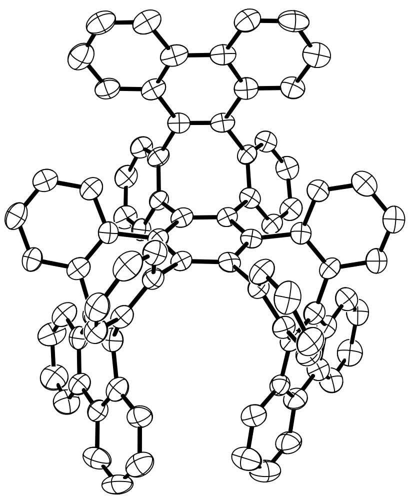

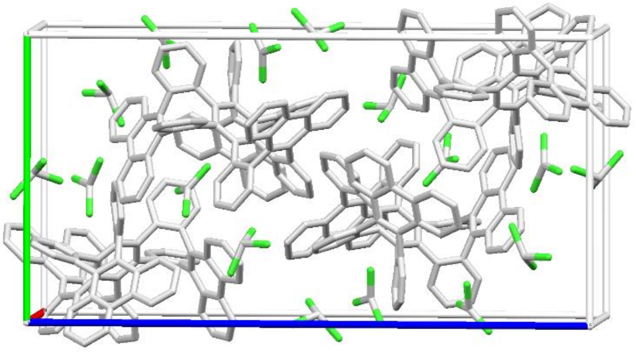

Empirical formula	$C_{88}H_{52}Cl_{12}$	
Formula weight	1534.69	
Temperature	200(2) K	
Wavelength	1.54178 Å	
Crystal system	monoclinic	
Space group	$P2_1/c$	
Z	4	
Unit cell dimensions	a = 14.5431(7) Å	α = 90 °.
	b = 15.6779(5) Å	β = 98.953(4) °.
	c = 29.8695(12) Å	γ = 90 °.
Volume	6727.4(5) $Å^3$	
Density (calculated)	1.51 g/cm^3	
Absorption coefficient	4.93 mm^{-1}	
Crystal shape	brick	
Crystal size	0.102 x 0.052 x 0.029 mm^3	
Crystal colour	colourless	
Theta range for data collection	4.1 to 68.3 °.	
Index ranges	$-15\leq h\leq 16$, $-17\leq k\leq 13$, $-27\leq l\leq 35$	
Reflections collected	28574	
Independent reflections	7941 (R(int) = 0.1522)	
Observed reflections	4319 (I > 2σ(I))	
Absorption correction	Semi-empirical from equivalents	
Max. and min. transmission	1.00 and 0.43	
Refinement method	Full-matrix least-squares on F^2	
Data/restraints/parameters	7941 / 759 / 901	
Goodness-of-fit on F^2	1.02	
Final R indices (I>2sigma(I))	R1 = 0.099, wR2 = 0.264	
Largest diff. peak and hole	0.87 and -0.55 $eÅ^{-3}$	

Table S3: Crystal structure, crystal data and structure refinement for **7b** (CCDC: 2080526).

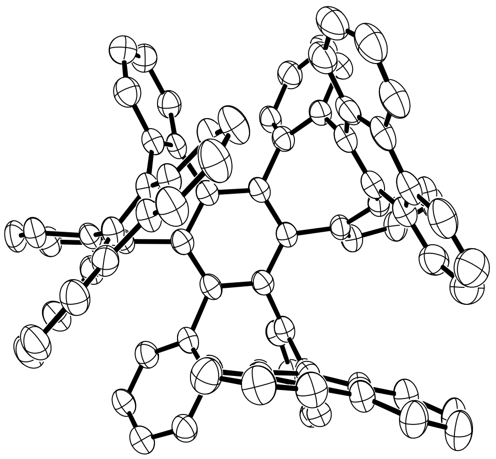

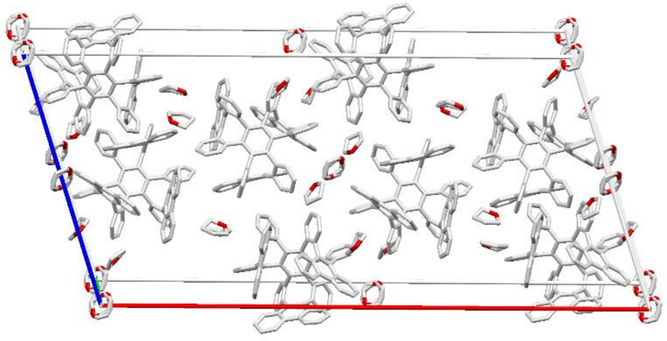

Empirical formula	$C_{96}H_{72}O_3$	
Formula weight	1273.53	
Temperature	200(2) K	
Wavelength	1.54178 Å	
Crystal system	monoclinic	
Space group	C2/c	
Z	8	
Unit cell dimensions	a = 51.1347(18) Å	α = 90 °.
	b = 11.0185(2) Å	β = 107.776(3) °.
	c = 25.2123(9) Å	γ = 90 °.
Volume	13527.1(8) $Å^3$	
Density (calculated)	1.25 g/cm^3	
Absorption coefficient	0.57 mm^{-1}	
Crystal shape	plank	
Crystal size	0.078 x 0.067 x 0.038 mm^3	
Crystal colour	pale yellow	
Theta range for data collection	4.1 to 57.3 °.	
Index ranges	$-55 \leq h \leq 55$, $-11 \leq k \leq 6$, $-27 \leq l \leq 26$	
Reflections collected	43451	
Independent reflections	8990 (R(int) = 0.0703)	
Observed reflections	5337 (I > 2σ(I))	
Absorption correction	Semi-empirical from equivalents	
Max. and min. transmission	1.45 and 0.70	
Refinement method	Full-matrix least-squares on F^2	
Data/restraints/parameters	8990 / 1584 / 915	
Goodness-of-fit on F^2	0.98	
Final R indices (I>2sigma(I))	R1 = 0.058, wR2 = 0.136	
Largest diff. peak and hole	0.26 and -0.28 $eÅ^{-3}$	

Table S4: Crystal structure, crystal data and structure refinement for **3** (CCDC: 2080527).

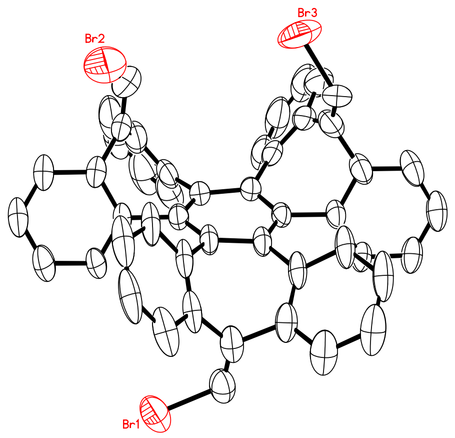

Empirical formula	$C_{49}H_{28}Br_3Cl_3$	
Formula weight	962.79	
Temperature	200(2) K	
Wavelength	0.71073 Å	
Crystal system	triclinic	
Space group	P $\bar{1}$	
Z	2	
Unit cell dimensions	a = 10.5054(5) Å	α = 108.6828(13) °.
	b = 13.7386(7) Å	β = 103.9446(13) °.
	c = 15.4406(8) Å	γ = 92.9724(13) °.
Volume	2028.60(18) $Å^3$	
Density (calculated)	1.58 g/cm^3	
Absorption coefficient	3.22 mm^{-1}	
Crystal shape	plank	
Crystal size	0.176 x 0.154 x 0.045 mm^3	
Crystal colour	colourless	
Theta range for data collection	1.4 to 24.1 °.	
Index ranges	$-12 \le h \le 11$, $-15 \le k \le 15$, $-17 \le l \le 17$	
Reflections collected	24112	
Independent reflections	6452 (R(int) = 0.0330)	
Observed reflections	4289 (I > 2σ(I))	
Absorption correction	Semi-empirical from equivalents	
Max. and min. transmission	0.90 and 0.77	
Refinement method	Full-matrix least-squares on F^2	
Data/restraints/parameters	6452 / 1397 / 562	
Goodness-of-fit on F^2	1.05	
Final R indices (I>2sigma(I))	R1 = 0.085, wR2 = 0.212	
Largest diff. peak and hole	1.13 and -1.23 $eÅ^{-3}$	

References

[S1] G. R. Fulmer, A. J. M. Miller, N. H. Sherden, H. E. Gottlieb, A. Nudelman, B. M. Stoltz, J. E. Bercaw, K. I. Goldberg, *Organometallics* **2010**, *29*, 2176-2179.
[S2] M. J. Frisch, G. W. Trucks, H. B. Schlegel, G. E. Scuseria, M. A. Robb, J. R. Cheeseman, G. Scalmani, V. Barone, G. A. Petersson, H. Nakatsuji, X. Li, M. Caricato, A. V. Marenich, J. Bloino, B. G. Janesko, R. Gomperts, B. Mennucci, H. P. Hratchian, J. V. Ortiz, A. F. Izmaylov, J. L. Sonnenberg, Williams, F. Ding, F. Lipparini, F. Egidi, J. Goings, B. Peng, A. Petrone, T. Henderson, D. Ranasinghe, V. G. Zakrzewski, J. Gao, N. Rega, G. Zheng, W. Liang, M. Hada, M. Ehara, K. Toyota, R. Fukuda, J. Hasegawa, M. Ishida, T. Nakajima, Y. Honda, O. Kitao, H. Nakai, T. Vreven, K. Throssell, J. A. Montgomery Jr., J. E. Peralta, F. Ogliaro, M. J. Bearpark, J. J. Heyd, E. N. Brothers, K. N. Kudin, V. N. Staroverov, T. A. Keith, R. Kobayashi, J. Normand, K. Raghavachari, A. P. Rendell, J. C. Burant, S. S. Iyengar, J. Tomasi, M. Cossi, J. M. Millam, M. Klene, C. Adamo, R. Cammi, J. W. Ochterski, R. L. Martin, K. Morokuma, O. Farkas, J. B. Foresman, D. J. Fox, Wallingford, CT, **2016**.
[S3] W. H. a. S. Ohlinger, *Wavefunction Inc, USA* **2010**.
[S4] L. Krause, R. Herbst-Irmer, G. M. Sheldrick, D. Stalke, *Journal of Applied Crystallography* **2015**, *48*, 3-10.
[S5] G. Sheldrick, *Acta Crystallographica Section A* **2015**, *71*, 3-8.
[S6] G. Sheldrick, *Acta Crystallographica Section C* **2015**, *71*, 3-8.
[S7] L. Yang, H. Matsuyama, S. Zhang, M. Terada, T. Jin, *Organic Letters* **2020**, *22*, 5121-5125.
[S8] S. C. Kornmayer, F. Rominger, R. Gleiter, *Synthesis* **2009**, *2009*, 2547-2552.
[S9] J. Klosin, K. A. Abboud, W. M. Jones, *Organometallics* **1995**, *14*, 2892-2902.
[S10] C. Eames, J. M. Clark, G. Rousse, J.-M. Tarascon, M. S. Islam, *Chemistry of Materials* **2014**, *26*, 3672-3678.
